# Visible light photoredox-catalyzed deoxygenation of alcohols

**DOI:** 10.3762/bjoc.10.223

**Published:** 2014-09-10

**Authors:** Daniel Rackl, Viktor Kais, Peter Kreitmeier, Oliver Reiser

**Affiliations:** 1 Institute of Organic Chemistry, University of Regensburg, 93053 Regensburg, Germany

**Keywords:** C–O bond activation, deoxygenation, photochemistry, photoredox catalysis, visible light

## Abstract

Carbon–oxygen single bonds are ubiquitous in natural products whereas efficient methods for their reductive defunctionalization are rare. In this work an environmentally benign protocol for the activation of carbon–oxygen single bonds of alcohols towards a reductive bond cleavage under visible light photocatalysis was developed. Alcohols were activated as 3,5-bis(trifluoromethyl)-substituted benzoates and irradiation with blue light in the presence of [Ir(ppy)_2_(dtb-bpy)](PF_6_) as visible light photocatalyst and Hünig’s base as sacrificial electron donor in an acetonitrile/water mixture generally gave good to excellent yields of the desired defunctionalized compounds. Functional group tolerance is high but the protocol developed is limited to benzylic, α-carbonyl, and α-cyanoalcohols; with other alcohols a slow partial C–F bond reduction in the 3,5-bis(trifluoromethyl)benzoate moiety occurs.

## Introduction

The dwindling supply of hydrocarbons from fossil resources calls for the usage of renewable resources for the synthesis of fine chemicals in the future [1]. This strategy suffers from the relative high degree of functionalization of feedstock materials, which is often not desired in fine chemicals and further leads to compatibility issues in chemical transformations. Carbon–oxygen single bonds are common elements in natural materials and their reduction to non-functionalized carbon–hydrogen bonds decreases complexity and increases compatibility of those materials in further chemical manipulations in accordance with established oil-based protocols developed in the chemical industry during the last century.

**Scheme 1:**
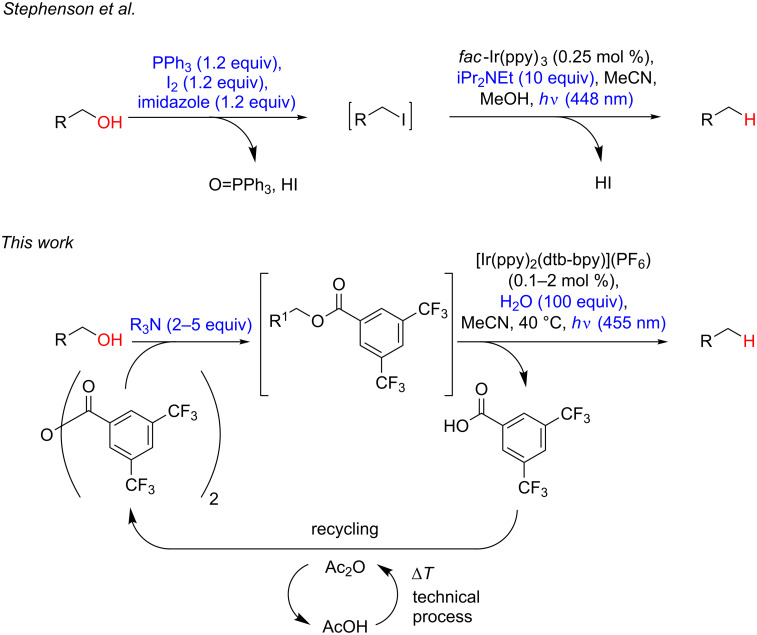
Strategies for the visible light-catalysed deoxygenation of alcohols (reagents needed in (over-)stoichiometric quantities are depicted in blue).

A classical radical deoxygenation reaction using over-stoichiometric amounts of highly noxious chemicals [2] is the Barton–McCombie reaction, although nowadays several improved protocols are available [3]. Radical deoxygenations can also be carried out electrochemically [4] or photochemically [5–9]. In all these cases an activation of the hydroxy group is necessary, either via conversion to the corresponding halide or formation of an ester derivative, which consequently generates over-stoichiometric amounts of byproducts. Related to this work, Stephenson et al. elegantly succeeded in the direct deoxygenation of alcohols by their in situ conversion to iodides using triphenylphospine and iodine followed by visible light-mediated reduction with amines as stoichiometric sacrificial electron donor and *fac*-Ir(ppy)_3_ (ppy = 2-phenylpyridine) as photoredox catalyst (Scheme 1) [10]. This protocol is applicable to a broad range of alcohols. It also greatly advanced the quest to develop sustainable methods for the deoxygenation of alcohols. Yet, several redox steps, i.e., the stoichiometric transformation of triphenylphosphine to triphenylphospine oxide and iodine to iodide, are required, which appears to be problematic for establishing a sustainable protocol that allows the recycling and reuse of the reagents involved. Herein we report a redox economic deoxygenation method of alcohols in which formation of radicals is achieved under visible light photocatalysis and the auxiliary activation group can be readily recovered and reused.

This method, ultimately requiring only energy and a tertiary amine as stoichiometric reductant, gives high yields after short illumination times under mild conditions for the deoxygenation of benzylic alcohols, α-hydroxycarbonyl, and α-cyanohydrin compounds. Moreover, the selective catalytic monoacylation of diols is possible, thus allowing efficient monodeoxygenations as exemplified in the conversion of (+)-diethyl tartrate to unnatural (+)-diethyl malate.

## Results and Discussion

**Scheme 2:**
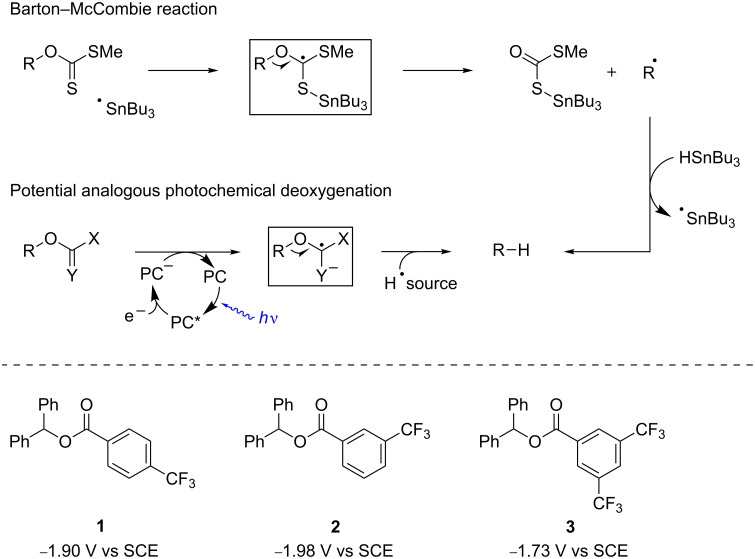
Reduction potentials of investigated derivatives **1**–**3** in DMF.

Following the lead of photochemical deoxygenations under UV light irradiation (vide supra) we envisioned carboxylic ester derivatives as substrates for initial test reactions. Benzoate esters were chosen due to the potentially very facile recovery of the benzoic acids used for activation. Through variation of the substitution pattern of benzoates we intended to shift the electrochemical reduction potentials of the substances into a region that could be accessed by common visible light photocatalysts. The substituents should be as inert as possible in order not to interfere with the photochemical reaction itself. Therefore different trifluoromethyl-substituted benzoates were prepared and subjected to cyclovoltametric measurements. 3,5-Bis(trifluoromethyl)-substituted benzoate **3** showed the most promising reduction potential of the compounds investigated (Scheme 2) and was therefore expected to be most susceptible for an initial photoredox electron transfer that we considered in analogy to the Barton–McCombie technology to be crucial to trigger deoxygenations.

**Scheme 3:**
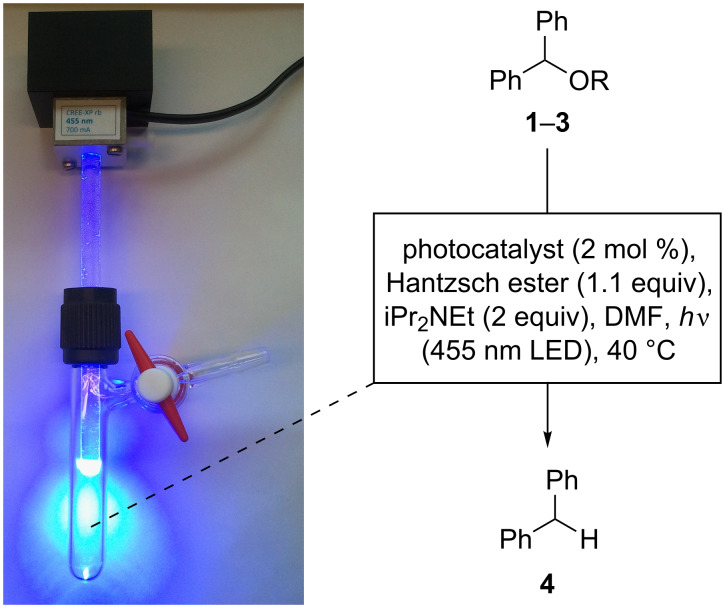
Initial reaction conditions for deoxygenation candidates **1**–**3**.

Initial deoxygenation experiments were carried out with either Ru(bpy)_3_Cl_2_·6H_2_O [bpy = 2,2'-bipyridine] or [Ir(ppy)_2_(dtb-bpy)](PF_6_) [ppy = 2-phenylpyridine; dtb-bpy = 4,4′-di-*tert*-butyl-2,2′-bipyridine] as photocatalysts, Hantzsch ester (diethyl 1,4-dihydro-2,6-dimethyl-3,5-pyridinedicarboxylate) as hydrogen donor, and iPr_2_NEt as sacrificial electron donor in DMF (Scheme 3). Light generated from a high power LED was channeled into the reaction solution in a Schlenk tube through a glass rod from above while magnetic stirring and heating was applied from below. This reaction setup is superior compared to conventional ones that are carried out in a vessel with external irradiation: a high light intensity can be achieved for irradiation and reaction temperatures can be manipulated very conveniently by heating in a conventional oil bath while carrying out the photoreaction. Also light pollution is kept to a minimum, which renders the apparatus an optimal device for the fast setup of test reactions.

In agreement with the reduction potentials, 3,5-bis(trifluoromethyl)-substituted benzoate **3** gave the best results in the deoxygenation reaction while esters **1** and **2** led to incomplete reactions after 16 h of irradiation (Table 1). The performance of [Ir(ppy)_2_(dtb-bpy)](PF_6_) was in all cases superior as compared to Ru(bpy)_3_Cl_2_·6H_2_O, possibly due to its increased reduction potential (*E*^0^ = −1.51 V vs *E*^0^ = −1.31 V) [11].

**Table 1:** Comparison of different esters and photocatalysts in deoxygenation reaction.

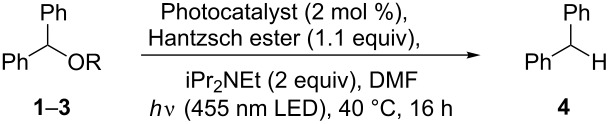			

Photocatalyst	Compound yield [%]^a^		
	**1**	**2**	**3**

Ru(bpy)_3_Cl_2_·6H_2_O	5	8	10
[Ir(ppy)_2_(dtb-bpy)](PF_6_)	18	20	85

^a^All yields determined by GC–FID with dodecane as internal standard.

Having identified a promising activation group for deoxygenation in combination with an iridium-based photocatalyst, different solvents and reaction temperatures were examined for the conversion of **3** (Table 2). Gratifyingly, toxic DMF could be replaced with more benign acetonitrile without appreciable decreasing the yield (Table 2, entry 2). The reaction also proceeded in less polar solvents (Table 2, entries 3 and 4), albeit yields were significantly lower. When the reaction was performed at ambient temperature **4** was only formed in 41% yield after 16 h of irradiation (Table 2, entry 5). Control experiments suggest that the deoxygenation reaction of 3,5-bis(trifluoromethyl)benzoate **3** is indeed a photochemically mediated process (Table 2, entries 6 and 7): when either the photocatalyst (Table 2, entry 6) or the light source (Table 2, entry 7) was omitted significantly lower yields were obtained. Leaving out Hantzsch ester (Table 2, entry 8) apparently does not impede the deoxygenation while carrying out the reaction without Hünig’s base lowers the yield (Table 2, entry 9), nevertheless, **4** was still formed to a significant extent. These results suggest that Hantzsch ester is not necessary as the hydrogen source but likewise that reductive quenching of the photocatalyst is not exclusively accomplished by Hünig’s base.

**Table 2:** Solvent/temperature dependence and control experiments of deoxygenation reaction with 3,5-bis(trifluoromethyl)benzoate **3**^a^.

Entry	Solvent, modification	Yield (%)^b^

1	DMF	85
2	MeCN	80
3	DCM	20
4	THF	22
5	MeCN, rt	41
6	MeCN, w/o photocatalyst	7
7	MeCN, w/o light source	15
8	MeCN, w/o Hantzsch ester	91
9	MeCN, w/o iPr_2_NEt	53

^a^Conditions see Table 1. ^b^All yields determined by GC–FID with dodecane as internal standard.

**Scheme 4:**
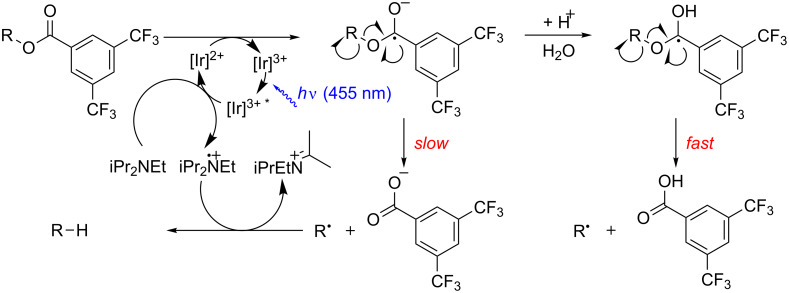
Proposed reaction mechanism with and without additional water.

**Scheme 5:**
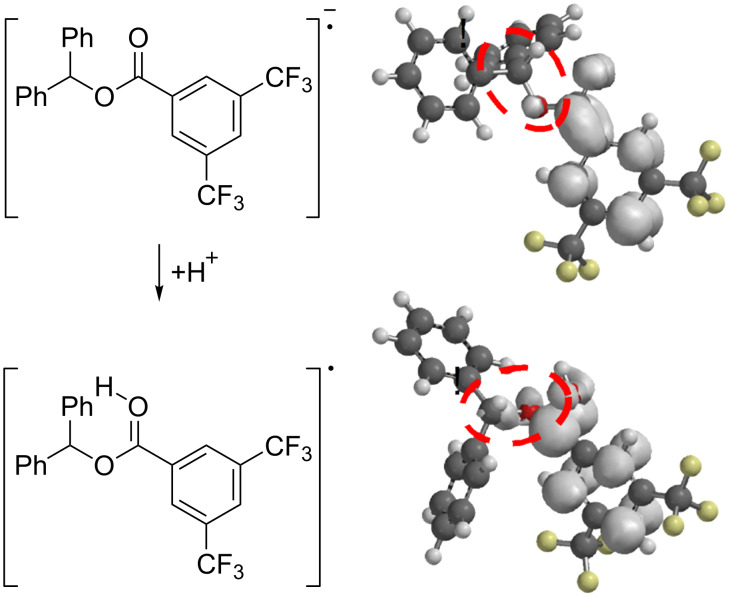
Calculated spin densities of the radical anion and its protonated species.

We assumed that the mechanism of the deoxygenation reaction involves an electron uptake of the ester moiety from the reductively quenched photocatalyst followed by mesolysis and subsequent hydrogen abstraction (Scheme 4). Quantum mechanical calculations (B3LYP/6-31G*) for benzhydryl 3,5-bis(trifluoromethyl)benzoate (**3**) revealed that the electron density of the presumed transient radical anion is mainly located at the phenyl moiety of the benzoate – and not in the desired anti-bonding σ*(C–O) (Scheme 5). Protonation of the radical anion would lead to a neutral radical species, which in the calculations reflects in a shift of electron density towards the C–O bond to be cleaved.

Consequently, a mixture of acetonitrile/water (14:1) was explored as reaction medium, resulting to our delight in dramatically reduced reaction times to achieve full conversion. Starting from bis(trifluoromethyl)benzoates, quantitative formation of the deoxygenated products could be already observed after 20 minutes of irradiation.

A simple iridium-catalyzed hydrogenation mechanism as an alternative for a photochemical pathway of the reaction could be ruled out; in the presence of 5 atm H_2_ without irradiation under otherwise unchanged reaction conditions no deoxygenation of the benzoates could be observed even after prolonged reaction times.

With the newly optimized reaction conditions at hand different benzylic alcohol derivatives were investigated (Table 3). Uniformly very good isolated yields after short reaction times were achieved in case of dibenzylic alcohol derivatives. Steric bulk (Table 3, entry 2), as well as a broad range of functional groups with different electronic properties, i.e., an electron-donating *p*-methoxy substituent (Table 3, entry 3), electron-withdrawing *p-*nitro substituent (Table 3, entry 4), ester group containing systems (Table 3, entry 5), chlorinated derivatives (Table 3, entry 6) and electron-deficient heteroaromatic systems (Table 3, entry 7) were well tolerated, giving the corresponding deoxygenated products in analytical pure form in high yields after filtration through a short plug of silica gel. Noteworthy, no reduction of reducible groups such as nitro (Table 3, entry 4) or chloro (Table 3, entry 5) was observed. Moving to monobenzyl alcohols, e.g., replacement of one aromatic group with an alkyl chain resulted in prolonged reaction times but nevertheless acceptable yields of the deoxygenated products (Table 3, entries 8 and 9). With α-carbonyl-substituted benzylic alcohol derivatives irradiation times could be reduced again and defunctionalized materials were isolated in moderate to good yields (Table 3, entries 10 and 11). Bis(trifluoromethyl)benzoic acid **5** could easily be recovered (>90%) in an acid–base extraction step.

**Table 3:** Preparative deoxygenation reactions.

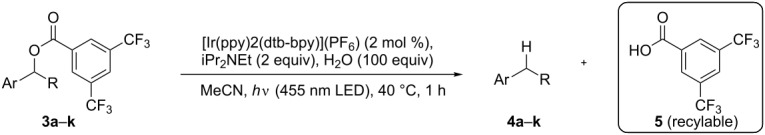			

Entry	Substrate	Product	Yield (%)^a^

1	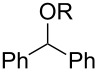 **3a**	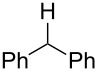 **4a**	95
2	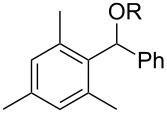 **3b**	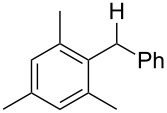 **4b**	86
3	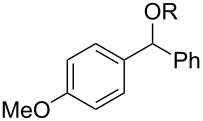 **3c**	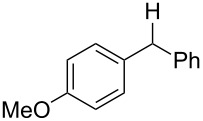 **4c**	87
4	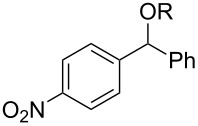 **3d**	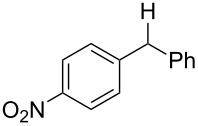 **4d**	91
5	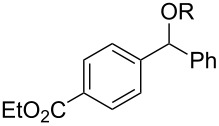 **3e**	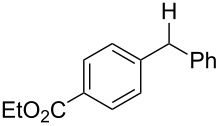 **4e**	93
6	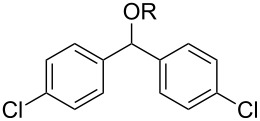 **3f**	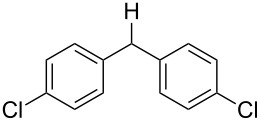 **4f**	92
7	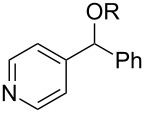 **3g**	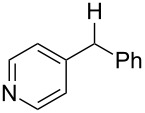 **4g**	86
8	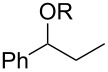 **3h**	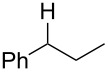 **4h**	66^b,c^
9	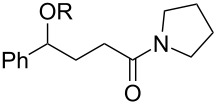 **3i**	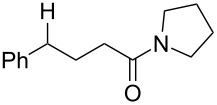 **4i**	79^c^
10	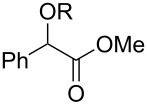 **3j**	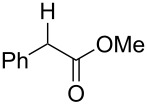 **4j**	83
11	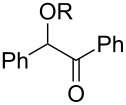 **3k**	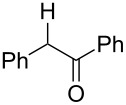 **4k**	67

^a^Isolated yields of reactions conducted at a 0.2–1.0 mmol scale. ^b^Determined by GC with dodecane as internal standard. ^c^16 h reaction time.

Also bis(trifluoromethyl)benzoates of non-benzylic α-cyanohydrin **6a** and α-hydroxycarbonyl compounds **6b–e** turned out to be amenable for the desoxygenation process (Table 4).

**Table 4:** Preparative deoxygenation reactions of non-benzylic benzoates. ^a^

Entry	Substrate	Product	Yield (%)

1	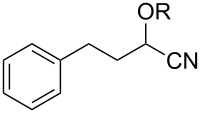 **6a**	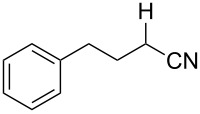 **7a**	86
2	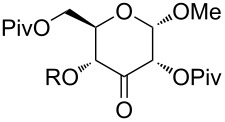 **6b**	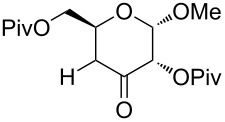 **7b**	79
3	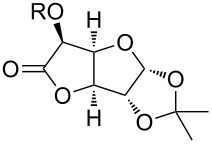 **6c**	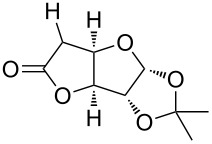 **7c**	14^b^
4	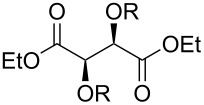 **6d**	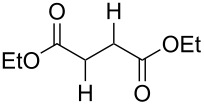 **7d**	69^c,d^
5	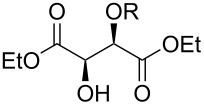 **6e**	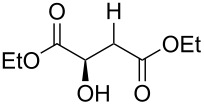 **7e**	99

^a^Conditions see Table 3. ^b^Parent compound was prone to hydrolysis under reaction conditions. ^c^16 h reaction time. ^d1^H NMR yield.

**Scheme 6:**
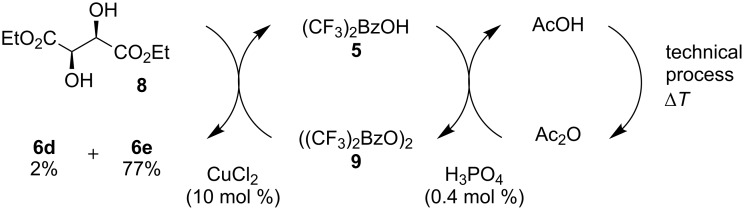
Synthesis of monobenzoate **6e**.

Especially interesting from a preparative point of view, the monodeoxygenation of diethyl tartrate to maleate could be achieved from **6e** in excellent yields (Table 4, entry 5). Notably, after screening various Lewis acids (Supporting Information File 1) it was found that **6e** could be selectively prepared by copper(II) chloride-catalyzed benzoylation of diol **8** with anhydride **9**, which in turn can be generated from its acid **5** by treatment with acetic anhydride (Scheme 6).

Since acetic anhydride is technically being produced by thermal dehydration of acetic acid [12], the overall sequence to the benzoylated starting material **6e** does not require any type of activation reagents such as thionyl chloride or DCC that are often used for ester formation, but ultimately only requires energy in form of heat. After the photochemical deoxygenation, 3,5-bis(trifluoromethyl)benzoic acid (**5**) is formed, which can be easily recovered in high yield and from which anhydride **9** can be regenerated as described above.

**Scheme 7:**
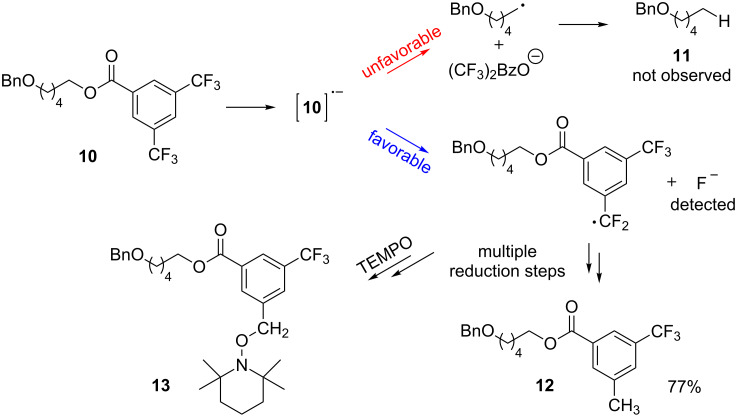
Reduction of benzoate moiety in case of non-benzylic alcohols.

Attempts to deoxygenate simple alkyl-substituted alcohols (primary, secondary and tertiary) were not successful; for example under typical conditions 3,5-bis(trifluoromethyl)benzoates such as **10** gave **12** where one trifluoromethyl group was completely reduced to a methyl group in 52% yield (Scheme 7). Apparently, electron transfer to the benzoate group is still possible however, the subsequent C–O bond cleavage does not occur, presumably due to the energetically unfavorable primary radical intermediate that would result via the desired cleavage of the C–O bond. Instead, carbon–fluorine cleavage, leading to a benzylic radical, is the preferred pathway.

To unambiguously rule out that the methyl group in **12** originates from a substitution process with acetonitrile as the methyl source, the reaction was carried out in deuterated acetonitrile. No deuterium incorporation was observed which proved that acetonitrile is not responsible for the presence of the methyl group in **12**. Performing the reaction in the presence of TEMPO (2,2,6,6-tetramethylpiperidine-1-oxyl) gave, beside reduction product **12**, adduct **13** which suggests that the methyl group originates from a sequential reduction of the C–F bonds through a radical pathway. In addition a test for fluoride with [Fe(SCN)(H_2_O)_5_]^2+^ in an evaporated aliquot of the irradiated reaction mixture was positive. Increasing the amount of Hünig’s base acting as sacrificial electron donor in the initial reduction step of **10** led to full conversion of the starting material and gave **12** as the only reaction product in 77% isolated yield.

**Scheme 8:**
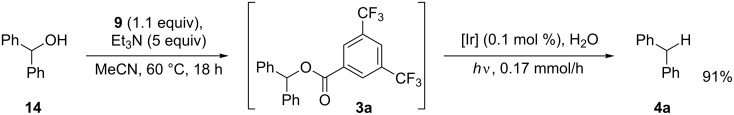
Optimized conditions for larger scale applications.

For larger scale applications it would be desirable to install the activating benzoate group in situ rather than in a foregoing reaction step. Also, considering its high price the employment of only small amounts of iridium-based catalyst and lower priced triethylamine instead of costly diisopropylethylamine would be desirable. Taking **14** as model compound it could be shown that the overall deoxygenation process can be optimized and simplified in this regard by the in situ formation of the 3,5-bis(trifluoromethyl)benzoate **3a** which in turn could then be converted to deoxygenated compound **4a** in 91% yield (Scheme 8) in a flow process using a microreactor (see Supporting Information File 1).

## Conclusion

In summary a protocol for the deoxygenation of benzylic alcohols, α-hydroxycarbonyl and α-cyanohydrin compounds under visible light photocatalysis was developed using 3,5-bis(trifluoromethyl)benzoic anhydride for alcohol activation. Since 3,5-bis(trifluoromethyl)benzoic acid can be recycled and reactivated under redox neutral conditions, and moreover, the in situ activation of alcohols with this auxiliary is possible we envision that an overall continuous process could be developed for the deoxygenation of alcohols by this protocol. That ultimately only requires heat, triethylamine as a sacrificial electron donor and visible light, forming water as the only byproduct. Therefore, we believe that despite the relatively expensive activation of alcohols as 3,5-bis(trifluoromethyl)benzoic acid esters, the deoxygenation protocol described here could become also attractive for large-scale applications.

## Experimental

General procedure for photochemical deoxygenations: A Schlenk tube was charged with photocatalyst (20 µmol, 2.0 mol %), benzoate (1.00 mmol, 1.00 equiv), sealed with a screw cap and subsequently evacuated and backfilled with N_2_ (3×). Solvent (20 mL), Hünig’s base (0.35 mL, 2.0 mmol, 2.0 equiv), and degassed water (1.8 mL, 100 mmol, 100 equiv) were added and the reaction mixture was magnetically stirred until a homogeneous solution was obtained. The reaction mixture was degassed by freeze-pump-thaw (5×) and the screw cap was replaced with a Teflon-sealed inlet for a glass rod, through which irradiation with a 455 nm high power LED took place from above while the reaction was magnetically stirred and heated in an aluminum block from below. After the reaction the mixture was diluted with 100 mL Et_2_O, washed with 50 mL 10% Na_2_CO_3_, 50 mL H_2_O, 50 mL brine, and dried over Na_2_SO_4_. After evaporation the crude mixture was purified by filtration through a short plug of flash silica gel with a mixture of petrol ether and ethyl acetate.

## Supporting Information

File 1 Experimental details, characterization data and spectra.
