# Supplementary material for: Visible light photoredox-catalyzed deoxygenation of alcohols
Source: Beilstein J Org Chem. 2014 Sep 10;10:2157–65. doi: 10.3762/bjoc.10.223 (PMC4168922; doi:10.3762/bjoc.10.223)
Supplement: File 1 — Experimental details, characterization data and spectra. [file Beilstein_J_Org_Chem-10-2157-s001.pdf]

Supporting Information

for

Visible light photoredox-catalyzed deoxygenation of alcohols

Daniel Rackl, Viktor Kais, Peter Kreitmeier and Oliver Reiser*

Address: Institute of Organic Chemistry, University of Regensburg, 93053 Regensburg, Germany

Email: Oliver Reiser* - Oliver.Reiser@uni-regensburg.de

* Corresponding author

Experimental details, characterization data and spectra

Table of Contents

1. General information S2
2. Synthesis of alcohols S3
3. General procedure for the synthesis of benzoate esters via the acid chloride (GPI) S5
4. Synthesis of the acid anhydride S12
5. General procedure for the synthesis of benzoate esters via the acid chloride (GPII) S13
6. Selective synthesis of monoacylated tartrate 6e S15
7. General procedure for photoreactions (GPIII) S18
8. In-situ acylation and deoxygenations in a microreactor S23
9. References S24
10. Spectra of new compounds S26

1. General information

All chemicals were used as received or purified according to Purification of Common Laboratory Chemicals. Glassware was dried in an oven at 110 °C or flame dried and cooled under a dry atmosphere prior to use. All reactions were performed using Schlenk techniques. The blue light irradiation in batch processes was performed using a CREE XLamp XP-E D5-15 LED (λ = 450-465 nm). In micro reactor processes 8 OSRAM OSLON Black Series LD H9GP LEDs (λ = 455±10 nm) were employed. Analytical thin layer chromatography was performed on Merck TLC aluminium sheets silica gel 60 F 254. Reactions were monitored by TLC and visualized by a short wave UV lamp and stained with a solution of potassium permanganate, p-anisaldehyde, or Seebach's stain. Column flash chromatography was performed using Merck flash silica gel 60 (0.040-0.063 mm). The melting points were measured on a Büchi SMP-20 apparatus in a silicon oil bath. Values thus obtained were not corrected. ATR-IR spectroscopy was carried out on a Biorad Excalibur FTS 3000 spectrometer, equipped with a Specac Golden Gate Diamond Single Reflection ATR-System. NMR spectra were recorded on Bruker Avance 300 and Bruker Avance 400 spectrometers. Chemical shifts for ^{1}H NMR were reported as δ, parts per million, relative to the signal of $CHCl_3$ at 7.26 ppm. Chemical shifts for ^{13}C NMR were reported as δ, parts per million, relative to the center line signal of the $CDCl_3$ triplet at 77 ppm. Coupling constants *J* are given in Hertz (Hz). The following notations indicate the multiplicity of the signals: s = singlet, brs = broad singlet, d = doublet, t = triplet, q = quartet, quint = quintet, sept = septet, and m = multiplet. Mass spectra were recorded at the Central Analytical Laboratory at the Department of Chemistry of the University of Regensburg on a Varian MAT 311A, Finnigan MAT 95, Thermoquest Finnigan TSQ 7000 or Agilent Technologies 6540 UHD Accurate-Mass Q-TOF LC/MS. Gas chromatographic analyses were performed on a Fisons Instuments gas chromatograph equipped with a capillary column (30 m × 250 µm × 0.25 µm) and a flame ionisation detector. The yields reported are referred to the isolated compounds unless otherwise stated.

2. Synthesis of alcohols

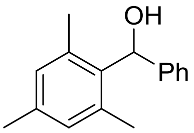

Mesityl(phenyl)methanol:[1] A 50 mL Schlenk flask equipped with a magnetic stir bar was charged with mesitaldehyde (2.22 g, 15.0 mmol, 1.00 equiv.), flushed with nitrogen, and capped with a rubber septum. THF (15 mL) was added and the mixture was cooled to 0 °C, then a solution of phenylmagnesium bromide (1.0 M in THF, 16.5 mL, 16.5 mmol, 1.10 equiv.) was added dropwise. The reaction mixture was allowed to warm to room temperature, quenched with 1 M HCl, and evaporated under reduced pressure. 50 mL DCM was added to the obtained residue, phases were separated, the aqueous layer was extracted twice with 50 mL DCM, and the combined organic layers were dried over Na_2SO_4 and evaporated under reduced pressure. The obtained oil was purified by flash column chromatography (petrol ether / EtOAc, 100:1 to 25:1) to give 2.93 g (13.0 mmol, 86%) of mesityl(phenyl)methanol as a colorless oil. ^{1}H-NMR (300 MHz, $CDCl_3$): 7.39-7.19 (m, 5 H), 6.88 (s, 2 H), 6.34 (s, 1 H), 2.30 (s, 3 H), 2.25 (s, 6 H), 2.08 (brs, 1 H).

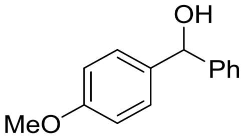

(4-Methoxyphenyl)(phenyl)methanol:[1] A 50 mL Schlenk flask equipped with a magnetic stir bar was charged with 4-methoxybenzaldehyde (2.04 g, 15.0 mmol, 1.00 equiv.), flushed with nitrogen, and capped with a rubber septum. THF (15 mL) was added and the mixture was cooled to 0 °C. A solution of phenylmagnesium bromide (1.0 M in THF, 16.5 mL, 16.5 mmol, 1.10 equiv.) was added dropwise. The reaction mixture was allowed to warm to room temperature, quenched with 1 M HCl, and evaporated under reduced pressure. 50 mL DCM was added to the obtained residue, phases were separated, the aqueous layer was extracted twice with 50 mL DCM, and the combined organic layers were dried over Na_2SO_4 and evaporated under reduced pressure. The obtained oil was purified by flash column chromatography (petrol ether / EtOAc, 100:1 to 25:1) to give 2.70 g (12.6 mmol, 84%) of (4-methoxyphenyl)(phenyl)methanol as a colorless oil. ^{1}H-NMR (300 MHz, $CDCl_3$): 7.47-7.19 (m, 7 H), 6.09-6.84 (m, 2 H), 5.82 (s, 1 H), 3.79 (s, 3 H), 1.88 (brs, 1 H).

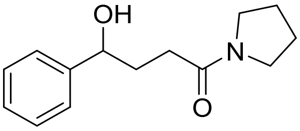

4-Hydroxy-4-phenyl-1-(pyrrolidin-1-yl)butan-1-one:[2,3] In a 100 mL round bottom flask 3-benzoylpropionic acid (4.46 g, 25.0 mmol, 1.00 equiv.) was dissolved in a mixture of NaOH (1.50 g, 37.5 mmol, 1.50 equiv.) in 15 mL H2O and 15 mL EtOH. The mixture was cooled to 0 °C and a solution of $NaBH_4$ (0.946 g, 25.0 mmol, 1.00 equiv.) in 10 mL 0.4 M NaOH was added

dropwise. The resulting reaction mixture was stirred for 1 h at room temperature and further 2 h at 60 °C upon which the reaction mixture was brought to a pH of 1 with HCl (conc.) and extracted with DCM (3 x 20 mL). The combined organic phases were washed with 20 mL 10% Na_2CO_3, 20 mL H_2O, 20 mL brine, dried over Na_2SO_4, and evaporated under reduced pressure to give 3.03 g (18.7 mmol, 75%) of 5-phenyldihydrofuran-2(3H)-one[2] as a colorless oil. ^{1}H-NMR (400 MHz, $CDCl_3$): 7.46-7.30 (m, 5 H), 5.55-5.48 (m, 1 H), 2.73-2.61 (m, 3 H), 2.27-2.12 (m, 1 H). A 50 mL Schlenk flask equipped with a magnetic stir bar was charged with 5-phenyldihydrofuran-2(3H)-one (1.05 g, 6.50 mmol, 1.00 equiv.), pyrrolidine (1.13 g, 13.0 mmol, 2.00 equiv.), Et_3N (4.54 mL, 3.34 g, 32.5 mmol, 5.00 equiv.), and equipped with a reflux condenser. The reaction mixture was refluxed for 3 d after which excess pyrrolidine and Et_3N was removed by distillation under reduced pressure to obtain 1.48 g (6.32 mmol, 97%) of 4-hydroxy-4-phenyl-1-(pyrrolidin-1-yl)butan-1-one[3] as a slightly orange solid. ^{1}H-NMR (300 MHz, $CDCl_3$): 7.43-7.19 (m, 5 H), 4.86-4.77 (m, 1 H), 4.73 (brs, 1 H), 3.48 (t, J = 7.0, 2 H), 3.35 (t, J = 6.8, 2 H), 2.43 (t, J = 6.3, 2 H), 2.20-2.02 (m, 2 H), 2.02-1.78 (m, 4 H).

3. General procedure for the synthesis of benzoate esters via the acid chloride (*GPI*)

A 50 mL Schlenk flask equipped with a magnetic stir bar was charged with an alcohol (5.00 mmol, 1.00 equiv.), 4-DMAP (31 mg, 0.25 mmol, 0.05 equiv.), Et_3N (5.0 ml, 3.5 g, 35 mmol, 7.0 equiv.), and DCM (50 mL). The mixture was cooled to 0 °C, then (trifluoromethyl)benzoyl chloride[45] (5.50 mmol, 1.10 equiv.) was added dropwise. The reaction mixture was allowed to warm to room temperature, solvent was evaporated under reduced pressure and the residue was purified by flash chromatography.

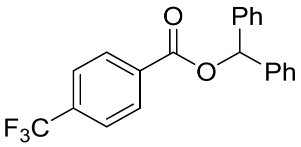

Benzhydryl 4-(trifluoromethyl)benzoate (1): Following general procedure ***GPI*** gave 1.12 g (3.13 mmol, 63%) of a white solid after purification on SiO_2 (petrol ether / EtOAc, 50:1 to 25:1). R_f (petrol ether): 0.08; mp: 92 °C; IR (neat): 3062, 1722, 1585, 1497, 1455, 1411, 1320, 1268, 1166, 1115, 1065, 1016, 966, 898, 862, 774, 742, 703, 649, 591, 457 cm^{-1}; ^{1}H-NMR (300 MHz, $CDCl_3$): 8.26 (d, J = 8.5, 2 H), 7.74 (d, J = 8.5, 2 H), 7.45-7.30 (m, 10 H), 7.15 (s, 1 H); ^{13}C-NMR (101 MHz, $CDCl_3$): 164.4, 139.9, 134.7 (q, J = 32.7), 130.2, 128.7, 128.2, 127.2, 125.5 (q, J = 3.7), 123.6 (q, J = 273), 78.1; ^{19}F-NMR (282 MHz, $CDCl_3$): -63.6; HRMS (EI) m/z calculated for $C_{21}H_{15}F_3O_2$ ($[M]^+$) 356.1024, found 356.1024.

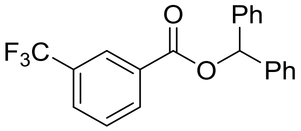

Benzhydryl 3-(trifluoromethyl)benzoate (2): Following general procedure ***GPI*** gave 1.63 g (4.58 mmol, 92%) of a white solid after purification on SiO_2 (petrol ether / EtOAc, 50:1 to 25:1). R_f (petrol ether): 0.13; mp: 73 °C; IR (neat): 3077, 3029, 1725, 1617, 1494, 1455, 1335, 1246, 1169, 1131, 1073, 985, 932, 826, 751, 695, 652, 618, 598, 570, 507, 468, 410 cm^{-1}; ^{1}H-NMR (300 MHz, $CDCl_3$): 8.39 (s, 1 H), 8.33 (d, J = 7.9, 1 H), 7.84 (d, J = 7.8, 1 H), 7.61 (t, J = 7.8, 1 H), 7.48-7.41 (m, 4 H), 7.41-7.28 (m, 6 H), 7.16 (s, 1 H); ^{13}C-NMR (101 MHz, $CDCl_3$): 164.4, 139.8, 133.0, 131.2 (q, J = 32.8), 131.1, 129.7 (q, J = 3.7), 129.2, 128.7, 128.2, 127.2, 126.7 (q, J = 3.9), 123.7 (q, J = 273), 78.1; ^{19}F-NMR (282 MHz, $CDCl_3$): -63.3; HRMS (APCI) m/z calculated for $C_{21}H_{19}F_3NO_2$ ($[M+NH_4]^+$) 374.1362, found 374.1362.

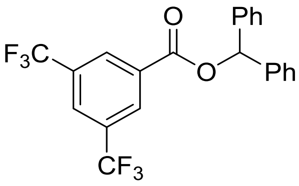

Benzhydryl 3,5-bis(trifluoromethyl)benzoate (3): Following general procedure ***GPI*** gave 1.60 g (3.76 mmol, 75%) of a white solid after purification on SiO_2 (petrol ether / EtOAc, 100:1 to 50:1). R_f (petrol ether): 0.18; mp: 71 °C; IR (neat): 3110, 3063, 3033, 1732, 1621, 1496, 1456, 1388, 1248, 1173, 1120, 989, 912, 846, 768, 750, 696, 623, 603, 562, 475, 430 cm^{-1}; ^{1}H-NMR (300 MHz, $CDCl_3$): 8.56 (s, 2 H), 8.09 (s, 1 H), 7.45-7.32 (m, 10 H), 7.19 (s, 1 H); ^{13}C-NMR (101 MHz, $CDCl_3$): 163.1, 139.3, 132.4, 132.3 (q, J = 34.3), 129.9 (m), 128.8, 128.4, 127.2, 126.6 (sept, J = 3.7), 122.9 (q, J = 273), 78.9; ^{19}F-NMR (282 MHz, $CDCl_3$): -63.4; HRMS (EI) m/z calculated for $C_{22}H_{14}F_6O_2$ ($[M]^+$) 424.0898, found 424.0899.

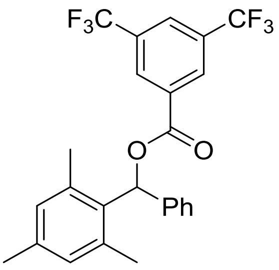

Mesityl(phenyl)methyl 3,5-bis(trifluoromethyl)benzoate (3b): Following general procedure ***GPI*** using mesityl(phenyl)methanol (438 mg, 1.94 mmol, 1.00 equiv.), 3,5-bis(trifluoromethyl)benzoyl chloride (370 µL, 563 mg, 2.04 mmol, 1.05 equiv.), 4-DMAP (12 mg, 0.10 mmol, 0.05 equiv.), Et_3N (2.0 mL, 1.4 g, 14 mmol, 7.0 equiv.), and DCM (20 mL) gave 900 mg (1.93 mmol, 99%) of a white solid after purification on SiO_2 (petrol ether / EtOAc, 100:1 to 30:1). R_f (petrol ether): 0.41; mp: 75 °C, IR (neat): 2969, 2919, 2869, 1721, 1614, 1495, 1448, 1390, 1354, 108, 1254, 1190 1129, 1036, 957, 951, 911, 854, 807, 760, 725, 695, 622, 599, 531, 497, 439 cm^{-1}; ^{1}H-NMR (300 MHz, $CDCl_3$): 8.57 (s, 2 H), 8.10 (s, 1 H), 7.67 (s, 1 H), 7.42-7.29 (m, 3 H), 7.22-7.14 (m, 2H), 6.91 (m, 2 H); 2.38 (s, 6 H), 2.30 (s, 3 H). ^{13}C-NMR (75 MHz, $CDCl_3$): 163.4, 138.8, 138.4, 137.7, 132.6, 132.4, 132.1, 132.0, 130.0, 129.9 (m), 128.6, 127.6, 126.5 (m), 125.7, 124.6, 74.8, 21.0, 20.6; ^{19}F-NMR (282 MHz, $CDCl_3$): -63.5; HRMS (EI) m/z calculated for $C_{25}H_{20}F_6O_2$ ($[M]^+$) 466.1367, found 466.1363.

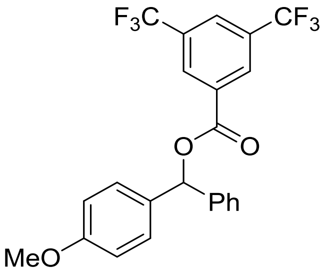

(4-Methoxyphenyl)(phenyl)methyl 3,5-bis(trifluoromethyl)-benzoate (3c): Following general procedure ***GPI*** using (4-methoxyphenyl)(phenyl)methanol (429 mg, 2.00 mmol, 1.00 equiv.), 3,5-bis(trifluoromethyl)benzoyl chloride (381 µL, 581 mg, 2.10 mmol, 1.05 equiv.), 4-DMAP (12 mg, 0.10 mmol, 0.05 equiv.), Et_3N (2.0 mL, 1.4 g, 14 mmol, 7.0 equiv.), and DCM (20 mL) gave 732 mg (1.61 mmol, 81%) of a colorless oil after purification on SiO_2 (petrol ether / EtOAc, 100:1 to 20:1). R_f (petrol ether): 0.13; IR (neat): 2840, 2362, 1730, 1613, 1514, 1457, 1388, 1278, 1242, 1175, 1136, 1035, 912, 821, 767, 700, 632, 544, 501 cm^{-1}; ^{1}H-NMR (300 MHz, $CDCl_3$): 8.55 (s, 2 H), 8.08 (s, 1 H), 7.46-7.29 (m, 7 H), 7.16 (s, 1 H), 6.96-6.88 (m, 2

H), 3.81 (s, 3 H); ^{13}C-NMR (75 MHz, $CDCl_3$): 163.1, 159.7, 139.5, 132.5, 132.3 (q, *J* = 34.0), 131.4, 129.8 (m), 129.0, 128.7, 128.7, 128.2, 127.0, 126.6 (m), 122.9 (q, *J* = 273), 114.1, 78.6, 55.3; ^{19}F-NMR (282 MHz, $CDCl_3$): -63.4; HRMS (EI) m/z calculated for $C_{23}H_{16}F_6O_3$ ($[M]^+$) 454.1004, found 454.1003.

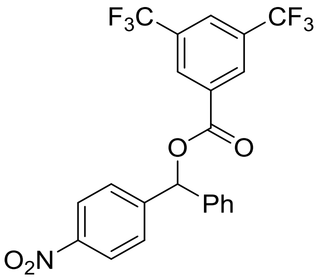

(4-Nitrophenyl)(phenyl)methyl 3,5-bis(trifluoromethyl)-benzoate (3d): Following general procedure ***GPI*** using (4-nitrophenyl)(phenyl)methanol[6] (252 mg, 1.10 mmol, 1.00 equiv.), 3,5-bis(trifluoromethyl)benzoyl chloride (218 µL, 333 mg, 1.21 mmol, 1.10 equiv.), 4-DMAP (6 mg, 0.05 mmol, 0.05 equiv.), Et_3N (1.1 mL, 0.80 g, 7.9 mmol, 7.0 equiv.), and DCM (11 mL) gave 528 mg (1.10 mmol, 100%) of a colorless oil after purification on SiO_2 (petrol ether / EtOAc, 30:1). R_f (petrol ether / EtOAc, 50:1): 0.19; IR (neat): 3085, 1732, 1609, 1523, 1348, 1227, 1236, 1175, 1127, 974, 912, 845, 765, 742, 697, 613, 574, 516, 461, 440 cm^{-1}; ^{1}H-NMR (300 MHz, $CDCl_3$): 8.54 (s, 2 H), 8.30-8.21 (m, 2 H), 8.11 (s, 1 H), 7.65-7.55 (m, 2 H), 7.48-7.35 (m, 5 H) 7.22 (s, 1 H); ^{13}C-NMR (101 MHz, $CDCl_3$): 162.9, 147.8, 146.3, 137.9, 132.6 (q, *J* = 34.0), 131.8, 129.9 (m), 129.2, 127.9, 127.4, 127.0 (m), 124.1, 122.8 (q, *J* = 273), 77.8; ^{19}F-NMR (282 MHz, $CDCl_3$): -63.4; HRMS (EI) m/z calculated for $C_{22}H_{13}F_6NO_4$ ($[M+H]^+$) 470.0822, found 470.0816.

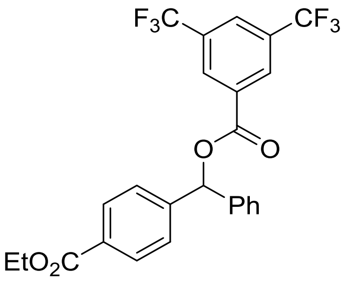

(4-(Ethoxycarbonyl)phenyl)(phenyl)methyl 3,5-bis(trifluoro-methyl)benzoate (3e): Following general procedure ***GPI*** using ethyl 4-(hydroxy(phenyl)methyl)benzoate[7] (513 mg, 2.00 mmol, 1.00 equiv.), 3,5-bis(trifluoromethyl)benzoyl chloride (381 µL, 581 mg, 2.10 mmol, 1.05 equiv.), 4-DMAP (12 mg, 0.10 mmol, 0.05 equiv.), Et_3N (2.0 mL, 1.4 g, 14 mmol, 7.0 equiv.), and DCM (20 mL) gave 954 mg (1.92 mmol, 96%) of a white solid after purification on SiO_2 (petrol ether / EtOAc, 20:1). R_f (petrol ether / EtOAc, 6:1): 0.56; mp: 98 °C; IR (neat): 2984, 1720, 1708, 1615, 1459, 1367, 1273, 1254, 1181, 1130, 1107, 1022, 979, 912, 874, 846, 754, 704, 677, 617, 570, 516, 484, 436 cm^{-1}; ^{1}H-NMR (300 MHz, $CDCl_3$): 8.55 (s, 2 H), 8.10 (s, 1 H), 8.09-8.03 (m, 2 H), 7.53-7.48 (m, 2 H), 7.45-7.31 (m, 5 H), 7.20 (s, 1 H), 4.38 (q, *J* = 7.2, 2 H), 1.39 (t, *J* =7.2, 3 H); ^{13}C-NMR (101 MHz, $CDCl_3$): 166.1, 163.0, 144.0, 138.6, 132.4 (q, *J* = 34.0), 132.2, 130.5, 130.0, 129.8 (m), 128.9, 128.8, 127.4, 126.9, 126.7 (m), 122.8 (q, *J* = 273), 78.4, 61.1, 14.3; ^{19}F-NMR (282 MHz, $CDCl_3$): -63.5; HRMS (EI) m/z calculated for $C_{25}H_{18}F_6O_4$ ($[M]^+$) 496.1109, found 496.1109.

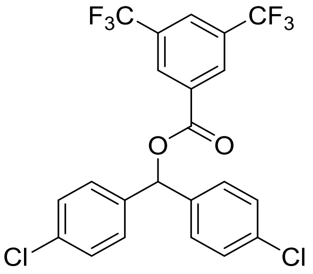

Bis(4-chlorophenyl)methyl 3,5-bis(trifluoromethyl)benzoate (3f): Following general procedure ***GPI*** using bis(4-chlorophenyl)methanol[8] (253 mg, 1.00 mmol, 1.00 equiv.), 3,5-bis(trifluoromethyl)benzoyl chloride (200 µL, 303 mg, 1.10 mmol, 1.10 equiv.), 4-DMAP (6 mg, 0.05 mmol, 0.05 equiv.), Et_3N (1.0 mL, 0.7 g, 7.0 mmol, 7.0 equiv.), and DCM (10 mL) gave 490 mg (0.99 mmol, 99%) of a white solid after purification on SiO_2 (petrol ether / EtOAc, 20:1). R_f (petrol ether / EtOAc, 6:1): 0.78; mp: 99 °C; IR (neat): 3101, 1729, 1625, 1493, 1346, 1271, 1239, 1185, 1136, 1125, 1091, 994, 912, 830, 799, 771, 705, 581, 530, 498, 443 cm^{-1}; ^{1}H-NMR (300 MHz, $CDCl_3$): 8.51 (s, 2 H), 8.10 (s, 1 H), 7.42-7.29 (m, 8 H), 7.10 (s, 1 H); ^{13}C-NMR (75 MHz, $CDCl_3$): 162.9, 137.3, 134.7, 132.4 (q, J = 34.0), 132.0, 129.8 (m), 129.1, 128.6, 126.9 (m), 122.8 (q, J = 274), 77.2. ^{19}F-NMR (282 MHz, $CDCl_3$): -63.4; HRMS (EI) m/z calculated for $C_{22}H_{12}Cl_2F_6O_2$ ($[M]^+$) 492.0119, found 492.0119.

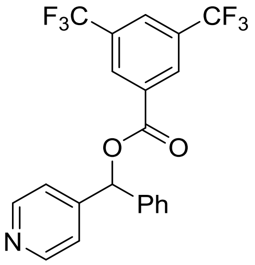

Phenyl(pyridin-4-yl)methyl 3,5-bis(trifluoromethyl)benzoate (3g): Following general procedure ***GPI*** using phenyl(pyridin-4-yl)methanol[9] (370 mg, 2.00 mmol, 1.00 equiv.), 3,5-bis(trifluoromethyl)benzoyl chloride (381 µL, 581 mg, 2.10 mmol, 1.05 equiv.), 4-DMAP (12 mg, 0.10 mmol, 0.05 equiv.), Et_3N (2.0 mL, 1.4 g, 14 mmol, 7.0 equiv.), and DCM (20 mL) gave 561 mg (1.32 mmol, 66%) of a white solid after purification on SiO_2 (petrol ether / EtOAc, 4:1). R_f (petrol ether / EtOAc, 4:1): 0.26; mp: 73 °C; IR (neat): 3034, 1730, 1603, 1496, 1458, 1413, 1359, 1273, 1244, 1181, 1124, 999, 914, 847, 792, 771, 756, 699, 654, 620, 597, 478, 438 cm^{-1}; ^{1}H-NMR (300 MHz, $CDCl_3$): 8.64 (d, J = 5.6, 2 H), 8.55 (s, 2 H), 8.11 (s, 1 H), 7.46-7.36 (m, 5 H), 7.35-7.29 (m, 2 H), 7.12 (s, 1 H); ^{13}C-NMR (75 MHz, $CDCl_3$): 162.9, 150.1, 148.2, 137.7, 132.5 (q, J = 33.8), 131.8, 129.8 (m), 129.2, 129.1, 128.2, 127.6, 126.9 (sept, J = 3.7), 122.8 (q, J = 274), 121.5, 77.4; ^{19}F-NMR (282 MHz, $CDCl_3$): -63.5; HRMS (EI) m/z calculated for $C_{21}H_{13}F_6NO_2$ ($[M]^+$) 425.0850, found 425.0847.

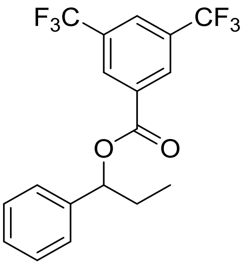

1-Phenylpropyl 3,5-bis(trifluoromethyl)benzoate (3h): Following general procedure ***GPI*** using 1-phenylpropan-1-ol (545 mg, 4.00 mmol, 1.00 equiv.), 3,5-bis(trifluoromethyl)benzoyl chloride (760 µL, 1.16 g, 4.20 mmol, 1.05 equiv.), 4-DMAP (24 mg, 0.20 mmol, 0.05 equiv.), Et_3N

(4.0 mL, 3.1 g, 28 mmol, 7.0 equiv.), and DCM (40 mL) gave 1.23 g (3.27 mmol, 81.7%) of a colorless oil after purification on SiO_2 (petrol ether / EtOAc, 50:1). R_f (petrol ether / EtOAc, 50:1): 0.62; IR (neat): 2974, 2882, 1729, 1624, 1457, 1277, 1244, 1175, 1129, 912, 845, 761, 698, 626, 546, 485, 437 cm^{-1}; ^{1}H-NMR (300 MHz, $CDCl_3$): 8.50 (s, 2 H), 8.06 (s, 1 H), 7.46-7.28 (m, 5 H), 5.95 (t, J = 7.0, 1 H), 2.24-1.91 (m, 2 H), 0.98 (t, J = 7.4, 3 H); ^{13}C-NMR (101 MHz, $CDCl_3$): 163.3, 139.6, 132.7, 132.2 (q, J = 34.0), 129.7 (m), 128.7, 128.4, 126.7, 126.3 (m), 122.9 (q, J = 274), 79.7, 29.2, 10.1; ^{19}F-NMR (376 MHz, $CDCl_3$): -62.9; HRMS (EI) m/z calculated for $C_{18}H_{14}F_6O_2$ ($[M+H]^+$) 376.0898, found 376.0896.

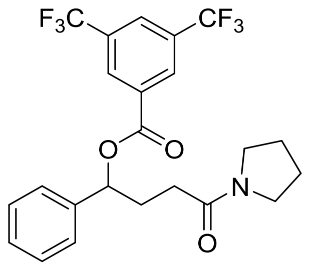

4-Oxo-1-phenyl-4-(pyrrolidin-1-yl)butyl 3,5-bis(trifluoromethyl)-benzoate (3i): Following general procedure ***GPI*** using 4-hydroxy-4-phenyl-1-(pyrrolidin-1-yl)butan-1-one (467 mg, 2.00 mmol, 1.00 equiv.), 3,5-bis(trifluoromethyl)benzoyl chloride (397 µL, 606 mg, 2.20 mmol, 1.10 equiv.), 4-DMAP (12 mg, 0.10 mmol, 0.05 equiv.), Et_3N (2.0 mL, 1.6 g, 14 mmol, 7.0 equiv.), and DCM (20 mL) gave 899 mg (1.90 mmol, 95%) of a white solid after purification on SiO_2 (petrol ether / EtOAc, 2:1). R_f (petrol ether / EtOAc, 2:1): 0.27; mp: 99 °C; IR (neat): 2974, 2878, 1729, 1642, 1444, 1255, 1168, 1035, 1005, 911, 844, 767, 698, 583, 533, 439 cm^{-1}; ^{1}H-NMR (300 MHz, $CDCl_3$): 8.49 (s, 2 H), 8.06 (s, 1 H), 7.49-7.27 (m, 5 H), 6.17-6.04 (m, 1 H), 3.43 (t, J = 7.0, 2 H), 3.29 (t, J = 6.6, 2 H), 2.57-2.20 (m, 4 H), 1.97-1.73 (m, 4 H); ^{13}C-NMR (101 MHz, $CDCl_3$): 169.9, 163.2, 139.3, 132.6, 132.2 (q, J = 34.0), 129.8 (m), 128.7, 128.5, 126.6, 126.4 (sept, J = 3.7), 122.9 (q, J = 274), 77.9, 46.5, 45.7, 31.1, 30.5, 26.0, 24.3; ^{19}F-NMR (282 MHz, $CDCl_3$): -63.4; HRMS (EI) m/z calculated for $C_{23}H_{22}F_6NO_3$ ($[M+H]^+$) 474.1498, found 474.1494.

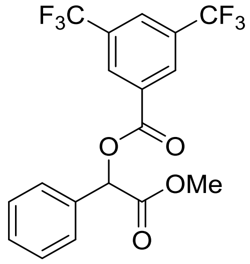

2-Methoxy-2-oxo-1-phenylethyl 3,5-bis(trifluoromethyl)benzoate (3j): Following general procedure ***GPI*** using methyl 2-hydroxy-2-phenylacetate (332 mg, 2.00 mmol, 1.00 equiv.), 3,5-bis(trifluoromethyl)benzoyl chloride (397 µL, 606 mg, 2.20 mmol, 1.10 equiv.), 4-DMAP (12 mg, 0.10 mmol, 0.05 equiv.), Et_3N (2.0 mL, 1.6 g, 14 mmol, 7.0 equiv.), and DCM (20 mL) gave 800 mg (1.97 mmol, 98%) of a colorless oil after purification on SiO_2 (petrol ether / EtOAc, 10:1). R_f (petrol ether / EtOAc, 6:1): 0.60; IR (neat): 2959, 2361, 1735, 1760, 1624, 1350, 1277, 1240, 1216, 1173, 1130, 1031, 968, 913, 845, 767, 697, 617, 542, 498, 463 cm^{-1}; ^{1}H-NMR (300 MHz, $CDCl_3$): 8.54 (s, 2 H), 8.10 (s, 1 H), 7.61-7.53 (m, 2 H), 7.52-7.42 (m, 3 H), 6.21 (s, 1 H), 3.78 (s, 3 H); ^{13}C-NMR (75 MHz, $CDCl_3$): 168.7, 163.4, 133.1, 132.3 (q, J = 34.0), 131.4, 130.1 (m),

129.8, 129.1, 127.9, 126.9 (m), 122.8 (q, J = 274), 75.8, 53.0; ^{19}F-NMR (282 MHz, $CDCl_3$): -63.4; HRMS (EI) m/z calculated for $C_{25}H_{18}F_6O_4$ ($[M+NH_4]^+$) 424.0978, found 424.0970.

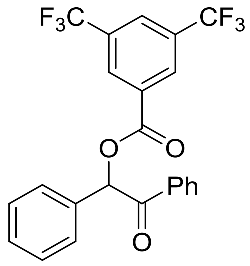

2-Oxo-1,2-diphenylethyl 3,5-bis(trifluoromethyl)benzoate (3k): Following general procedure ***GPI*** using 2-hydroxy-1,2-diphenylethanone (414 mg, 2.00 mmol, 1.00 equiv.), 3,5-bis(trifluoromethyl)benzoyl chloride (397 µL, 606 mg, 2.20 mmol, 1.10 equiv.), 4-DMAP (12 mg, 0.10 mmol, 0.05 equiv.), Et_3N (2.0 mL, 1.6 g, 14 mmol, 7.0 equiv.), and DCM (20 mL) gave 850 mg (1.88 mmol, 98%) of a white solid after purification on SiO_2 (petrol ether / EtOAc, 25:1). R_f (petrol ether / EtOAc, 6:1): 0.57; ^{1}H-NMR (300 MHz, $CDCl_3$): 8.58 (s, 2 H), 8.11 (s, 1 H), 8.01 (d, J = 7.4, 2 H), 7.72-7.50 (m, 3 H), 7.50-7.32 (m, 5 H), 7.19 (s, 1 H); ^{13}C-NMR (101 MHz, $CDCl_3$): 192.7, 163.5, 134.3, 133.8, 132.9, 132.3 (q, J = 34.0), 131.7, 130.1 (m), 129.9, 129.5, 129.0, 128.9, 128.8, 126.8 (m), 122.8 (q, J = 274), 79.2; ^{19}F-NMR (282 MHz, $CDCl_3$): -63.4; HRMS (EI) m/z calculated for $C_{25}H_{18}F_6O_4$ ($[M+NH_4]^+$) 470.1185, found 470.1189.

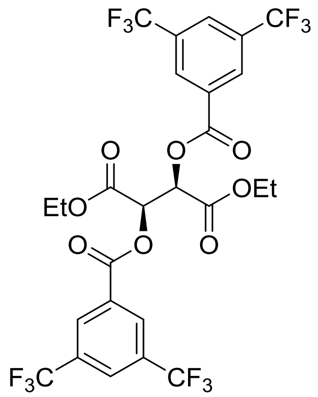

(2*R*,3*R*)-Diethyl 2,3-bis(3,5-bis(trifluoromethyl)benzoyloxy)-succinate (6d): Following general procedure ***GPI*** gave 1.54 g (2.24 mmol, 45% (81% based on 3,5-bis(trifluoromethyl)benzoyl chloride)) of a white solid after purification on SiO_2 (petrol ether / EtOAc, 6:1). R_f (petrol ether / EtOAc, 4:1): 0.61; mp: 88 - 90 °C; IR (neat): 2991, 2970, 1760, 1742, 1626, 1459, 1375, 1280, 1219, 1173, 1126, 1057, 938, 905, 847, 802, 767, 695, 680, 525, 492, 436 cm^{-1}; ^{1}H-NMR (300 MHz, $CDCl_3$): 8.25 (d, J = 1.4, 4 H), 8.14 (s, 2 H), 6.10 (s, 2 H), 4.28 (qd, J = 7.2, 1.2, 4 H), 1.25 (t, J = 7.1, 6 H); ^{13}C-NMR (75 MHz, $CDCl_3$): 165.1, 162.6, 132.7 (q, J = 34.6), 130.7, 130.0, 127.3, 122.7 (q, J = 274), 72.1, 63.0, 14.0; ^{19}F-NMR (282 MHz, $CDCl_3$): -63.8; HRMS (EI) m/z calculated for $C_{26}H_{19}F_{12}O_8$ ($[M+H]^+$) 687.0883, found 687.0882.

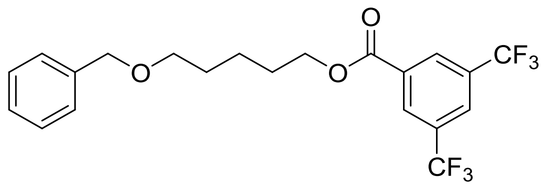

5-(Benzyloxy)pentyl 3,5-bis(trifluoromethyl)-benzoate (10): Following general procedure ***GPI*** using 5-(benzyloxy)pentan-1-ol[10] (583 mg, 3.00 mmol, 1.00 equiv.), 3,5-

bis(trifluoromethyl)benzoyl chloride (598 µL, 912 mg, 3.30 mmol, 1.10 equiv.), 4-DMAP (18 mg, 0.15 mmol, 0.05 equiv.), Et_3N (3.0 mL, 2.4 g, 21 mmol, 7.0 equiv.), and DCM (30 mL) gave 1.26 g (2.91 mmol, 97%) of a colorless oil after purification on SiO_2 (petrol ether / EtOAc, 10:1). R_f (petrol ether / EtOAc, 10:1): 0.51; IR (neat): 2941, 2866, 1729, 1621, 1455, 1377, 1277, 1247, 1175, 1134, 965, 912, 844, 770, 734, 698, 614 cm^{-1}; ^{1}H-NMR (400 MHz, $CDCl_3$): 8.48 (s, 2 H), 8.06 (s, 1 H), 7.38-7.23 (m, 5 H), 4.51 (s, 2 H), 4.40 (t, *J* = 6.7, 2 H), 3.51 (t, *J* = 6.3, 2 H), 1.89-1.78 (m, 2 H), 1.77-1.65 (m, 2 H), 1.60-1.49 (m, 2 H); ^{13}C-NMR (101 MHz, $CDCl_3$): 164.0, 132.6, 132.1 (q, *J* = 34.0), 129.7 (m), 128.4, 127.6, 127.6, 126.3 (m), 122.9 (q, J = 274), 73.0, 70.0, 29.4, 28.5, 22.8. ^{19}F-NMR (282 MHz, $CDCl_3$): -63.4; HRMS (EI) m/z calculated for $C_{21}H_{21}F_6O_3$ ($[M+H]^+$) 435.1389, found 435.1386.

4. Synthesis of 3,5-bis(trifluoromethyl)benzoic anhydride

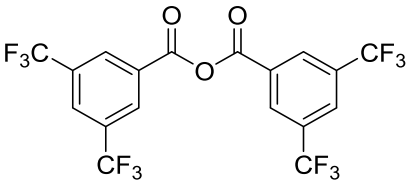

3,5-Bis(trifluoromethyl)benzoic anhydride (9): A 50 mL three-neck round-bottom flask was equipped with a gas inlet, a dropping funnel, and a 20 cm Vigreux column with a Claisen bridge and a 10 mL round-bottom flask. The flask was charged with 3,5-bis(trifluoromethyl)benzoic acid (12.0 g, 46.5 mmol, 1.00 equiv.) and phosphoric acid (20 mg, 0.20 mmol, 0.43 mol%) and the dropping funnel was charged with acetic anhydride (8.8 mL, 9.5 g, 93 mmol, 2.0 equiv.). The flask was heated to 150 °C in an oil bath and about 6.5 mL of acetic anhydride was added via the dropping funnel. The mixture was slowly heated to 190 °C till no more acetic anhydride distilled. About half of the remaining acetic anhydride was added to the reaction mixture via the dropping funnel and the mixture was stirred till no more acetic anhydride distilled. The remainder of acetic anhydride was added and again till no more acetic anhydride distilled. Vaccum (20 mbar) was applied and distillation was continued at 190 °C till no more distillate could be collected. The reaction mixture was allowed to cool to 100 °C after which the Vigreux column and the Claisen bridge were replaced with a distillation arch. The crude product was subsequently distilled at 170 °C / 1 mbar into a 50 mL Schlenk flask. Pure product was obtained after recrystallization from toluene / petrol ether as white crystals (9.07 g, 18.2 mmol, 78%). mp: 104 - 105 °C; IR (neat): 3103, 1801, 1749, 1622, 1384, 1286, 1172, 1126, 1052, 100, 915, 888, 843, 753, 726, 698, 681, 642, 613 cm^{-1}; ^{1}H-NMR (300 MHz, $CDCl_3$): 8.58 (s, 4 H), 8.22 (s, 2 H); ^{13}C-NMR (400 MHz, $CDCl_3$): 159.2, 133.1 (q, J = 34), 130.5 (m), 130.4, 128.3 (m), 122.5 (q, J = 273); ^{19}F-NMR (282 MHz, $CDCl_3$): -63.6.

5. General procedure for the synthesis of benzoate esters via the acid anhydride (*GPII*)

A 50 mL Schlenk flask equipped with a magnetic stir bar was charged with an alcohol (2.00 mmol, 1.00 equiv.), 3,5-bis(trifluoromethyl)benzoic anhydride (**9**, 1.20 g, 2.40 mmol, 1.20 equiv.), Et_3N (0.56 ml, 0.41 g, 4.0 mmol, 2.0 equiv.), and DCM (20 mL). The mixture was stirred at room temperature for 2 h. The solvent was evaporated under reduced pressure and the residue was redissolved in 20 mL EtOAc and extracted with 20 mL Na_2CO_3 (aq, 10%) and 20 mL H_2O. The united aqueous phases were acidified with 6 M HCl to give 3,5-bis(trifluoromethyl)benzoic acid (**5**) as a white solid which was filtered and dried *in vacuo*. The organic phase was evaporated and purified by flash chromatography.

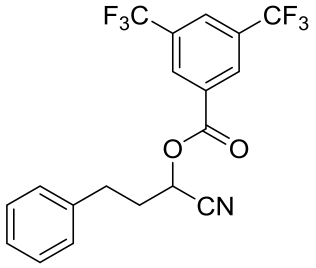

1-Cyano-3-phenylpropyl 3,5-bis(trifluoromethyl)benzoate (6a): Following general procedure ***GPII*** using 2-hydroxy-4-phenylbutanenitrile[11] gave 0.683 g (1.70 mmol, 85%) of a colorless oil after purification on SiO_2 (petrol ether / EtOAc, 10:1). R_f (petrol ether / EtOAc, 4:1): 0.82; IR (neat): 2928, 2855, 1743, 1621, 1496, 1456, 1387, 1278, 1235, 1177, 1133, 1030, 993, 911, 845, 761, 732, 697, 681, 650, 486 cm^{-1}; ^1H-NMR (300 MHz, $CDCl_3$): 8.40 (s, 2 H), 8.13 (s, 1 H), 7.35-7.15 (m, 5 H), 5.59 (t, J = 6.8, 1 H), 3.05-2.85 (m, 2 H), 2.47 (q, J = 7.1, 2 H); ^{13}C-NMR (75 MHz, $CDCl_3$): 162.3, 138.6, 132.6 (q, *J* = 34.0), 130.3, 130.0 (m), 128.9, 128.3, 127.4 (m), 126.9, 122.6 (q, J = 274), 116.1, 66.3, 33.7, 31.0; ^{19}F-NMR (282 MHz, $CDCl_3$): -63.5; HRMS (ESI) m/z calculated for $C_{19}H_{14}F_6NO_2$ ($[M+H]^+$) 402.0923, found 402.0929.

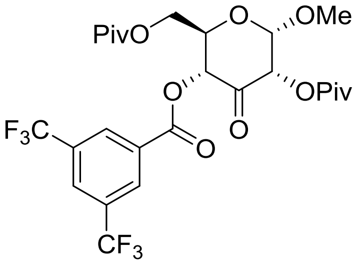

(2*R*,3*R*,5*S*,6*S*)-6-Methoxy-4-oxo-5-(pivaloyloxy)-2-(pivaloyloxymethyl)tetrahydro-2*H*-pyran-3-yl 3,5-bis(trifluoromethyl)benzoate (6b): Following general procedure ***GPII*** using (2S,3S,5R,6R)-5-hydroxy-2-methoxy-4-oxo-6-(pivaloyloxymethyl)tetrahydro-2H-pyran-3-yl pivalate[12] (258 mg, 0.716 mmol, 1.00 equiv.), 3,5-bis(trifluoromethyl)benzoic anhydride (**9**, 392 mg, 0.787 mmol, 1.10 equiv.), $^iPr_2NEt$ (249 µL, 185 mg, 1.43 mmol, 2.0 equiv.), and DCM (7 mL) gave 207 mg (0.803 mmol, 76% based on benzoate that was employed and not incorporated in the product) 3,5-bis(trifluoromethyl)benzoic acid (**5**) as a white powder and 309 mg (0.514 mmol, 71.8%)

(2R,3R,5S,6S)-6-methoxy-4-oxo-5-(pivaloyloxy)-2-(pivaloyloxy-methyl)tetrahydro-2H-pyran-3-yl 3,5-bis(trifluoromethyl)benzoate (**6b**) as a white solid after purification on SiO_2 (petrol ether / EtOAc, 6:1 to 3:1). R_f (petrol ether / EtOAc, 4:1): 0.48; mp: 46 °C; IR (neat): 2997, 1734, 1624, 1370, 1278, 1246, 1127, 1055, 1036, 911, 846, 762, 701, 682 cm^{-1}; ^{1}H-NMR (300 MHz, $CDCl_3$): 8.49 (s, 2 H), 8.10 (s, 1 H), 5.62 (dd, *J* = 10.0, 1.0, 1 H), 5.45 (dd, *J* = 4.2, 1.0, 1 H), 4.24 (d, *J* = 4.2, 1 H), 4.47-4.40 (m, 2 H), 4.39-4.32 (m, 1 H), 3.52 (s, 3 H), 1.27 (s, 9 H), 1.24 (s, 9 H); ^{13}C-NMR (75 MHz, $CDCl_3$): 192.8, 177.9, 177.0, 162.2, 132.4 (q, *J* = 34.0), 130.9, 130.1 (d, *J* = 3.0), 127.0 (m), 122.6 (q, *J* = 274), 100.0, 74.4, 73.3, 69.6, 62.2, 56.0, 39.0, 38.9, 27.1, 27.0; ^{19}F-NMR (282 MHz, $CDCl_3$): -63.5; HRMS (ESI) m/z calculated for $C_{26}H_{30}F_6NaO_9$ ($[M+Na]^+$) 623.1686, found 623.1686.

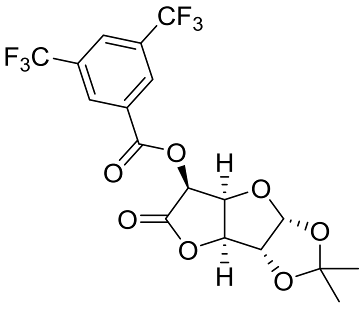

1,2-*O*-Isopropylidene-5-*O*-(3,5-bis(trifluoromethyl)benzoyl)-α-D-*xylo*-hexofuranurono-6,3-lactone (6c): Following general procedure ***GPII*** using 1,2-*O*-isopropylidene-5-*O*-(3,5-bis(trifluoromethyl)benzoyl)-α-D-xylo-hexofuranurono-6,3-lactone[13] (649 mg, 3.00 mmol, 1.00 equiv.), 3,5-bis(trifluoromethyl)benzoic anhydride (**9**, 1.50 g, 3.00 mmol, 1.00 equiv.), ^{i}Pr$_2$NEt (1.05 mL, 775 mg, 6.00 mmol, 2.0 equiv.), and DCM (30 mL) gave 432 mg (0.947 mmol, 31.6%) 1,2-*O*-isopropylidene-5-*O*-(3,5-bis(trifluoromethyl)benzoyl)-α-D-*xylo*-hexofuranurono-6,3-lactone (**6c**) as a white solid after purification on SiO_2 (petrol ether / EtOAc 3:1). R_f (petrol ether / EtOAc, 2:1): 0.66; mp: 120-122 °C; IR (neat): 2998, 1823, 1748, 1619, 1380, 1281, 1243, 1177, 1132, 1084, 1028, 912, 844, 818, 769, 700, 682, 507, 438 cm^{-1}; ^{1}H-NMR (300 MHz, $CDCl_3$): 8.54 (s, 2 H), 8.12 (s, 1 H), 6.07 (d, *J* = 3.6, 1 H), 5.78 (d, *J* = 4.4, 1 H), 5.20 (dd, *J* = 4.3, 3.0, 1 H), 4.99 (d, *J* = 3.0, 1 H), 4.90 (d, *J* = 3.6, 1 H), 1.52 (s, 3 H), 1.36 (s, 3 H); ^{13}C-NMR (75 MHz, $CDCl_3$): 169.0, 162.9, 132.5 (q, *J* = 34.0), 130.5, 130.3 (d, *J* = 3.0), 127.3 (m), 122.6 (q, *J* = 273), 113.8, 107.0, 82.5, 82.4, 76.9, 71.0, 26.9, 26.5; ^{19}F-NMR (282 MHz, $CDCl_3$): -63.4; HRMS (EI) m/z calculated for $C_{18}H_{14}F_6O_7$ ($[M]^{\cdot+}$) 456.0644, found 456.0647.

6. Selective synthesis of monoacylated tartrate *6e*

The preparation of mono-benzoate **6e** was not straight-forward (Table 1). Reaction of (+)-DET (**8**) with 1.05 equiv. of 3,5-bis(trifluoromethyl)benzoic acid chloride gave only 5% of mono-benzoate **6** alongside with 45% of bis-benzoate **9** (entry 1). Modification of the reaction temperature gave almost identical results (entry 2 - 4). Omission of DMAP on the other hand and performance of the reaction at -78 °C was found to give a high ratio of mono-benzoylation over bis-benzoylation (entry 5 to 8).

Table 1: Benzoylation of (+)-DET (**8**).

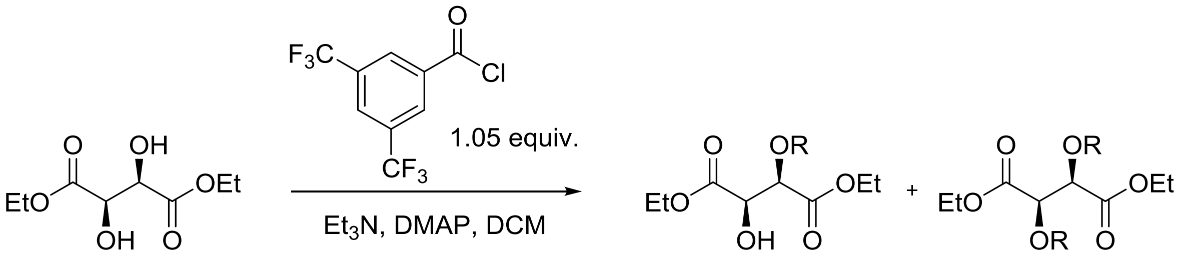

Entry	Scale	Conditions[a]	Monoester (%)	Diester (%)
1	5 mmol	0.1 M + neat, 0 °C	5	45
2	0.5 mmol	0.1 M + 0.1 M, -20 °C	17:83[b]	
3	0.5 mmol	0.1 M + 0.1 M, -40 °C	14:86[b]	
4	0.5 mmol	0.1 M + 0.1 M, -78 °C	14:86[b]	
5	0.5 mmol	0.1 M + 0.1 M, r.t., no DMAP	38:62[b]	
6	0.5 mmol	0.1 M + 0.1 M, 0 °C, no DMAP	44:56[b]	
6	0.5 mmol	0.1 M + 0.1 M, -40 °C, no DMAP	42:58[b]	
8	0.5 mmol	0.1 M + 0.1 M, -78 °C, no DMAP	47:53[b]	

[a]To a solution of **8** a solution of acid chloride was added dropwise at the indicated temperature. [b]^{1}H-NMR integration.

Despite the efforts to increase the amount of mono-benzoylation product **6e** the selectivity remained low with the acid chloride as acylation agent. Using 3,5-bis(trifluoromethyl)benzoic acid anhydride (**9**) in the presence of a Lewis acid catalyst largely increased the selectivity of

the benzoylation in favor of **6e** (Table 2). Without Lewis acid additive the reaction already favors mono-benzoylation product **6e** (entry 1). $YbCl_3$ increases the reaction speed as well as the selectivity (entry 2). While several other Lewis acids even slow down the reaction (entry 3 to 7), $CuCl_2$ again leads to high selectivity and even higher conversions (entry 8) compared to $YbCl_3$. Lowering the amount of $YbCl_3$ leads to longer reaction times, slightly higher conversion rates, and diminished selectivity (entry 9 and 10) while in contrast lowering the amount of $CuCl_2$ leads to decreased conversions but increased selectivities (entry 11). The assumption that at lower conversions the selectivity is higher is self-evident – nevertheless the conversion reaches its final point at the time indicated and no further rise was observed after prolonged times. Due to the considerably lower cost of $CuCl_2$ in comparison to $YbCl_3$, $CuCl_2$ was ultimately used for a reaction in a larger scale (entry 12). Surprisingly the large scale reaction took much longer than the reaction on small scale to

Table 2: Benzoylation of (+)-DET (**8**) with benzoic acid anhydride **4** and Lewis acid catalysis.

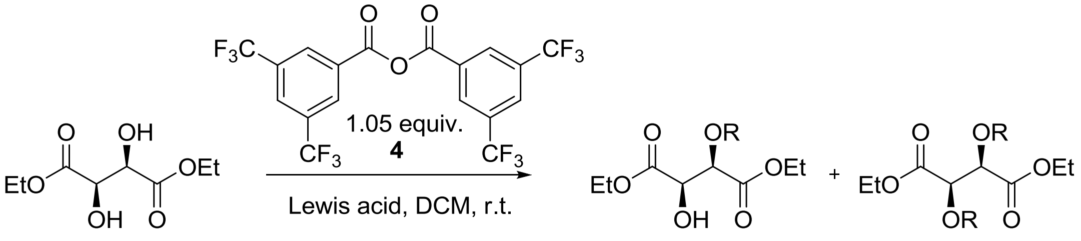

Entry	Lewis acid	Time	Conversion[a]	Monoester (%)	Diester (%)
1	-	68 h	66%	71:39[b]	
2	$YbCl_3$ (10 mol%)	20 h	62%	>95:5[b]	
3	$FeCl_3$ (10 mol%)	2 h	<5%	-	
4	$ZnCl_2$ (10 mol%)	2 h	<5%	-	
5	$AlCl_3$ (10 mol%)	2 h	<5%	-	
6	$TiCl_4$ (10 mol%)	2 h	<5%	-	
7	$NiCl_2$ (10 mol%)	2 h	<5%	-	
8	$CuCl_2$ (10 mol%)	68 h	82%	94:6[b]	
9	$YbCl_3$ (1 mol%)	68 h	80%	93:7[b]	
10	$YbCl_3$ (0.1 mol%)	140 h	75%	83:17[b]	
11	$CuCl_2$ (1 mol%)	68 h	56%	>95:5[b]	
12	$CuCl_2$ (10 mol%)[c]	7d + 7d (reflux)	81%	77%	2%

[a]Conversion of **8** on a 0.5 mmol scale. [b]^{1}H-NMR integration. [c]6 mmol scale.

reach comparable conversions. The exact nature of this effect is unknown but might be to the inhomogeneous nature of the reaction ($CuCl_2$ is not fully soluble in DCM at a loading of 10 mol%). Nevertheless practical reaction conditions for the synthesis of **6e** were found:

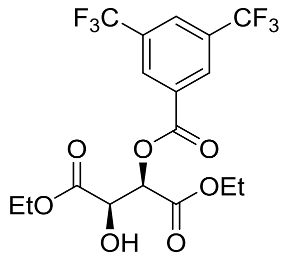

(2*R*,3*R*)-Diethyl 2-(3,5-bis(trifluoromethyl)benzoyloxy)-3-hydroxysuccinate (6e): A 100 mL Schlenk flask equipped with a magnetic stir bar was charged with $CuCl_2$ (81.3 mg, 0.605 mmol, 0.100 equiv.), (2R,3R)-diethyl 2,3-dihydroxysuccinate (**8**, 1.25 g, 6.05 mmol, 1.00 equiv.) and DCM (50 mL). A solution of 3,5-bis(trifluoromethyl)benzoic anhydride in DCM (34 mL, 0.18 M, 6.1 mmol, 1.0 equiv.) was added and the mixture was stirred at ambient temperature for one week and refluxed for another week. The reaction mixture was transferred to a separation funnel with 150 mL EtOAc, washed twice with 50 mL 10% Na_2CO_3 (aq.), 50 mL water, and 50 mL brine. The combined aqueous phased were boiled up, cooled back to room temperature and acidified with conc. HCl (aq.) upon which the white precipitate was filtered and washed with 50 mL water to give 1.31 g (5.08 mmol, 67% based on benzoate that was employed and not incorporated in the product) 3,5-bis(trifluoromethyl)benzoic acid. The organic phases were dried over Na_2SO_4, evaporated and the resulting solid was purified on SiO_2 (petrol ether / EtOAc, 6:1 to 2:1) to give 2.07 g (4.63 mmol, 76.5%) (2R,3R)-diethyl 2-(3,5-bis(trifluoromethyl)benzoyloxy)-3-hydroxysuccinate a white solid. R_f (petrol ether / EtOAc, 4:1): 0.34; mp: 98 °C; IR (neat): 3378, 2986, 2944, 1737, 1707, 1627, 1391, 1373, 1280, 1230, 1177, 1124, 1050, 917, 847, 768, 700, 681, 586, 538, 495, 439 cm^{-1}; ^{1}H-NMR (300 MHz, $CDCl_3$): 8.48 (s, 2 H), 8.10 (s, 1 H), 5.72 (d, *J* = 2.3, 1 H), 4.89 (d, *J* = 2.0, 1 H), 4.32 (q, *J* = 7.1, 2 H), 4.27 (q, *J* = 7.1, 2 H), 3.45 (brs, 1 H), 1.32 (t, *J* = 7.1, 3 H), 1.23 (t, *J* = 7.1, 3 H); ^{13}C-NMR (75 MHz, $CDCl_3$): 170.6, 165.9, 162.8, 132.4 (q, *J* = 34.0), 131.0, 130 (d, *J* = 3.0), 127.1 (m), 122.7 (q, *J* = 274), 74.3, 70.5, 62.9, 62.6, 14.1; ^{19}F-NMR (282 MHz, $CDCl_3$): -63.5; HRMS (EI) m/z calculated for $C_{17}H_{17}F_6O_7$ ($[M+H]^+$) 447.0873, found 447.0868.

7. General procedure for photoreactions (*GPIII*)

General procedure for deoxygenations of (trifluoromethyl)benzoate esters (***GPIII***): A Schlenk tube was charged with [Ir(ppy)$_2$(dtb-bpy)](PF$_6$) (3.7 mg, 4.0 µmol, 2.0 mol%), (trifluoromethyl)benzoate ester (0.200 mmol, 1.00 equiv.), sealed with a screw-cap and subsequently evacuated and backfilled with N_2 (3x). MeCN (5 ml), ^{i}Pr$_2$NEt (70 µL, 52 mg, 0.40 mmol, 2.0 equiv.), and degassed water (0.36 mL, 0.36 g, 20 mmol, 100 equiv.) was added and the reaction mixture was magnetically stirred until a homogeneous solution was obtained. The reaction mixture was degassed by freeze-pump-thaw (5x) and the screw-cap was replaced with a Teflon sealed inlet for a glass rod, through which irradiation with a 455 nm high power LED took place from above (1 h for every 0.2 mmol of benzoate) while the reaction was magnetically stirred and heated in an aluminum block from below. Afterwards the reaction mixture was diluted with 20 mL Et_2O, washed with 10 mL 10% Na_2CO_3, 10 mL H_2O, 10 mL brine, and dried over Na_2SO_4. The combined aqueous phases were acidified with HCl (conc.) upon which (trifluoromethyl)benzoic acid precipitated, which was collected by filtration and washed with water. After evaporation of the organic phase, the product was purified by filtration through a short plug of flash silica gel with a mixture of petrol ether and ethyl acetate.

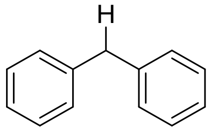

Diphenylmethane (4a):[14,15] Following general procedure ***GPIII*** using benzhydryl 3,5-bis(trifluoromethyl)benzoate (**3a**) (424 mg, 1.00 mmol, 1.00 equiv.), [Ir(ppy)$_2$(dtb-bpy)](PF$_6$) (18.3 mg, 20.0 µmol, 2.00 mol%), ^{i}Pr$_2$NEt (348 µL, 258 mg, 2.00 mmol, 2.00 equiv.), degassed water (1.8 mL, 1.8 g, 0.10 mol, 100 equiv.), and MeCN (25 mL) gave 160 mg (0.949 mmol, 95%) of a colorless oil after filtration through SiO_2 with petrol ether. ^{1}H NMR (300 MHz, $CDCl_3$): 7.33-7.16 (m, 10 H), 4.00 (s, 2 H).

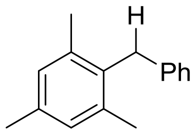

2-Benzyl-1,3,5-trimethylbenzene (4b):[16] Following general procedure ***GPIII*** gave 38.4 mg (0.182 mmol, 91%) of a colorless oil after filtration through SiO_2 with petrol ether. ^{1}H-NMR (400 MHz, $CDCl_3$): 7.29-7.11 (m, 3 H), 7.07-6.97 (m, 2 H), 6.91 (s, 2 H), 4.03 (s, 2 H), 2.31 (s, 3 H), 2.22 (s, 6 H).

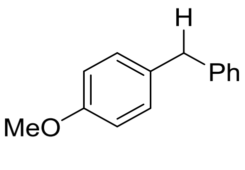

1-Benzyl-4-methoxybenzene (4c):[16] Following general procedure ***GPIII*** gave 34.4 mg (0.174 mmol, 87%) of a colorless oil after filtration through

SiO_2 with petrol ether. ^{1}H-NMR (400 MHz, $CDCl_3$): 7.32-7.25 (m, 2 H), 7.23-7.16 (m, 3 H), 7.14-7.08 (m, 2 H), 6.85-6.81 (m, 2 H), 3.94 (s, 2 H), 3.79 (s, 3 H).

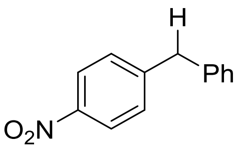

1-Benzyl-4-nitrobenzene (4d):[17] Following general procedure ***GPIII*** gave 38.7 mg (0.181 mmol, 91%) of a yellow oil after purification on SiO_2 (petrol ether / EtOAc, 30:1). ^{1}H-NMR (300 MHz, $CDCl_3$): 8.19-8.09 (m, 2 H), 7.39-7.14 (7 H), 4.08 (s, 2 H).

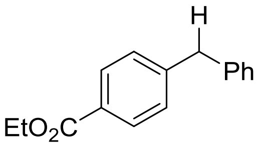

Ethyl 4-benzylbenzoate (4e):[14] Following general procedure ***GPIII*** gave 44.7 mg (0.186 mmol, 93%) of a colorless oil after filtration through SiO_2 with petrol ether / EtOAc = 50:1. ^{1}H-NMR (400 MHz, $CDCl_3$): 7.99-7.94 (m, 2 H), 7.33-7.15 (m, 7 H), 4.36 (q, J = 7.2, 2 H), 4.04 (s, 2 H), 1.28 (t, J = 7.2, 3 H).

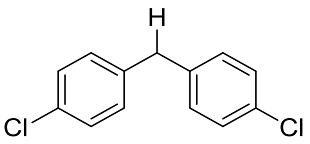

Bis(4-chlorophenyl)methane (4f):[18] Following general procedure ***GPIII*** gave 43.6 mg (0.183 mmol, 92%) of a colorless oil after filtration through SiO_2 with petrol ether. ^{1}H-NMR (300 MHz, $CDCl_3$): 7.30-7.22 (m, 4 H), 7.13-7.05 (m, 4 H), 3.92 (s, 2 H).

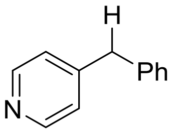

4-Benzylpyridine (4g):[16] Following general procedure ***GPIII*** gave 29.1 mg (0.171 mmol, 86%) of a colorless oil after purification on SiO_2 (petrol ether / EtOAc, 2:1). ^{1}H-NMR (300 MHz, $CDCl_3$): 8.51 (s, 2 H), 7.38-7.08 (m, 7 H), 3.98 (s, 2 H).

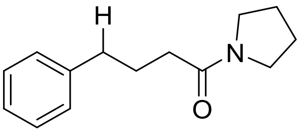

4-Phenyl-1-(pyrrolidin-1-yl)butan-1-one (4i): Following general procedure ***GPIII*** gave 34.5 mg (0.158 mmol, 79%) of a slightly yellow oil after purification on SiO_2 (petrol ether / EtOAc, 1:1). ^{1}H-NMR (300 MHz, $CDCl_3$): 7.30-7.24 (m, 2 H),7.21-7.15 (m, 3 H), 3.45 (t, J = 7.0, 2 H), 3.32 (t, J = 6.7, 2 H), 2.68 (t, J = 7.5, 2H), 2.26 (t, J = 7.3, 2 H), 2.05-1.77 (m, 6 H).

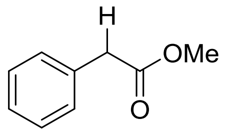

Methyl 2-phenylacetate (4j):[19] Following general procedure ***GPIII*** using 2-methoxy-2-oxo-1-phenylethyl 3,5-bis(trifluoromethyl)benzoate (**6j**) (305 mg, 0.750 mmol, 1.00 equiv.), [Ir(ppy)$_2$(dtb-bpy)](PF$_6$) (13.9 mg, 15.2 µmol, 2.02 mol%), ^{i}Pr$_2$NEt (209 µL, 1.50 mmol, 2.00 equiv.), degassed water (1.35 mL, 1.35 g, 75.0 mmol, 100 equiv.), and MeCN (19 mL) gave 93.7 mg (0.624 mmol, 83%) of a colorless oil after filtration through SiO$_2$ with petrol ether / EtOAc = 10:1. ^{1}H-NMR (300 MHz, CDCl$_3$): 7.41-7.21 (m, 5 H), 3.70 (s, 3 H), 3.64 (s, 2 H).

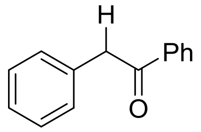

1,2-Diphenylethanone (4k):[20] Following general procedure ***GPIII*** gave 26.3 mg (0.134 mmol, 67%) of a colorless oil after filtration through SiO$_2$ with petrol ether / EtOAc = 25:1. ^{1}H-NMR (400 MHz, CDCl$_3$): 8.07-8.00 (m, 2 H), 7.60-7.53 (m, 1 H), 7.50-7.46 (m, 2 H), 7.37-7.31 (m, 2 H), 7.30-7.23 (m, 3 H), 4.30 (s, 2 H).

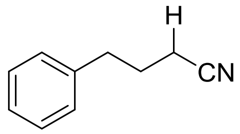

4-Phenylbutanenitrile (7a):[21] Following general procedure ***GPIII*** gave 24.9 mg (0.171 mmol, 86%) of a colorless oil after filtration through SiO$_2$ with petrol ether / EtOAc = 6:1. ^{1}H-NMR (400 MHz, CDCl$_3$): 7.36-7.28 (m, 2 H), 7.26-7.16 (m, 3 H), 2.78 (t, *J* = 7.4, 2 H), 2.32 (t, *J* = 7.1, 2 H), 1.99 (quint, J = 7.2, 2 H).

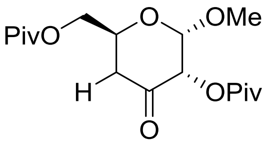

((2*S*,5*S*,6*S*)-6-Methoxy-4-oxo-5-(pivaloyloxy)tetrahydro-2*H*-pyran-2-yl)methyl pivalate (7b):[12] Following general procedure ***GPIII*** using (2R,3R,5S,6S)-6-methoxy-4-oxo-5-(pivaloyloxy)-2-(pivaloyloxymethyl)tetrahydro-2H-pyran-3-yl 3,5-bis(trifluoromethyl)benzoate (**6b**, 120 mg, 0.200 mmol, 1.00 equiv.) gave 54.5 mg (0.158 mmol, 79%) of a white solid after column chromatography on flash silica gel with petrol ether/EtOAc = 3:1. ^{1}H-NMR (300 MHz, CDCl$_3$): 5.19 (d, *J* = 4.0, 1 H), 5.07 (d, *J* = 4.0, 1 H), 4.28-4.07 (m, 3 H), 3.37 (s, 3 H), 2.64-2.40 (m, 2 H), 1.21 (s, 9 H), 1.16 (s, 9 H).

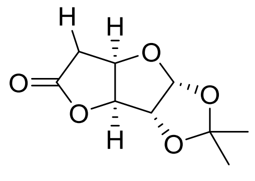

5-Deoxy-1,2-*O*-isopropylidene-α-D-*xylo*-hexofuranurono-6,3-lactone (7c):[22] Following general procedure ***GPIII*** using 1,2-*O*-isopropylidene-5-*O*-(3,5-bis(trifluoromethyl)benzoyl)-α-D-xylo-hexofuranurono-6,3-lactone (**6c**, 91.3 mg, 0.200 mmol, 1.00 equiv.)

gave 5.6 mg (0.028 mmol, 14%) of a colorless oil after column chromatography on flash silica gel with petrol ether/EtOAc = 2:1. ^{1}H-NMR (300 MHz, $CDCl_3$): 5.90 (d, J = 3.8, 1 H), 4.94 (q, J = 2.8, 1 H), 4.77 (t, J = 4.1, 2 H), 2.66 (d, J = 2.8, 2 H), 1.44 (s, 3 H), 1.28 (s, 3 H).

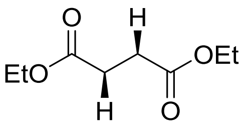

Diethyl succinate (7d):[15] After following general procedure ***GPIII*** using (2R,3R)-diethyl 2,3-bis(3,5-bis(trifluoromethyl)benzoyloxy)succinate (**6d**) (686 mg, 1.00 mmol, 1.00 equiv.), $[Ir(ppy)_2(dtb\text{-}bpy)](PF_6)$ (18.3 mg, 20.0 µmol, 2.00 mol%), $^iPr_2NEt$ (1.75 mL, 1.30 g, 10.0 mmol, 10.0 equiv.), degassed water (1.8 mL, 1.8 g, 0.10 mol, 100 equiv.), and MeCN (25 mL), 1,4-dimethoxybenzene (138 mg, 1.00 mmol, 1.00 equiv.) was added to the crude mixture and an aliquot was subjected to ^{1}H-NMR analysis. The yield of diethyl succinate (**8l**) was determined to be 69%.

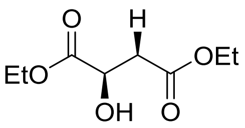

(*R*)-Diethyl 2-hydroxysuccinate (7e):[15] Following general procedure ***GPIII*** using (2R,3R)-diethyl 2-(3,5-bis(trifluoromethyl)benzoyloxy)-3-hydroxysuccinate (**6e**) (446 mg, 1.00 mmol, 1.00 equiv.), $[Ir(ppy)_2(dtb\text{-}bpy)](PF_6)$ (18.3 mg, 20.0 µmol, 2.00 mol%), $^iPr_2NEt$ (348 µL, 258 mg, 2.00 mmol, 2.00 equiv.), degassed water (1.8 mL, 1.8 g, 0.10 mol, 100 equiv.), and MeCN (25 mL) gave 188 mg (0.989 mmol, 99%) of a colorless oil after filtration through a short plug of flash silica gel with petrol ether/EtOAc = 1:1. ^{1}H-NMR (300 MHz, $CDCl_3$): 4.47 (brs, 1 H), 4.34-4.06 (m, 4 H), 3.23 (brs, 1 H), 2.91-2.73 (m, 2 H), 1.30 (t, *J* = 7.2, 3 H), 1.26 (t, *J* = 7.2, 3 H).

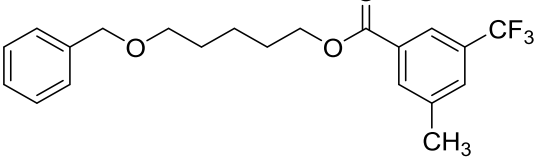

5-(Benzyloxy)pentyl 3-methyl-5-(trifluoromethyl)benzoate (12): Following general procedure ***GPIII*** using 5-(benzyloxy)pentyl 3,5-bis(trifluoromethyl)-benzoate (**10**) (434 mg, 1.00 mmol, 1.00 equiv.), $[Ir(ppy)_2(dtb\text{-}bpy)](PF_6)$ (18.3 mg, 20.0 µmol, 2.00 mol%), $^iPr_2NEt$ (1.74 mL, 1.29 g, 10.0 mmol, 10.0 equiv.), degassed water (1.8 mL, 1.8 g, 0.10 mol, 100 equiv.), and MeCN (25 mL) gave 293 mg (0.770 mmol, 77%) of a colorless oil after filtration through SiO_2 with petrol ether. R_f (petrol ether / EtOAc, 6:1): 0.40; IR (neat): 2943, 2860, 1722, 1612, 1455, 1390, 1352, 1247, 1199, 1166, 1123, 973, 855, 769, 734, 694, 614 cm^{-1}; ^{1}H-NMR (300 MHz, $CDCl_3$): 8.09 (s, 1 H), 8.02 (s, 1 H), 7.61 (s, 1 H), 7.36-7.23 (m, 5 H), 4.51 (s, 2 H), 4.35 (t, J=6.7, 2 H), 3.51 (t, J=6.4, 2 H), 2.47 (s, 3 H), 1.86-1.75 (m, 2 H), 1.75-1.65 (m, 2 H), 1.60-1.48 (m, 2 H); ^{13}C-NMR (75 MHz, $CDCl_3$): 165.6, 139.3, 138.5, 133.4, 131.2, 130.7, 130.0

(m), 128.4, 127.7, 127.6, 123.8 (q, *J* = 274), 123.7 (m), 73.0, 70.1, 65.5, 29.4, 28.5, 22.8, 21.3; ^{19}F-NMR (376 MHz, $CDCl_3$): -62.7; HRMS (EI) m/z calculated for $C_{21}H_{24}F_3O_3$ ($[M+H]^+$) 381.1672, found 381.1668.

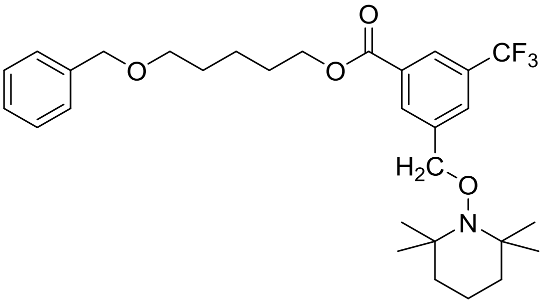

5-(Benzyloxy)pentyl 3-((2,2,6,6-tetramethylpiperidin-1-yloxy)methyl)-5-(trifluoromethyl)benzoate (13) as a 3:1 mixture with 12: Following general procedure ***GPIII*** using 5-(benzyloxy)pentyl 3,5-bis(trifluoromethyl)benzoate (**10**) (86.9 mg, 0.200 mmol, 1.00 equiv.), $[Ir(ppy)_2(dtb\text{-}bpy)](PF_6)$ (3.7 mg, 4.0 µmol, 2.00 mol%), $^{i}Pr_2NEt$ (348 µL, 258 mg, 2.00 mmol, 10.0 equiv.), degassed water (360 µL, 360 mg, 20.0 mmol, 100 equiv.), TEMPO (28.2 mg, 0.180 mmol, 0.900 equiv.), and MeCN (5 mL) gave 30.1 mg of a colorless oil containing **13** and **12** (3:1, ^{1}H-NMR integration) after purification on SiO_2 (petrol ether / EtOAc, 100:0 to 10:1). **13**: ^{1}H-NMR (300 MHz, $CDCl_3$): 8.22-8.15 (m, 2 H), 7.79 (s, 1 H), 7.39-7.22 (m, 5 H), 4.91 (s, 2 H), 4.51 (s, 2 H), 4.42-4.30 (m, 2 H), 3.56-3.43 (m, 2 H), 1.90-1.44 (m, 12 H), 1.22 (s, 6 H), 1.16 (s, 6 H); ^{19}F-NMR (282 MHz, $CDCl_3$): -63.1; HRMS (EI) m/z calculated for $C_{30}H_{41}F_3NO_4$ ($[M+H]^+$) 536.2982, found 536.2993.

8. In-situ acylation and deoxygenations in a microreactor

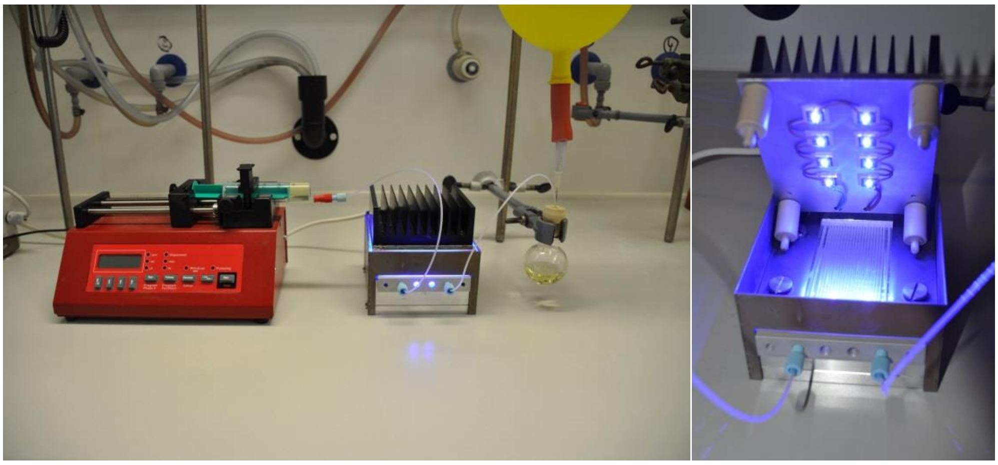

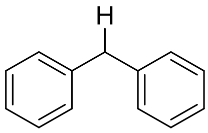

Diphenylmethane (4a):[14,15] A 100 mL Schlenk flask equipped with a magnetic stir bar was charged with diphenylmethanol (**14**, 184 mg, 1.00 mmol, 1.00 equiv.) and 3,5-bis(trifluoromethyl)benzoic anhydride (**9**, 548 mg, 1.10 mmol, 1.10 equiv.), sealed with a screw-cap and subsequently evacuated and backfilled with N_2 (3x). MeCN (5 mL) and Et_3N (0.70 mL, 0.51 g, 5.0 mmol, 5.0 equiv.) were added and the mixture was stirred at 60 °C for 18 h. The reaction mixture was diluted with 16 mL MeCN and 1.8 mL water and [Ir(ppy)$_2$(dtb-bpy)](PF$_6$) (0.9 mg, 1 µmol, 0.1 mol%) were added. The reaction mixture was degassed by sparging with N_2 for 30 min and pumped through a micro reactor equipped with 8 LED's at a flowrate of 4.0 mL/h (0.17 mmol/h) *via* a syringe pump. The reaction mixture was evaporated, 20 mL Et_2O was added and the mixture was subsequently washed with 2 x 10 mL 10% Na_2CO_3, 10 mL H_2O, and dried over Na_2SO_4. The combined aqueous phases were acidified with 6 M HCl upon which a white solid precipitated, which was collected by filtration and washed with water to give 3,5-bis(trifluoromethyl)benzoic acid (513 mg, 1.99 mmol, 90%). After evaporation of the organic phase, the obtained oil was purified by filtration through a short plug of SiO_2 with petrol ether to give 153 mg (0.911 mmol, 91%) of diphenylmethane (**4a**) as a colorless oil.

9. References

(1) Sedelmeier, J.; Bolm, C. *J. Org. Chem.* **2007**, *72*, 8859.

(2) Kim, J.; De Castro, K. a.; Lim, M.; Rhee, H. *Tetrahedron* **2010**, *66*, 3995.

(3) Ohta, S.; Hayakawa, S.; Moriwaki, H.; Harada, S.; Okamoto, M. *Chem. Pharm. Bull.* **1986**, *34*, 4916.

(4) Leazer, J. L.; Cvetovich, R.; Tsay, F.; Dolling, U.; Vickery, T.; Bachert, D. *J. Org. Chem.* **2003**, *68*, 3695.

(5) Giencke, A.; Lackner, H. *Liebigs Ann. Chem.* **1990**, *6*, 569.

(6) Kodama, S.; Hashidate, S.; Nomoto, A.; Yano, S.; Ueshima, M.; Ogawa, A. *Chem. Lett.* **2011**, *40*, 495.

(7) Boymond, L.; Rottländer, M.; Knochel, P. *Angew. Chem. Int. Ed.* **1998**, *37*, 1701.

(8) Padmanaban, M.; Biju, A. T.; Glorius, F. *Org. Lett.* **2011**, *13*, 98.

(9) Cheng, M.; Bakac, A. *Dalt. Trans.* **2007**, *56*, 2077.

(10) Young, I. S.; Kerr, M. A. *J. Am. Chem. Soc.* **2007**, *129*, 1465.

(11) Deleuze-Masquéfa, C.; Gerebtzoff, G.; Subra, G.; Fabreguettes, J.-R.; Ovens, A.; Carraz, M.; Strub, M.-P.; Bompart, J.; George, P.; Bonnet, P.-A. *Bioorg. Med. Chem.* **2004**, *12*, 1129.

(12) Rauter, A. P.; Fernandes, A. C.; Czernecki, S.; Valery, J.-M. *J. Org. Chem.* **1996**, *61*, 3594.

(13) Bashyal, B. P.; Chow, H.-F.; Fellows, L. E.; Fleet, G. W. J. *Tetrahedron* **1987**, *43*, 415.

(14) Haberberger, M.; Someya, C. I.; Company, A.; Irran, E.; Enthaler, S. *Catal. Lett.* **2012**, *142*, 557.

(15) Material was identical with an authentic sample.

(16) Henry, N.; Enguehard-Gueiffier, C.; Thery, I.; Gueiffier, A. *Eur. J. Org. Chem.* **2008**, 4824.

(17) Inés, B.; SanMartin, R.; Moure, M. J.; Domínguez, E. *Adv. Synth. Catal.* **2009**, *351*, 2124.

(18) Endo, K.; Ishioka, T.; Ohkubo, T.; Shibata, T. *J. Org. Chem.* **2012**, *77*, 7223.

(19) Harker, W. R. R.; Carswell, E. L.; Carbery, D. R. *Org. Lett.* **2010**, *12*, 3712.

(20) Li, L.; Cai, P.; Guo, Q.; Xue, S. *J. Org. Chem.* **2008**, *73*, 3516.

(21) Yin, W.; Wang, C.; Huang, Y. *Org. Lett.* **2013**, *15*, 1850.

(22) Rauter, A. P.; Fernandes, A. C.; Figueiredo, J. A. *J. Carbohydr. Chem.* **1998**, *17*, 1037.

10. Spectra of new compounds

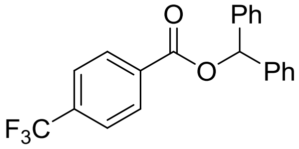

1

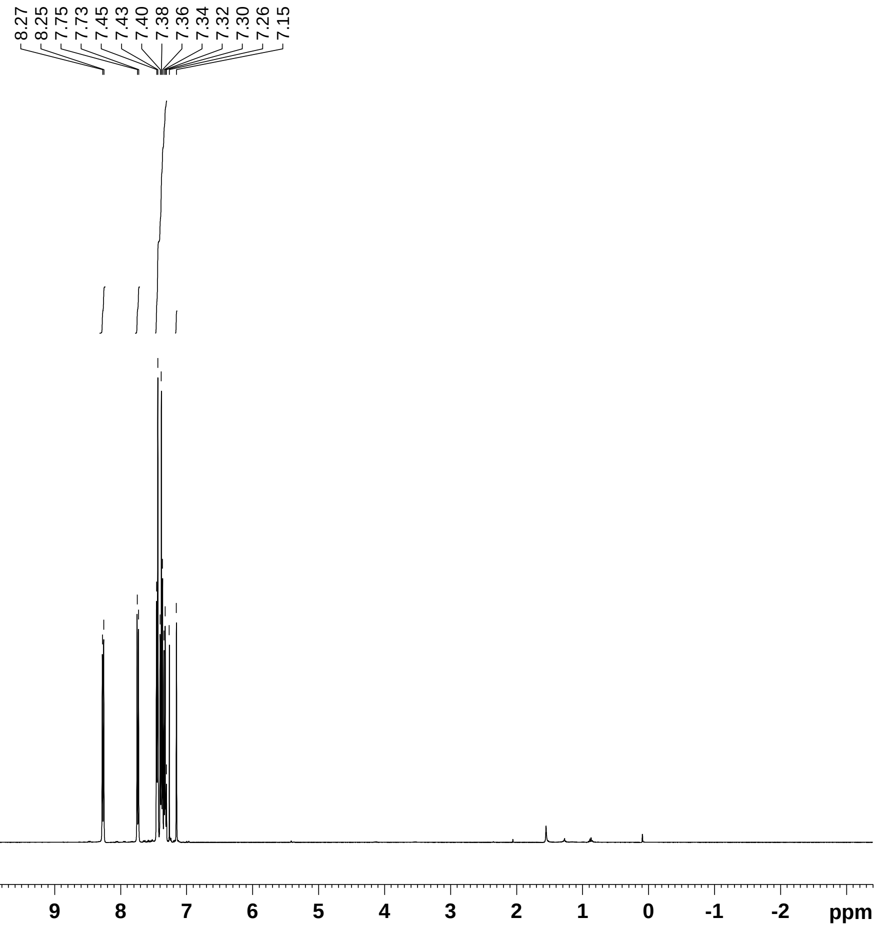

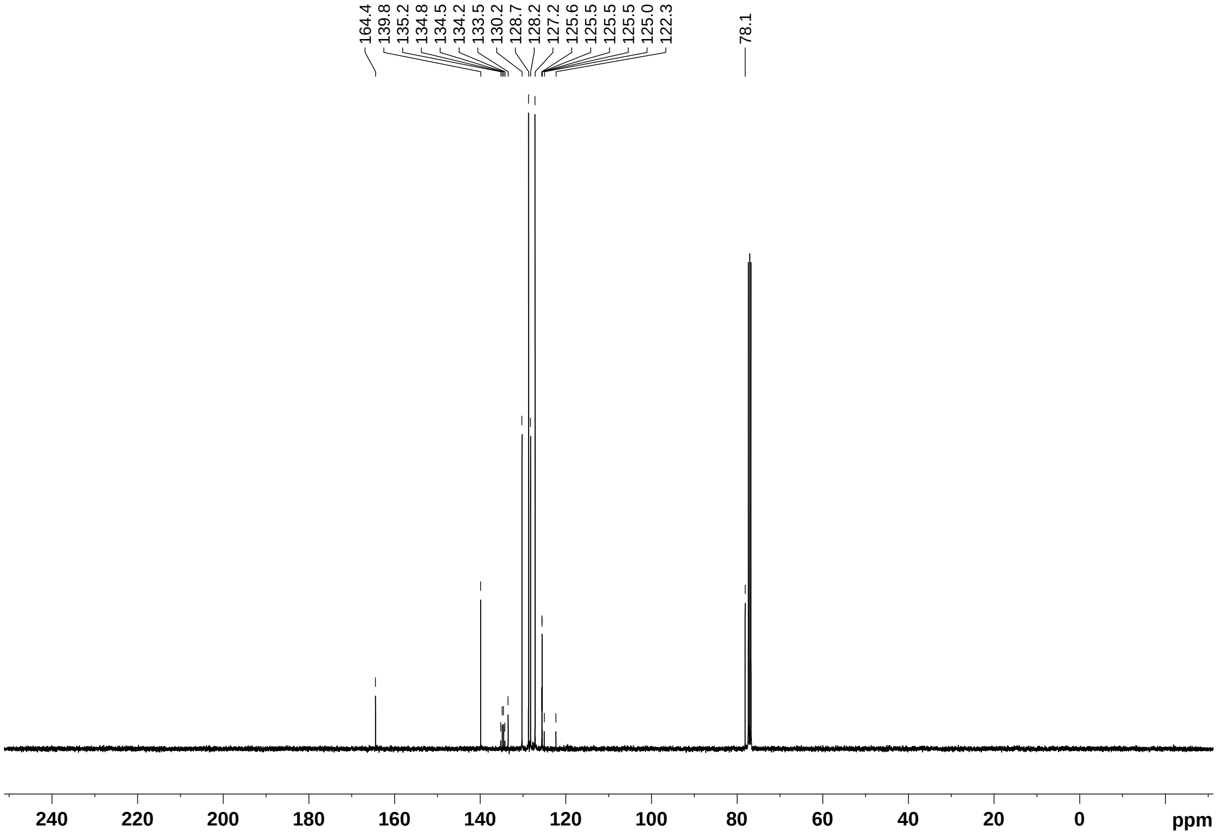

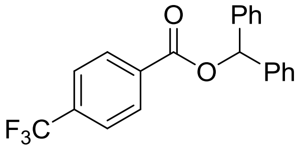

1

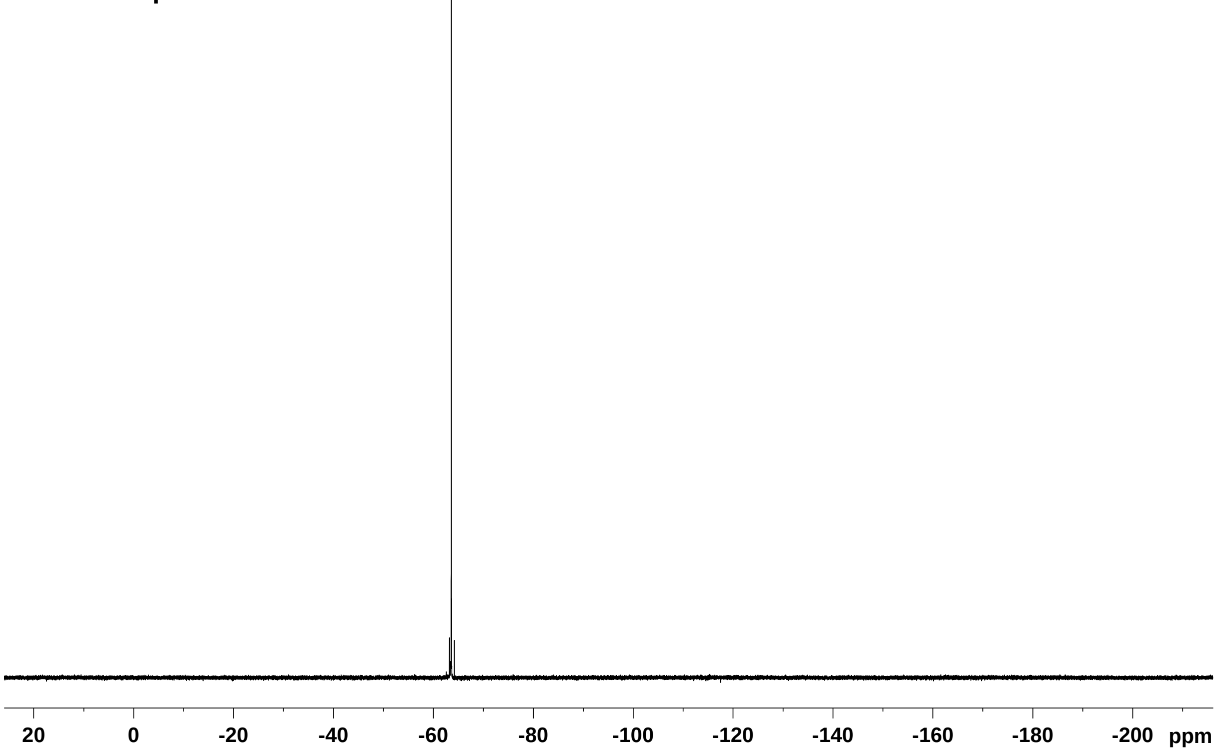

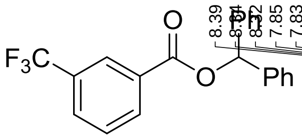

F3C
O
O
Ph
Ph

2

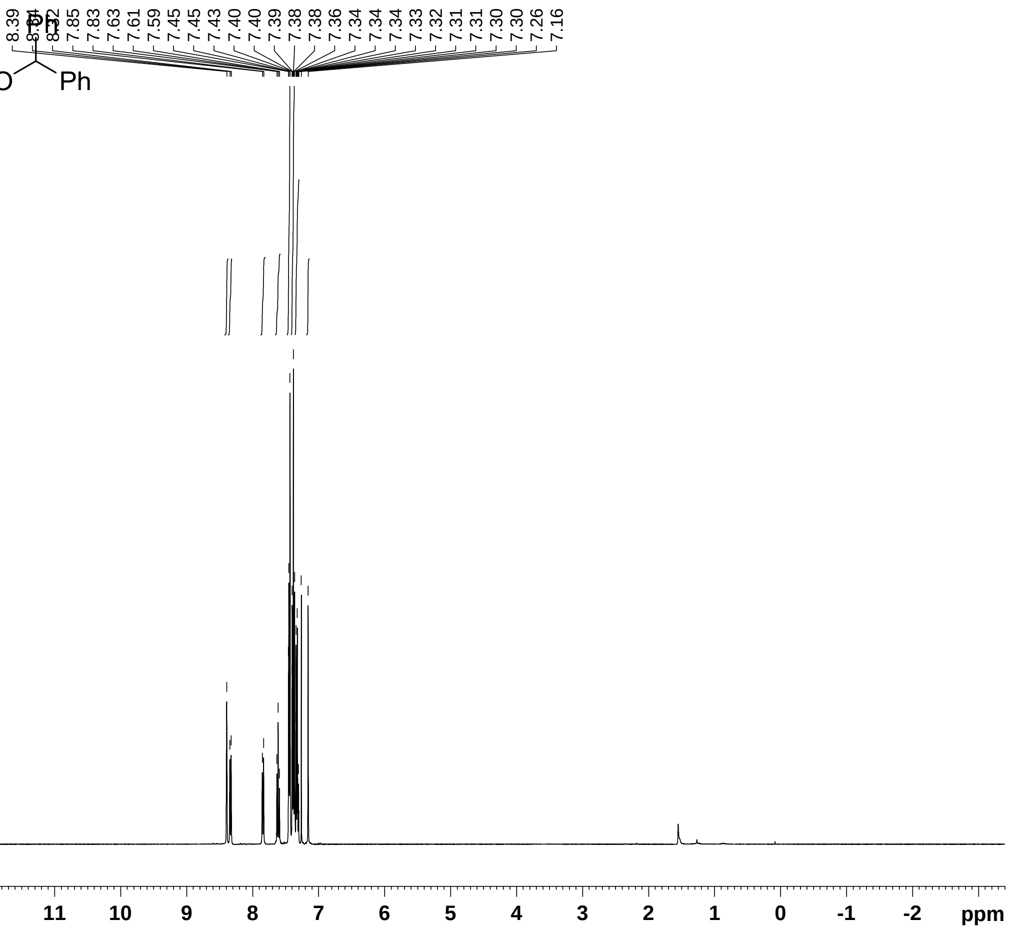

8.39
7.85
7.83
7.63
7.61
7.59
7.45
7.45
7.43
7.40
7.40
7.39
7.38
7.38
7.36
7.34
7.34
7.34
7.33
7.32
7.31
7.31
7.30
7.30
7.26
7.16
14 13 12 11 10 9 8 7 6 5 4 3 2 1 0 -1 -2 ppm

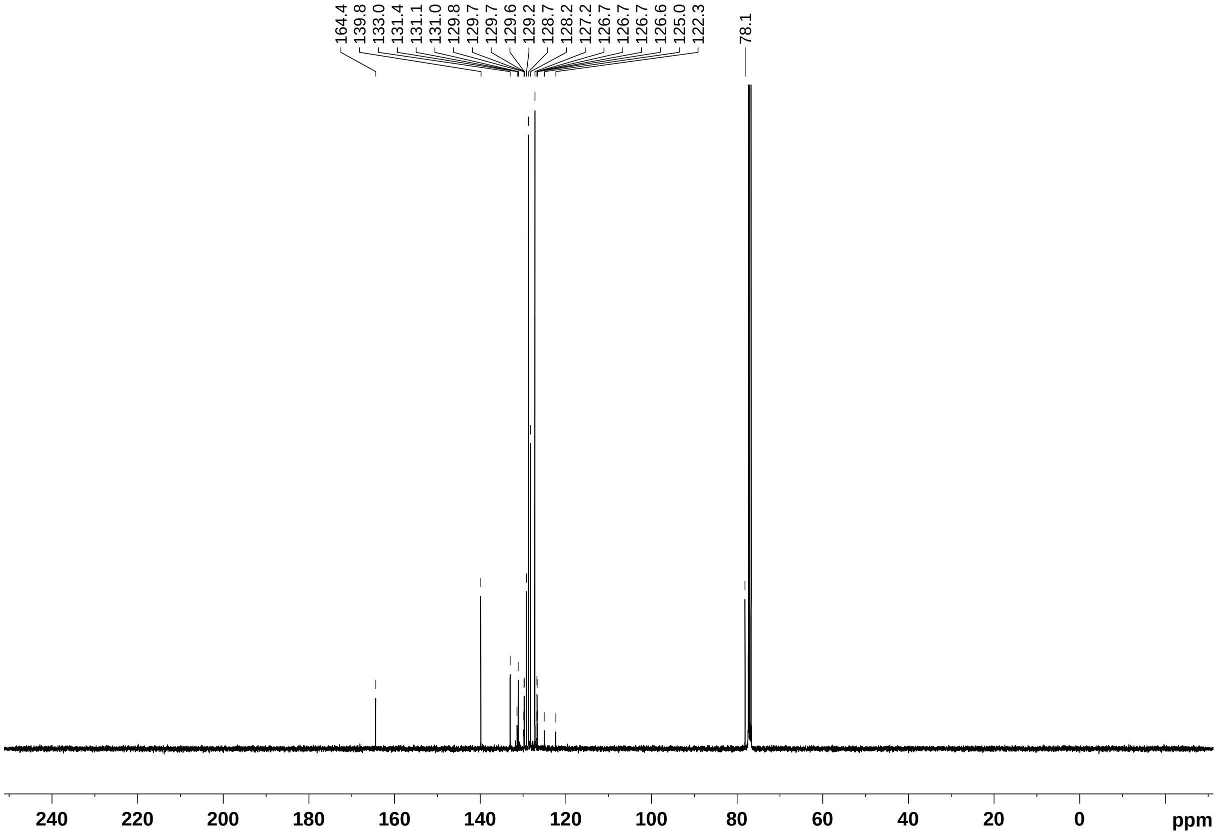

164.4
139.8
133.0
131.4
131.1
131.0
129.8
129.7
129.7
129.6
129.2
128.7
128.2
127.2
126.7
126.7
126.7
126.6
125.0
122.3
78.1
240 220 200 180 160 140 120 100 80 60 40 20 0 ppm

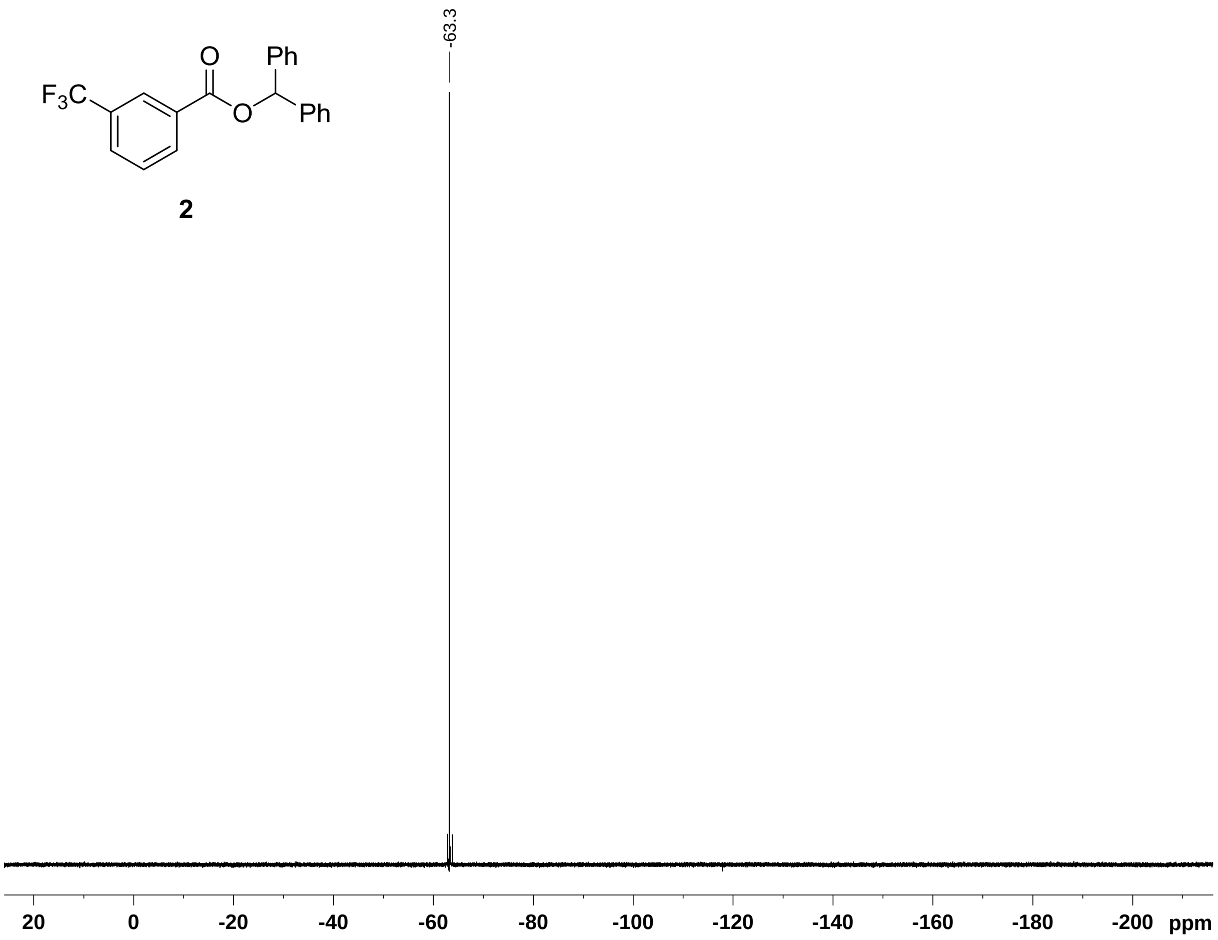

2

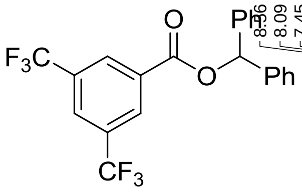
O
Ph
F3C
O
Ph
CF3

3

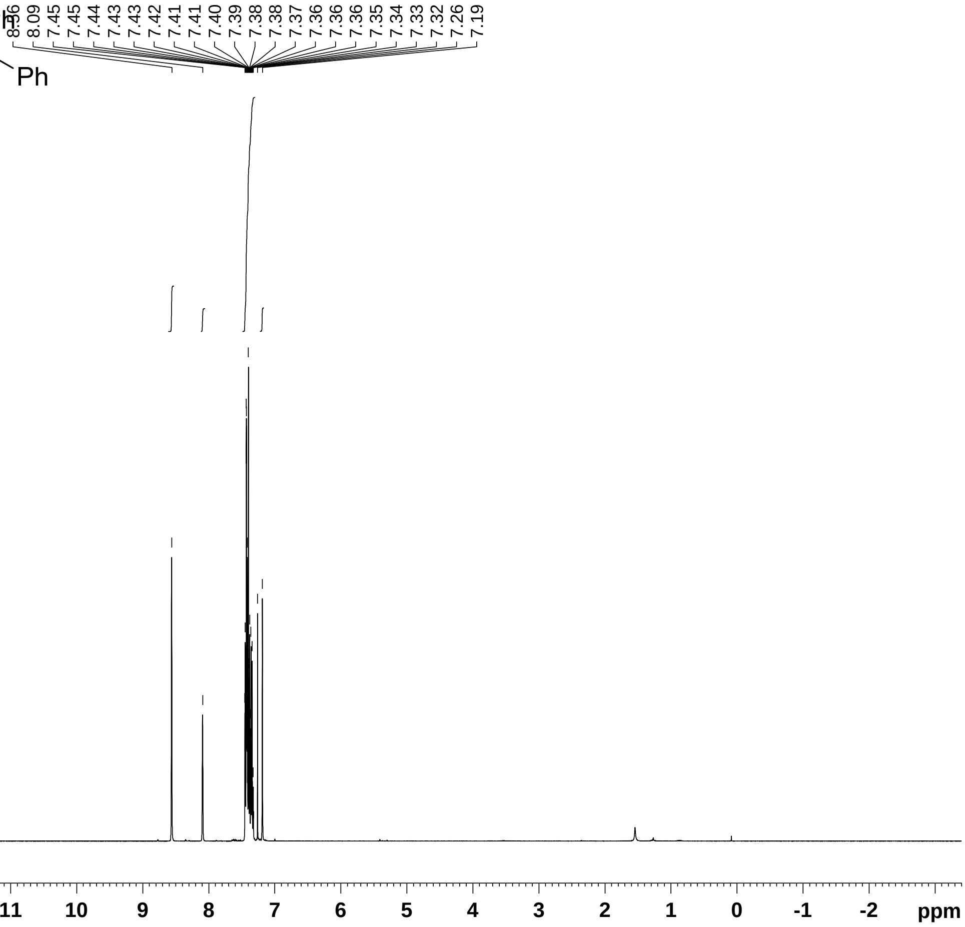
8.56
8.09
7.45
7.45
7.44
7.43
7.43
7.42
7.41
7.41
7.40
7.39
7.38
7.38
7.37
7.36
7.36
7.36
7.35
7.34
7.33
7.32
7.26
7.19
14 13 12 11 10 9 8 7 6 5 4 3 2 1 0 -1 -2 ppm

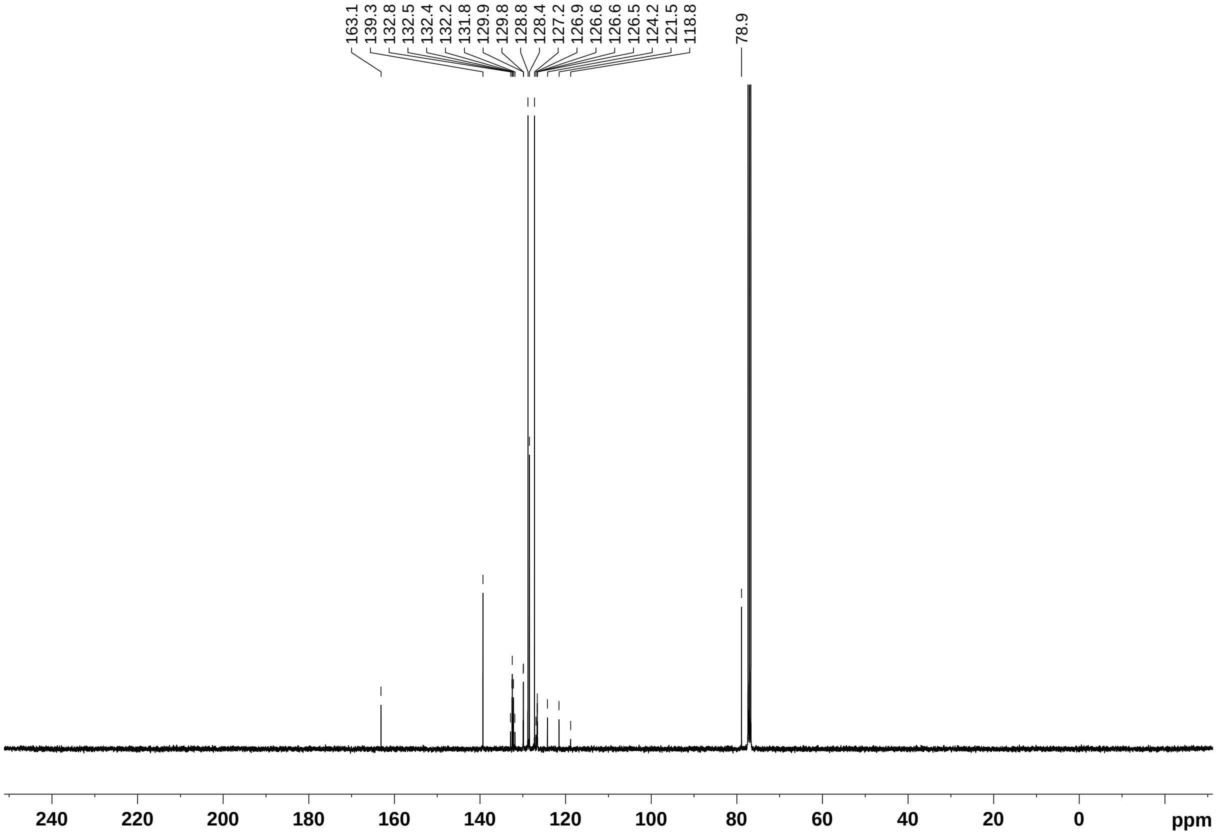
163.1
139.3
132.8
132.5
132.4
132.2
131.8
129.9
129.8
128.8
128.4
127.2
126.9
126.6
126.6
126.5
124.2
121.5
118.8
78.9
240 220 200 180 160 140 120 100 80 60 40 20 0 ppm

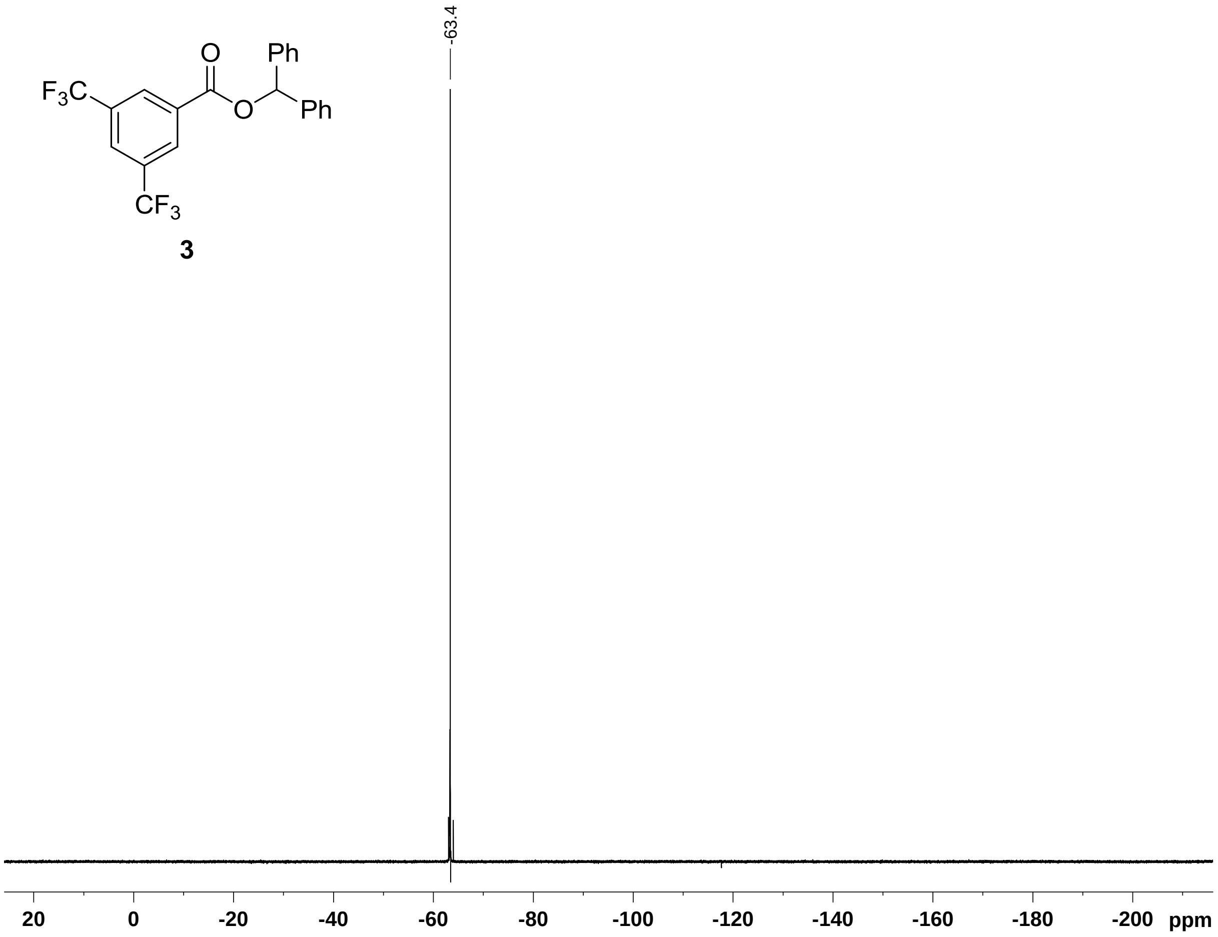
O
Ph
F3C
O
Ph
CF3
3
-63.4
20
0
-20
-40
-60
-80
-100
-120
-140
-160
-180
-200
ppm

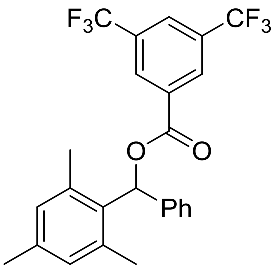
F3C
CF3
O
O
Ph

3b

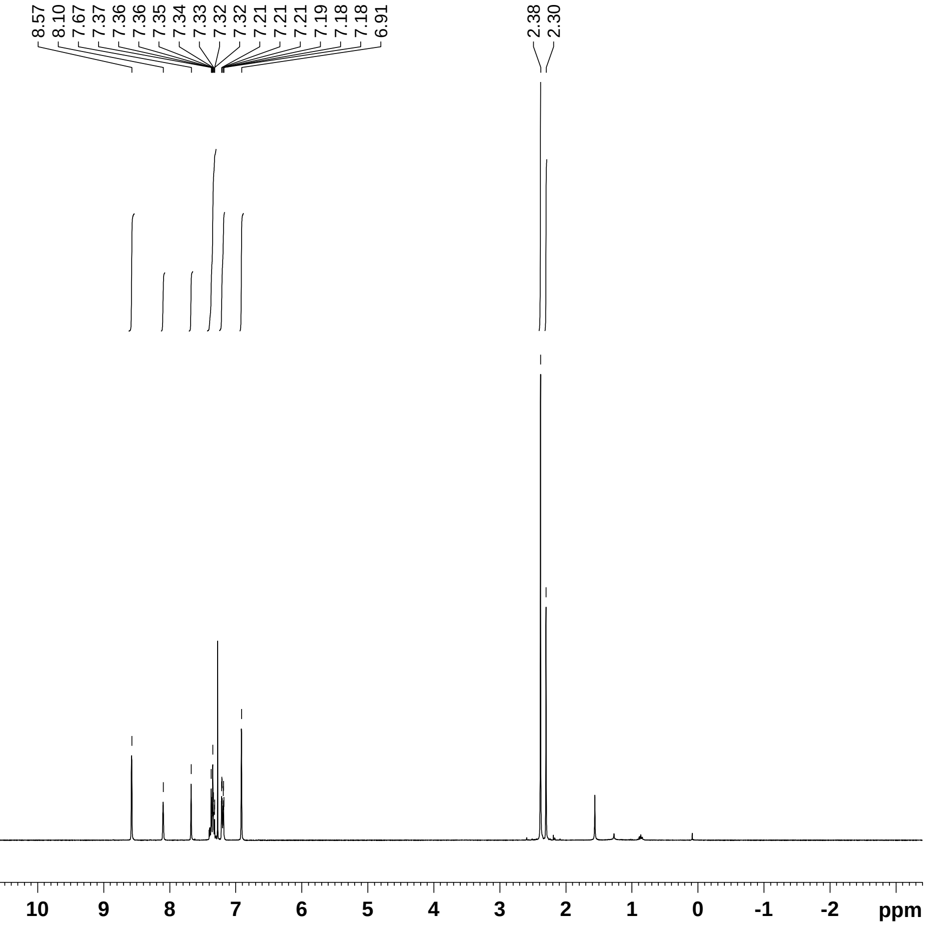
8.57
8.10
7.67
7.37
7.36
7.36
7.35
7.34
7.33
7.32
7.32
7.21
7.21
7.21
7.19
7.18
7.18
6.91
2.38
2.30
14 13 12 11 10 9 8 7 6 5 4 3 2 1 0 -1 -2 ppm

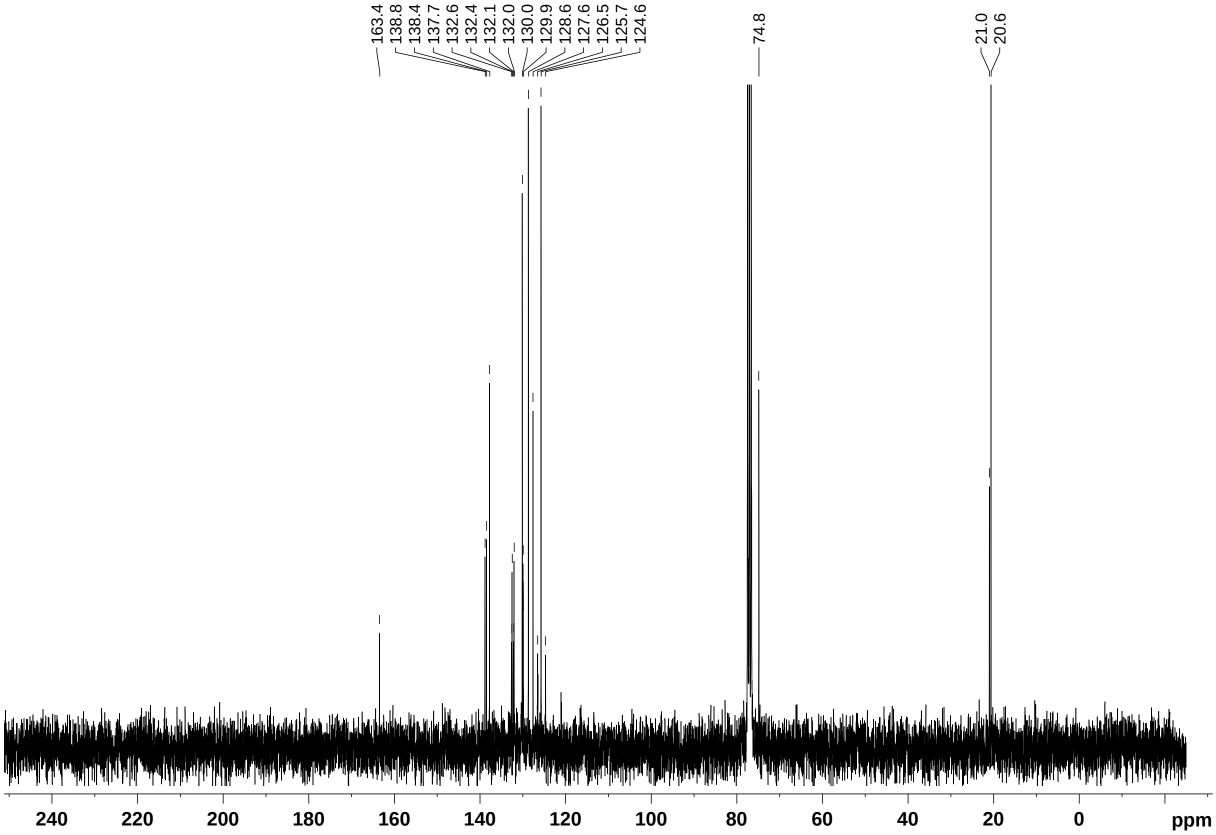
163.4
138.8
138.4
137.7
132.6
132.4
132.1
132.0
130.0
129.9
128.6
127.6
126.5
125.7
124.6
74.8
21.0
20.6
240 220 200 180 160 140 120 100 80 60 40 20 0 ppm

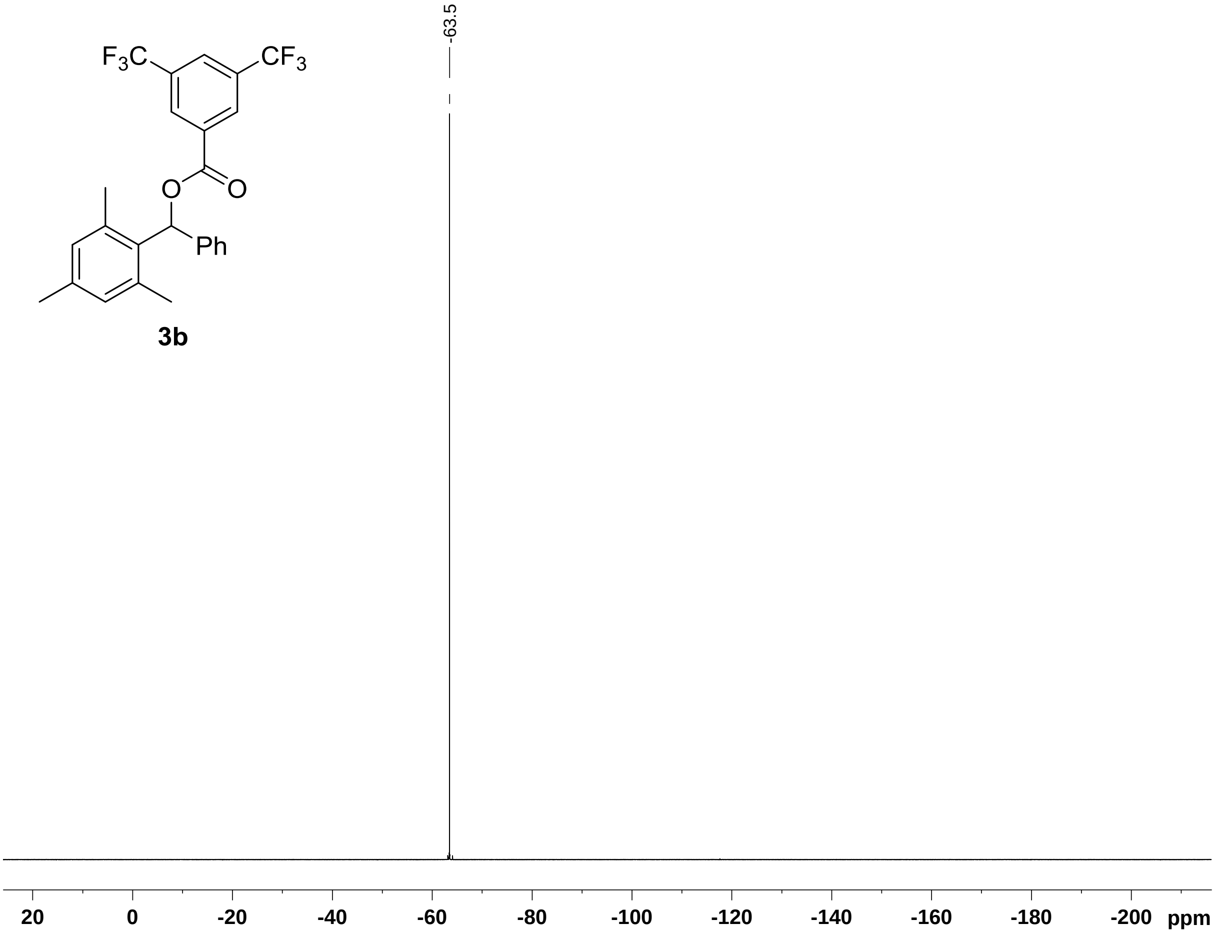
F3C
CF3
O
O
Ph
3b
-63.5
20
0
-20
-40
-60
-80
-100
-120
-140
-160
-180
-200
ppm

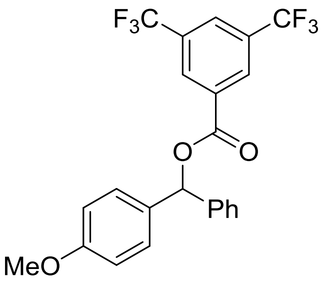
F3C
CF3
O
O
Ph
MeO

3c

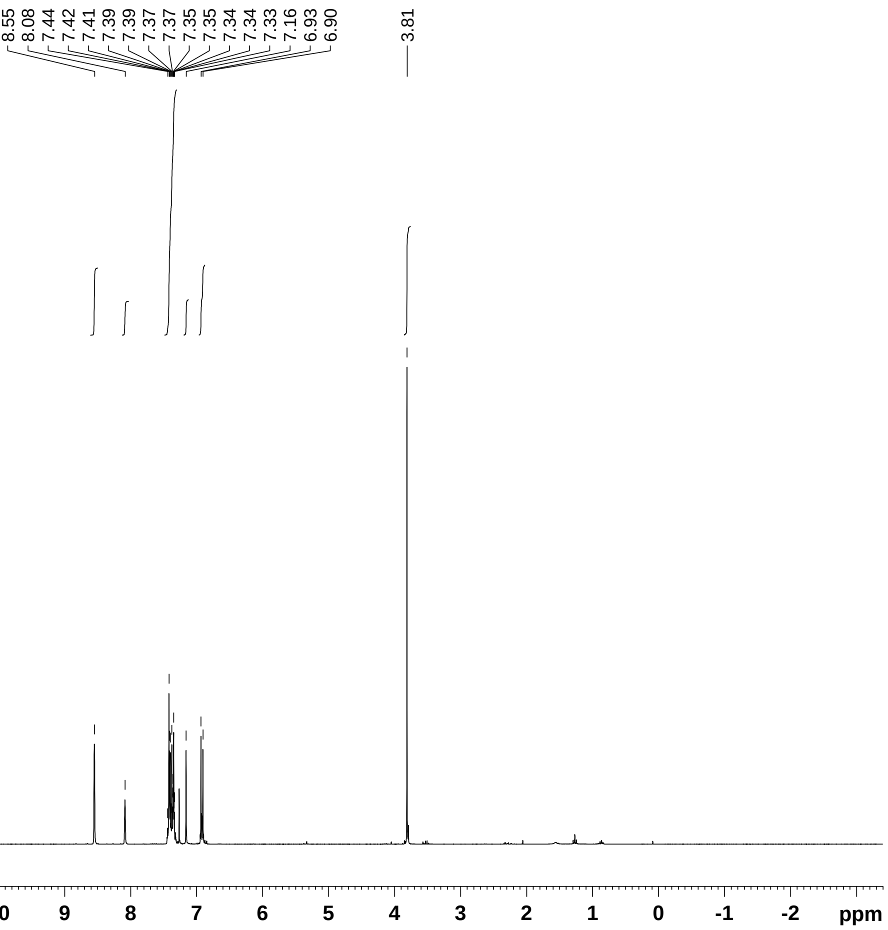
8.55
8.08
7.44
7.42
7.41
7.39
7.39
7.37
7.37
7.35
7.35
7.34
7.34
7.33
7.16
6.93
6.90
3.81
14 13 12 11 10 9 8 7 6 5 4 3 2 1 0 -1 -2 ppm

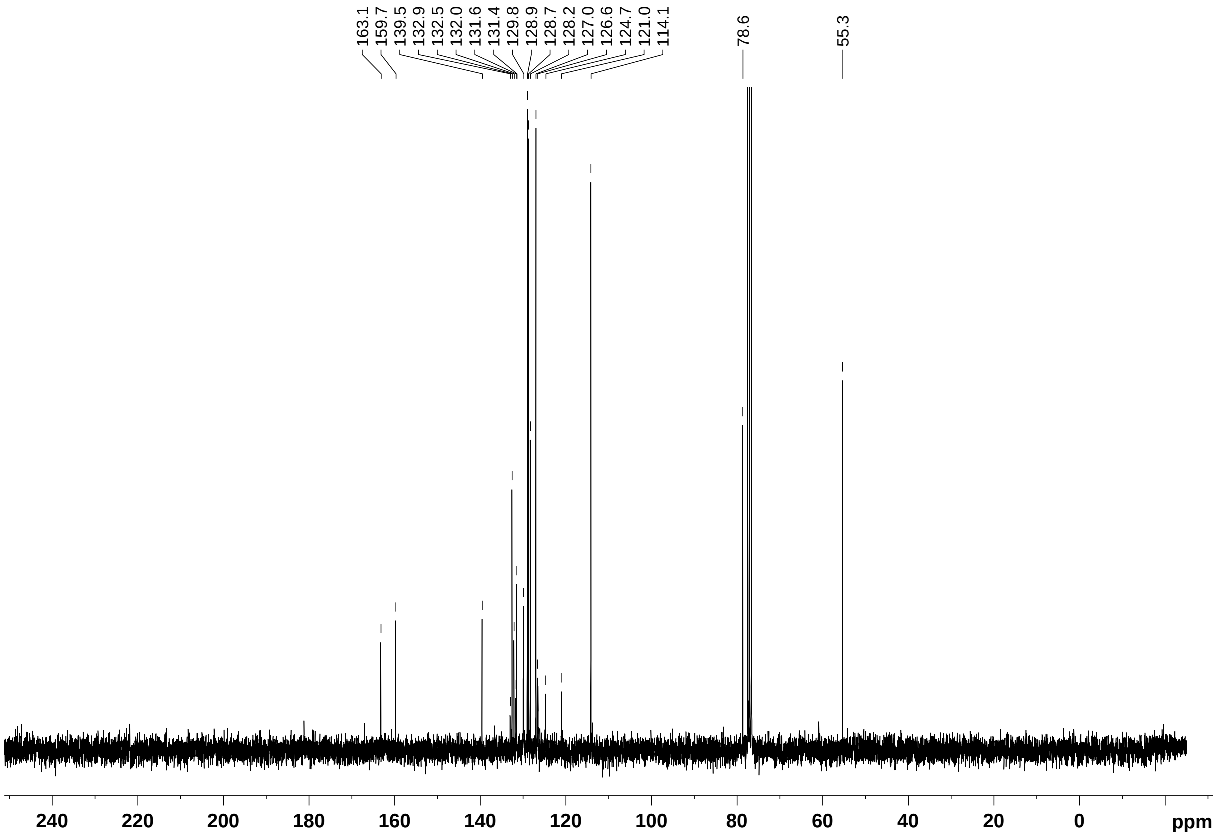
163.1
159.7
139.5
132.9
132.5
132.0
131.6
131.4
129.8
128.9
128.7
128.2
127.0
126.6
124.7
121.0
114.1
78.6
55.3
240 220 200 180 160 140 120 100 80 60 40 20 0 ppm

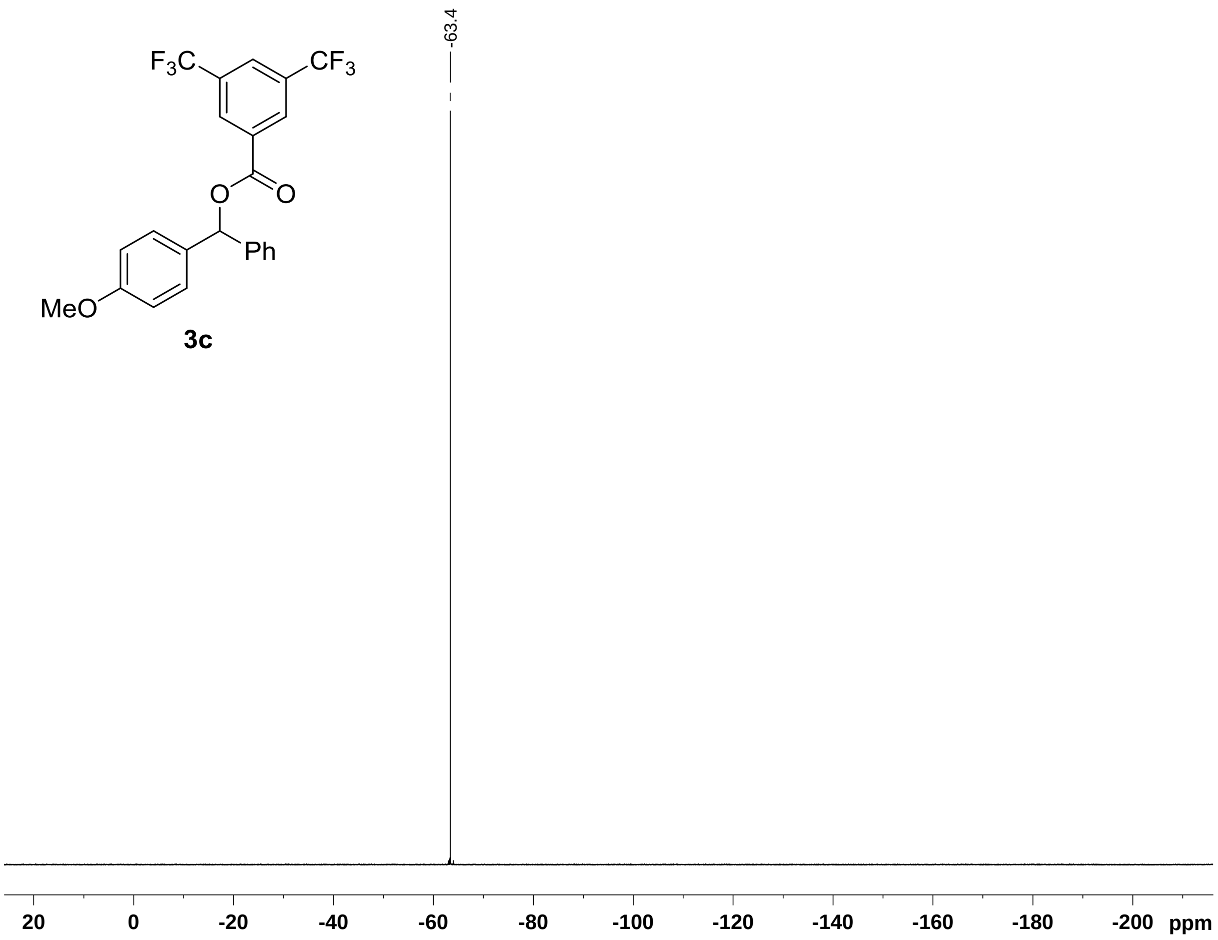

F3C
CF3
O
O
Ph
MeO
3c
-63.4
20
0
-20
-40
-60
-80
-100
-120
-140
-160
-180
-200
ppm

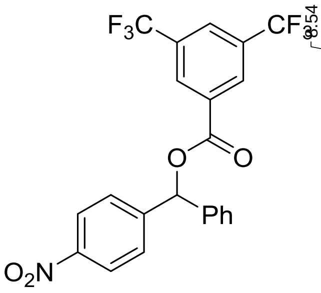
F3C
CF3
O
O
Ph
O2N

3d

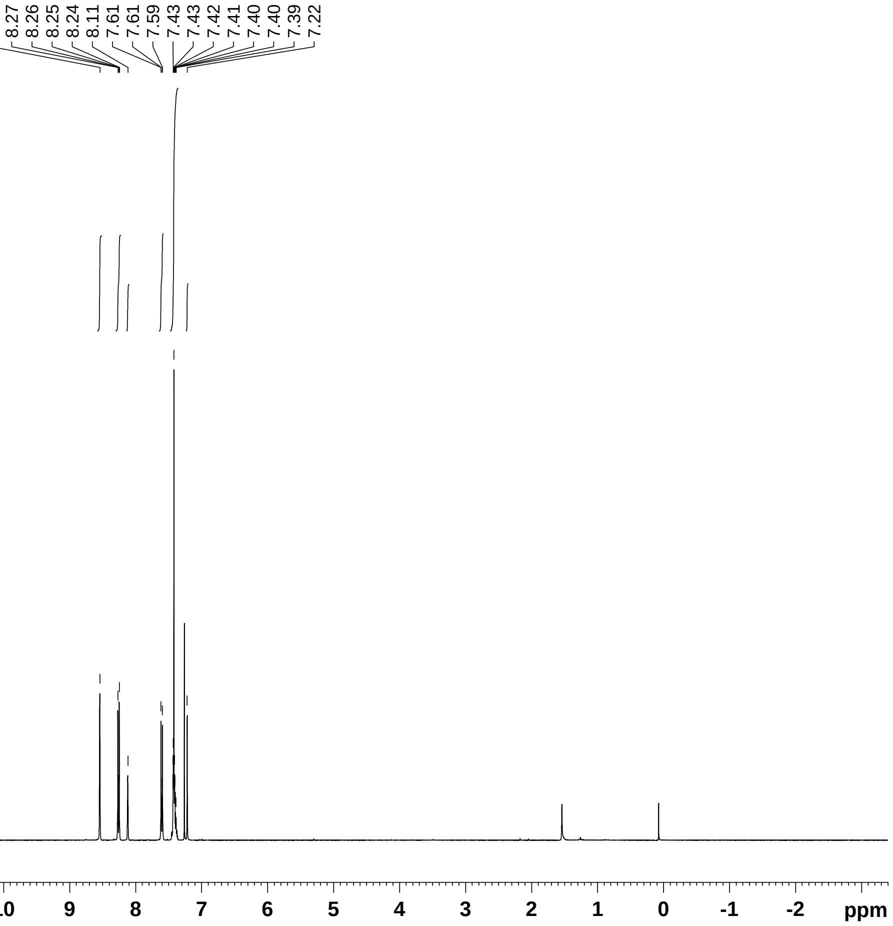
8.54
8.27
8.26
8.25
8.24
8.11
7.61
7.61
7.59
7.43
7.43
7.42
7.41
7.40
7.40
7.39
7.22
14 13 12 11 10 9 8 7 6 5 4 3 2 1 0 -1 -2 ppm

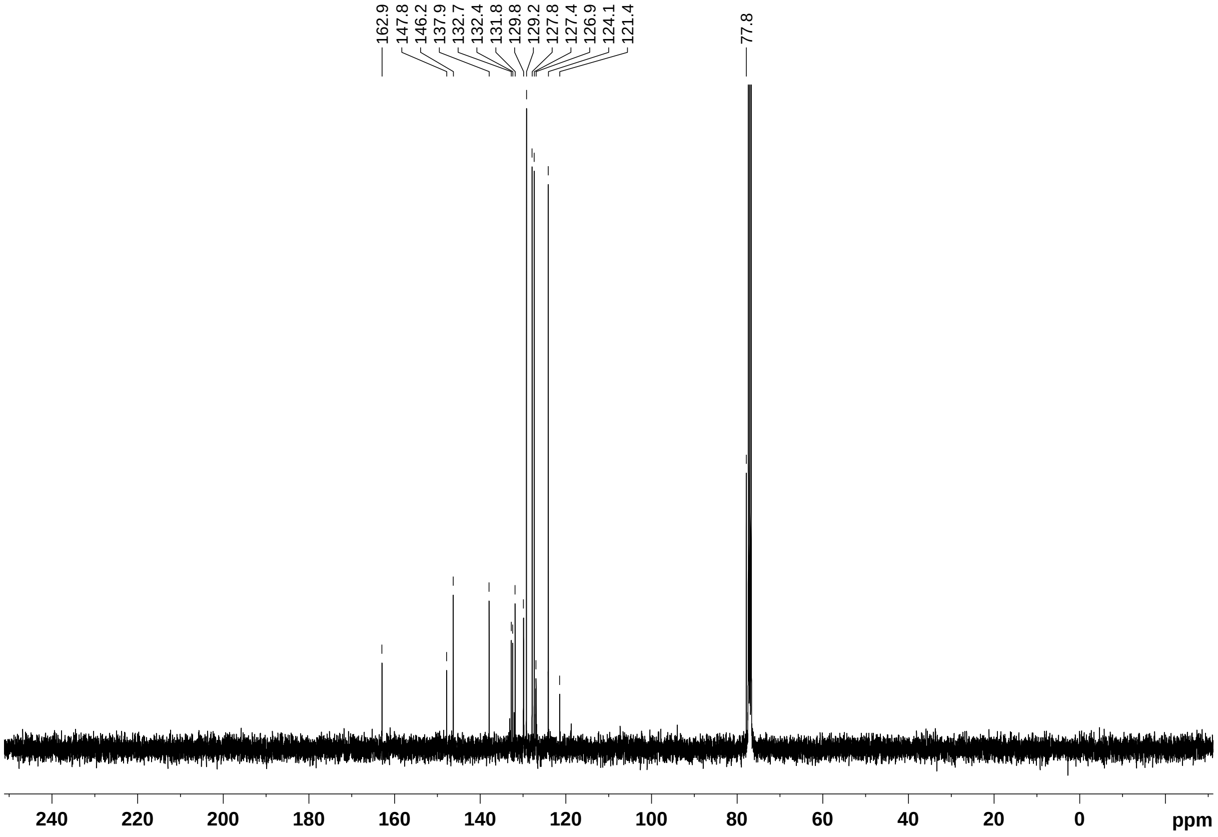
162.9
147.8
146.2
137.9
132.7
132.4
131.8
129.8
129.2
127.8
127.4
126.9
124.1
121.4
77.8
240 220 200 180 160 140 120 100 80 60 40 20 0 ppm

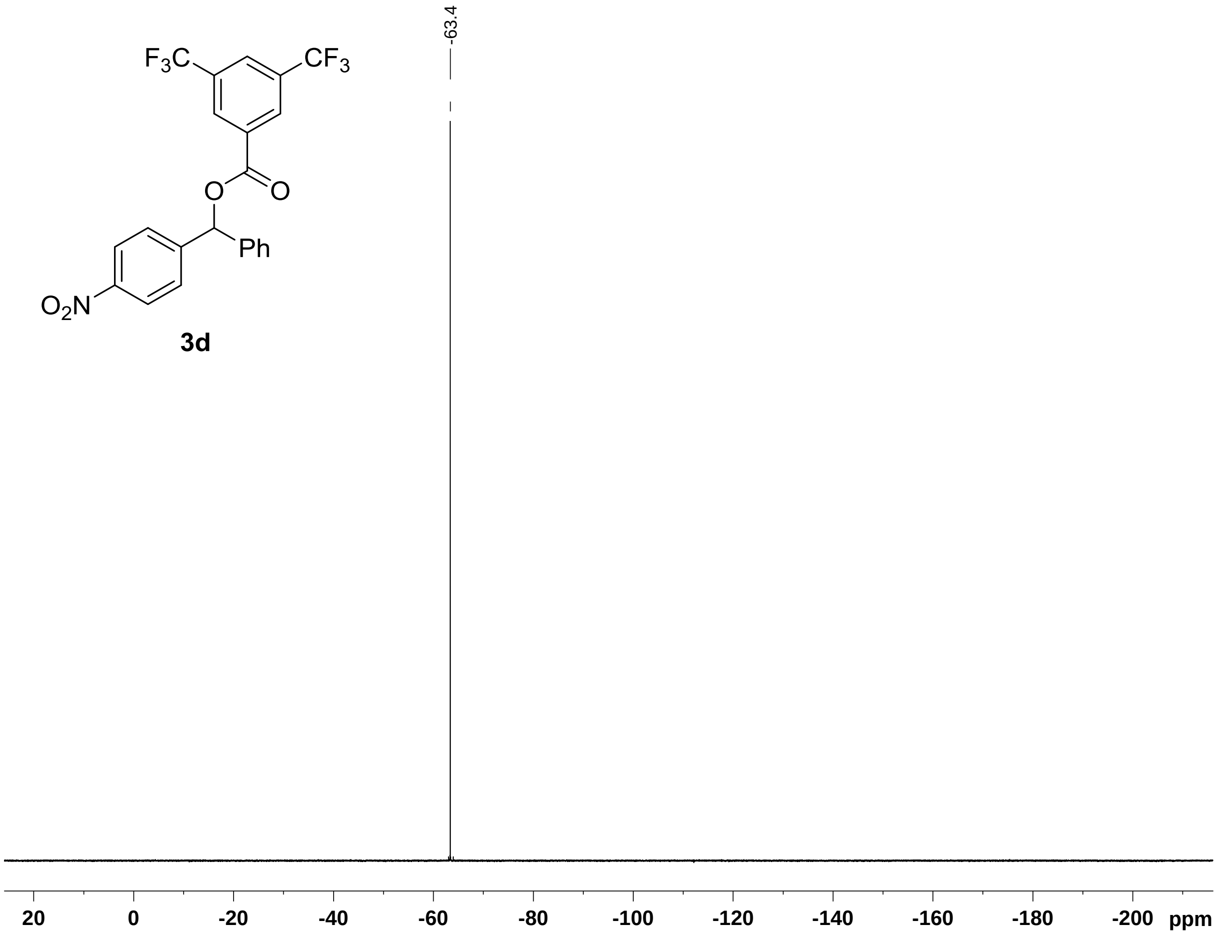
F3C
CF3
O
O
Ph
O2N
3d
-63.4
20 0 -20 -40 -60 -80 -100 -120 -140 -160 -180 -200 ppm

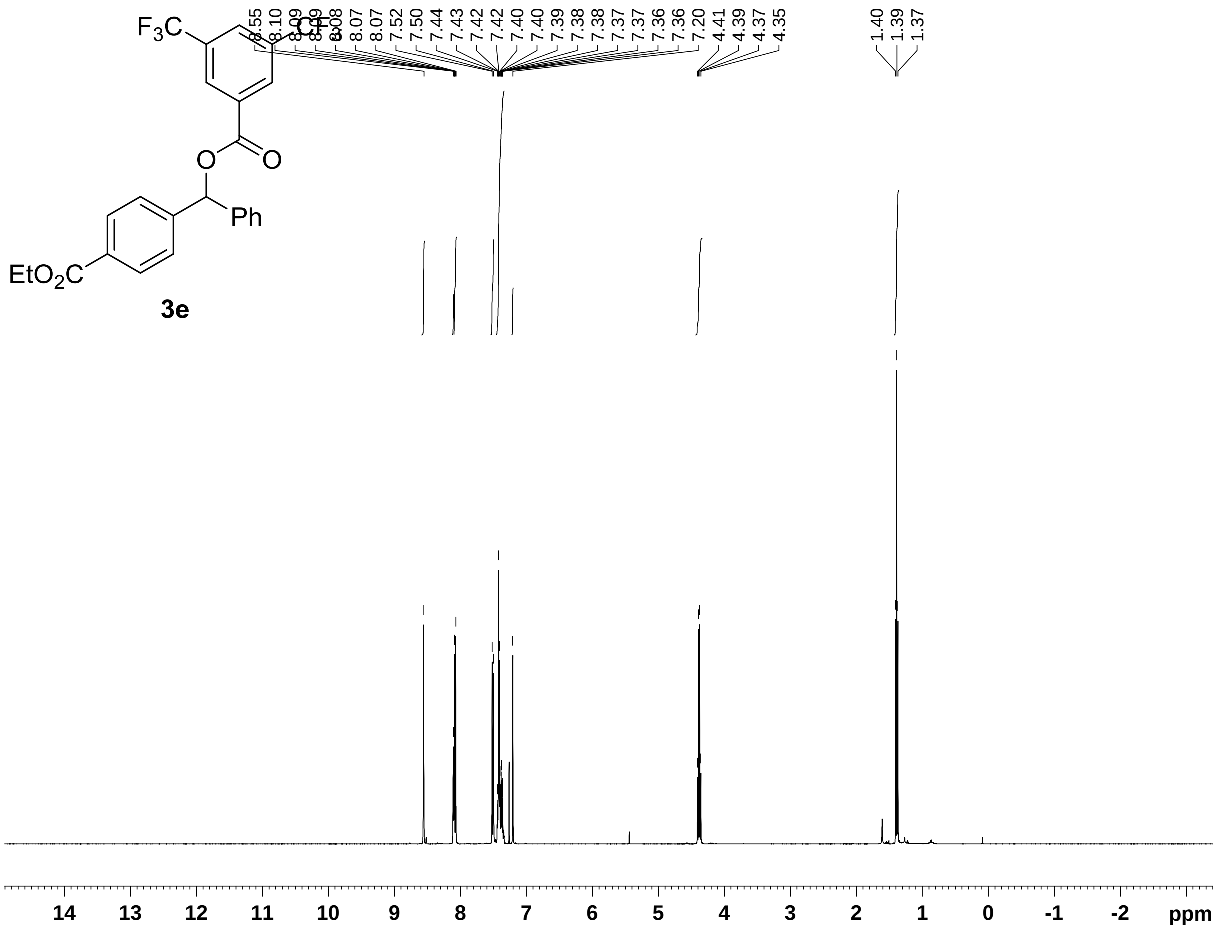
F3C
O
O
Ph
EtO2C
3e
8.55 8.10 8.09 8.09 8.08 8.07 7.52 7.50 7.44 7.43 7.42 7.42 7.40 7.40 7.39 7.38 7.38 7.37 7.37 7.36 7.36 7.20 4.41 4.39 4.37 4.35
1.40 1.39 1.37
14 13 12 11 10 9 8 7 6 5 4 3 2 1 0 -1 -2 ppm

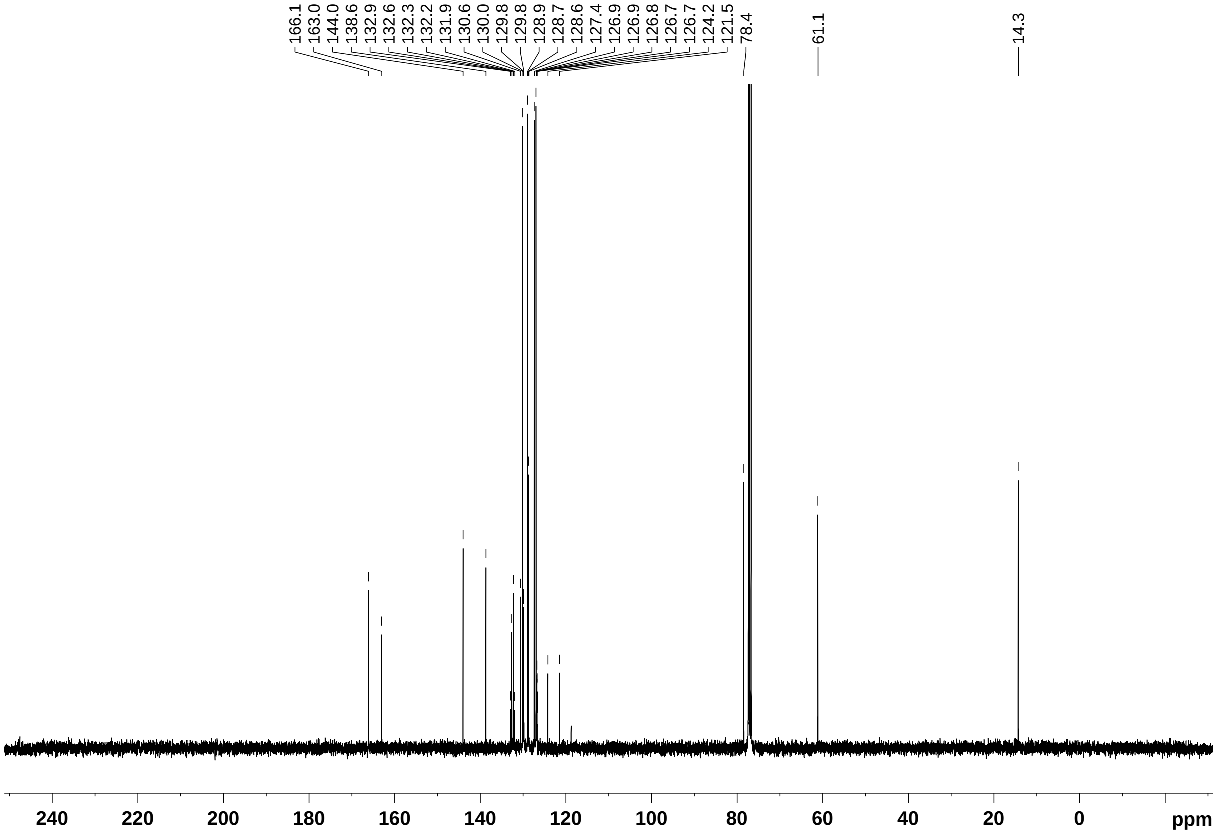
166.1 163.0 144.0 138.6 132.9 132.6 132.3 132.2 131.9 130.6 130.0 129.8 129.8 128.9 128.7 128.6 127.4 126.9 126.9 126.8 126.7 126.7 124.2 121.5 78.4
61.1
14.3
240 220 200 180 160 140 120 100 80 60 40 20 0 ppm

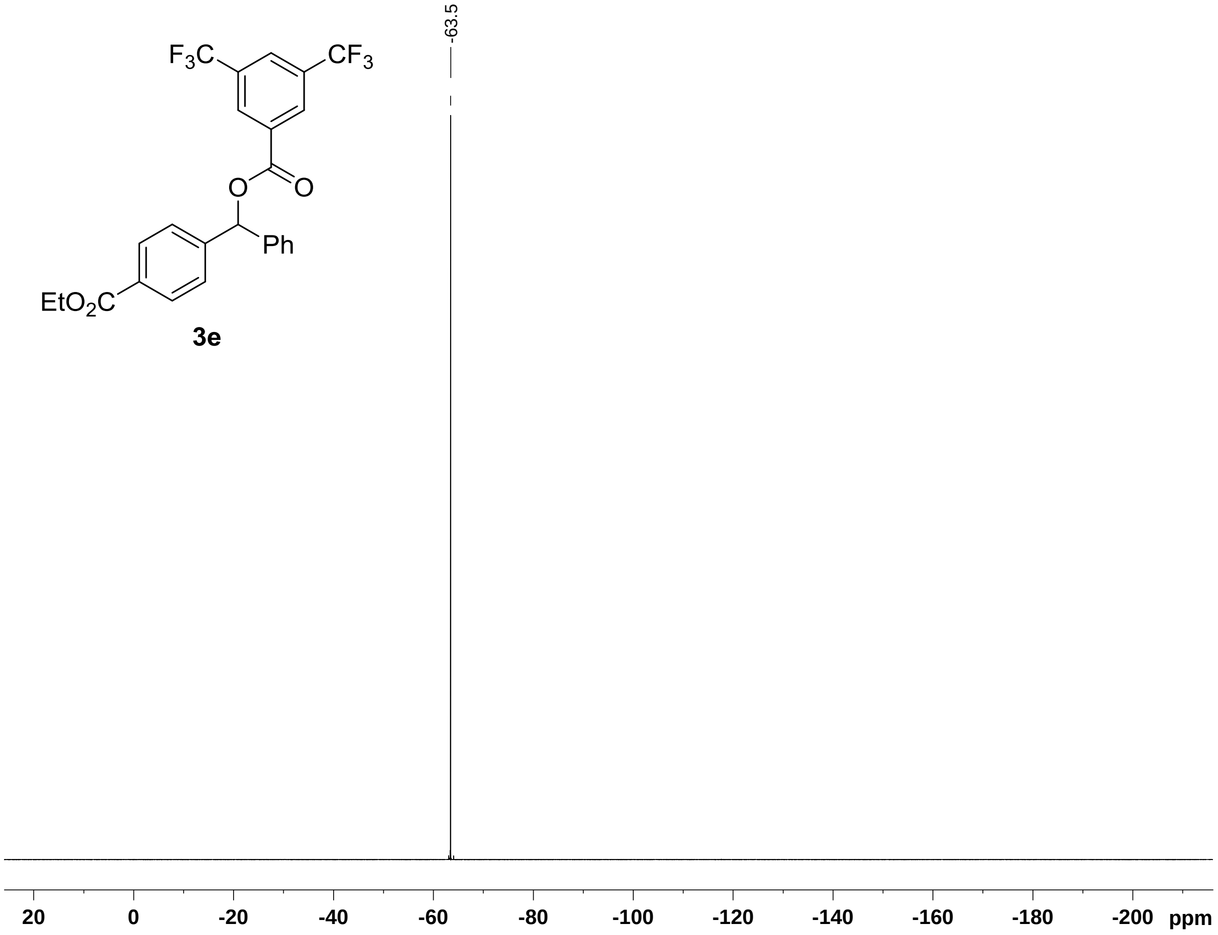
F3C
CF3
O
O
Ph
EtO2C
-63.5
20
0
-20
-40
-60
-80
-100
-120
-140
-160
-180
-200
ppm

3e

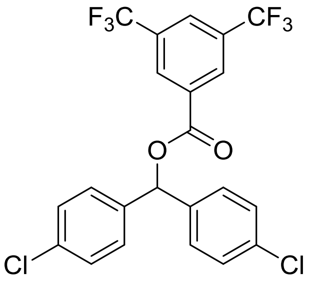

F3C
CF3
O
O
Cl
Cl

3f

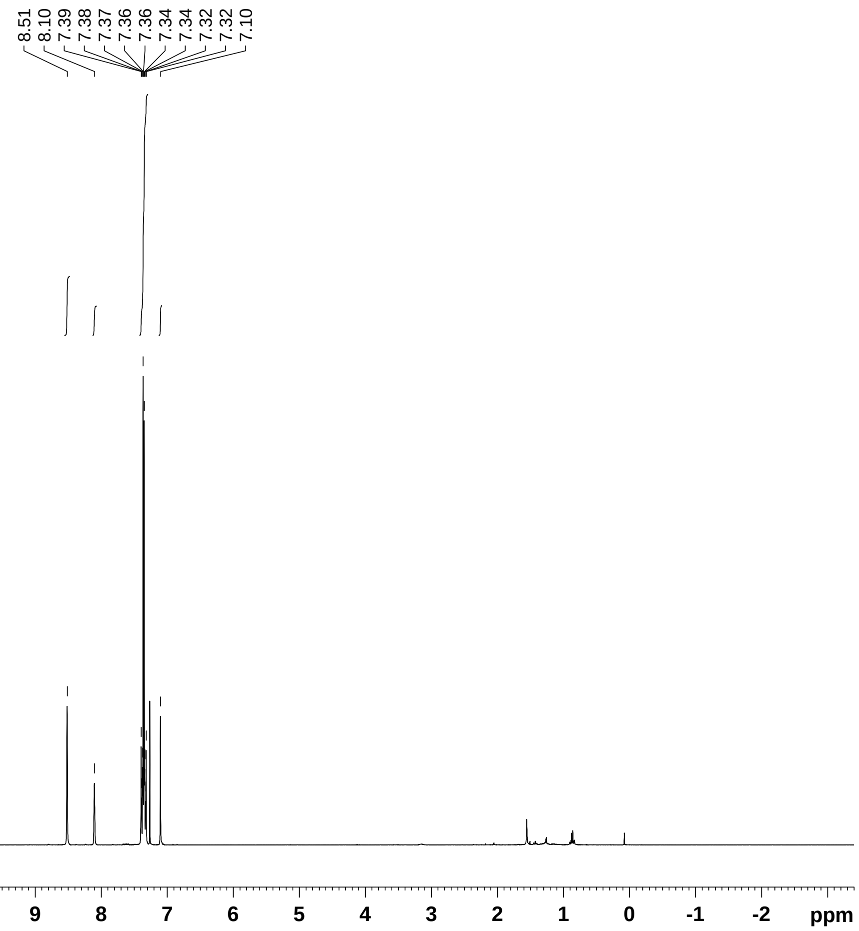

8.51
8.10
7.39
7.38
7.37
7.36
7.36
7.34
7.34
7.32
7.32
7.10
14 13 12 11 10 9 8 7 6 5 4 3 2 1 0 -1 -2 ppm

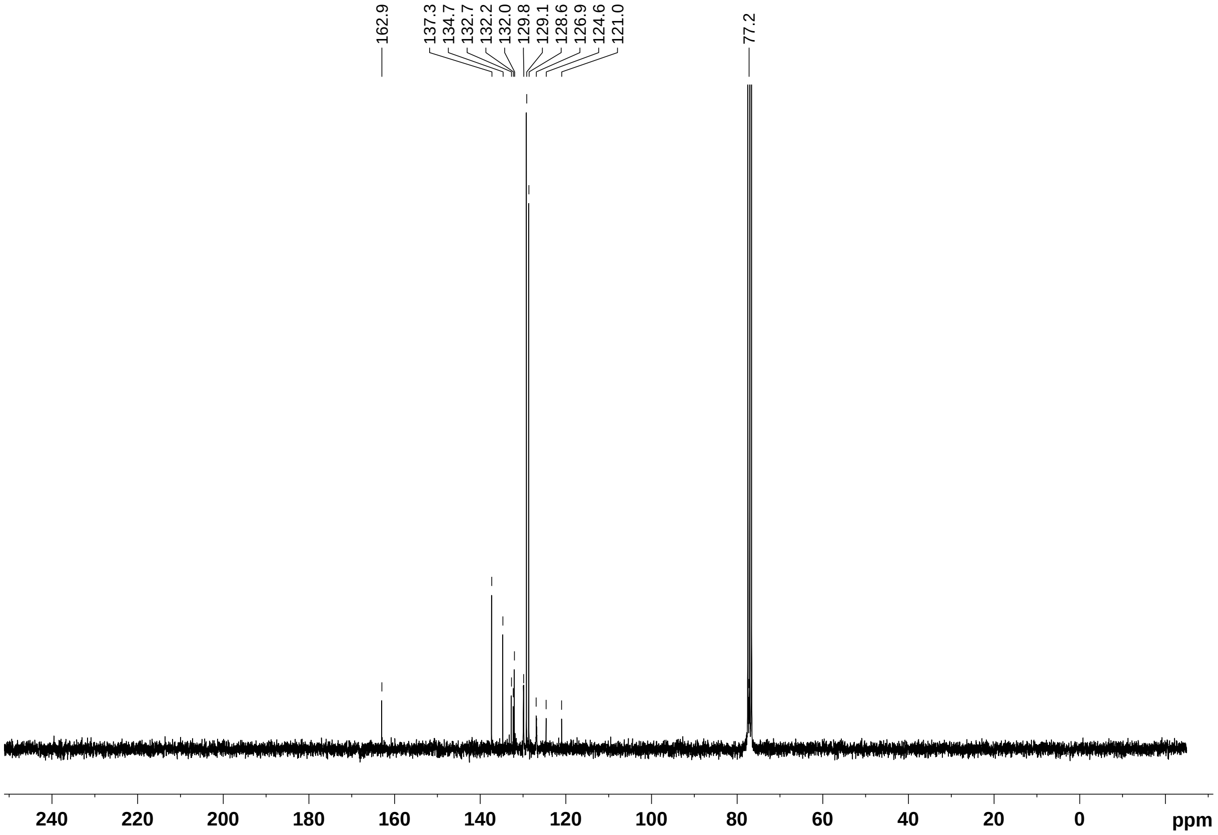

162.9
137.3
134.7
132.7
132.2
132.0
129.8
129.1
128.6
126.9
124.6
121.0
77.2
240 220 200 180 160 140 120 100 80 60 40 20 0 ppm

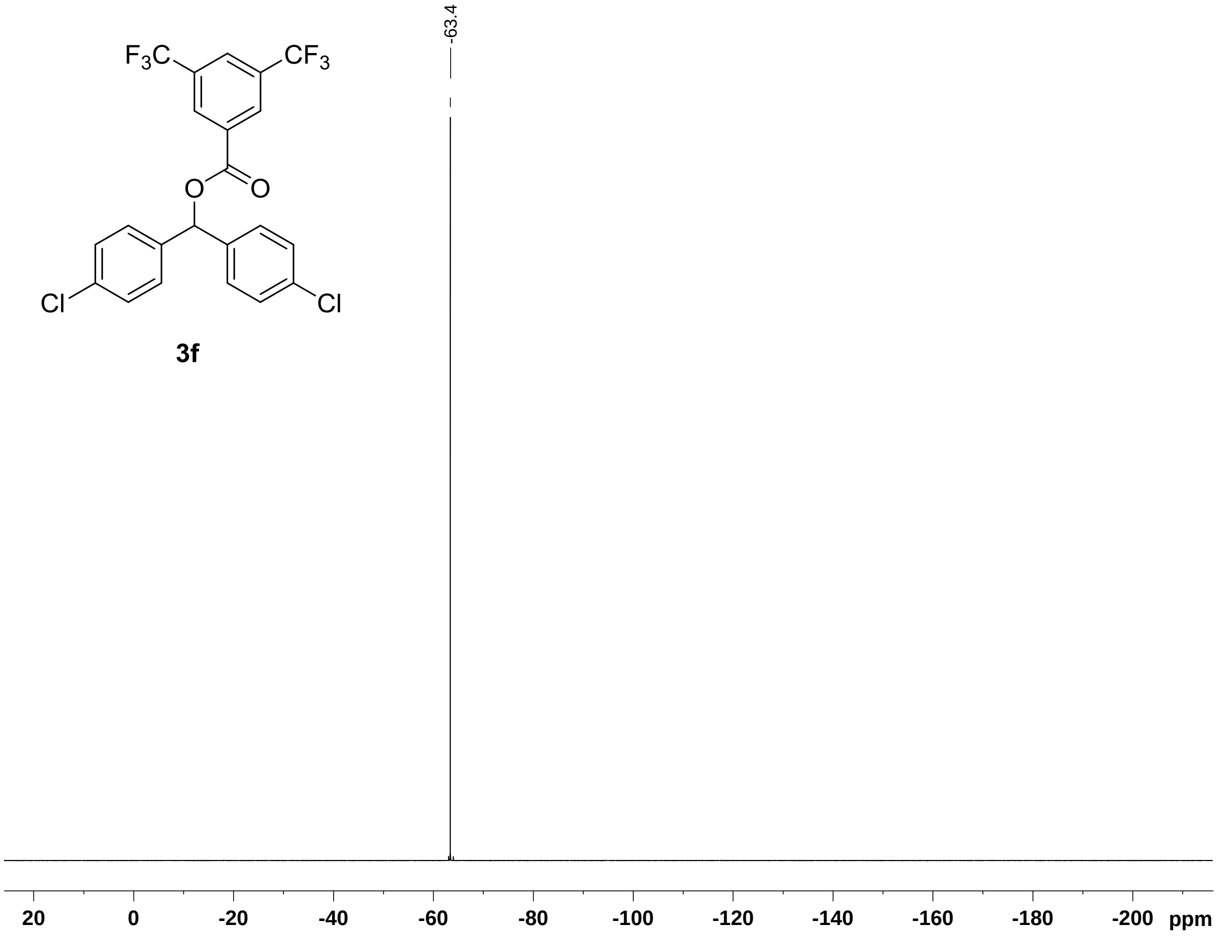

F3C
CF3
O
O
Cl
Cl
3f
-63.4
20
0
-20
-40
-60
-80
-100
-120
-140
-160
-180
-200
ppm

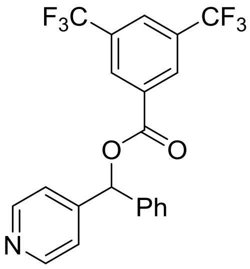
F3C
CF3
O
O
Ph
N

3g

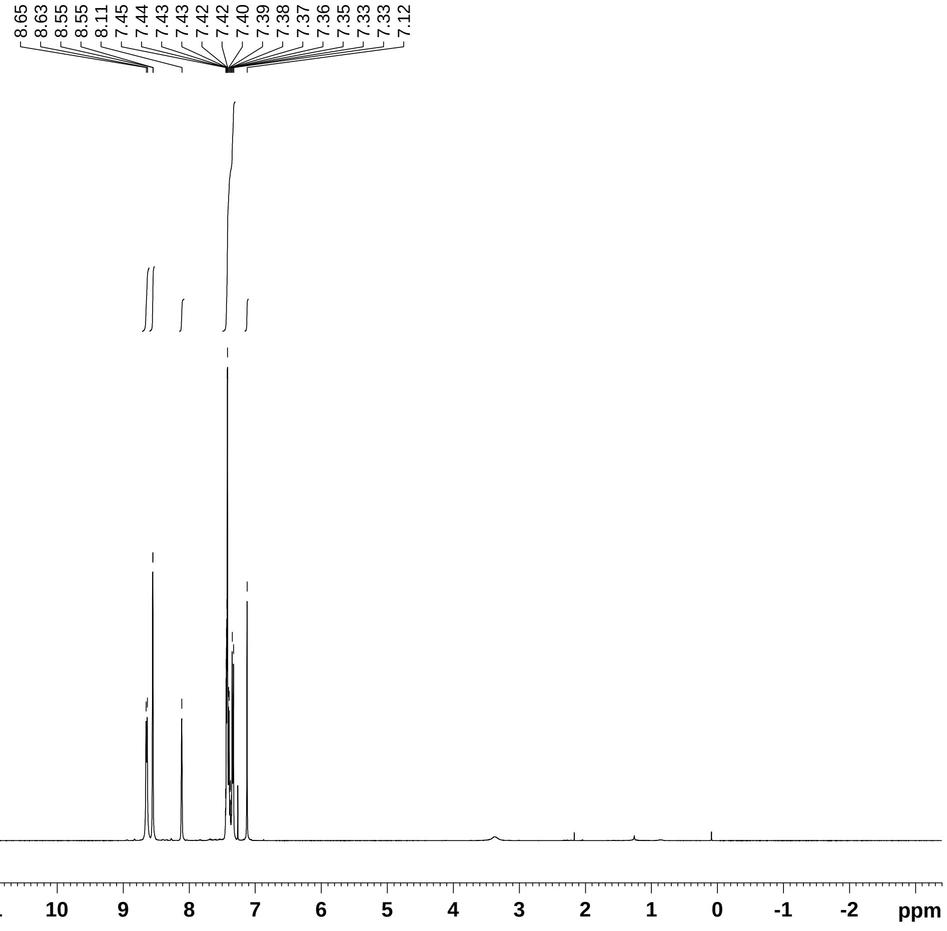
8.65
8.63
8.55
8.55
8.11
7.45
7.44
7.43
7.43
7.42
7.42
7.40
7.39
7.38
7.37
7.36
7.35
7.33
7.33
7.12
14
13
12
11
10
9
8
7
6
5
4
3
2
1
0
-1
-2
ppm

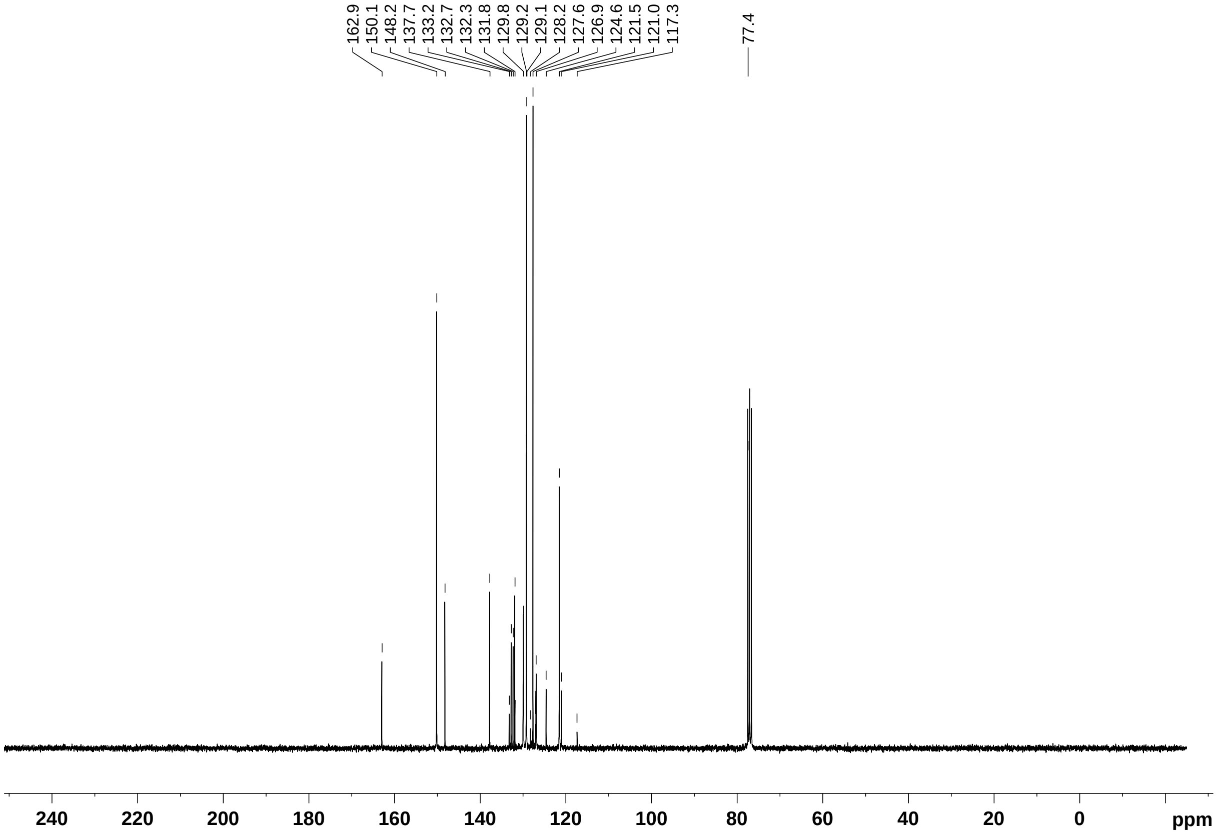
162.9
150.1
148.2
137.7
133.2
132.7
132.3
131.8
129.8
129.2
129.1
128.2
127.6
126.9
124.6
121.5
121.0
117.3
77.4
240
220
200
180
160
140
120
100
80
60
40
20
0
ppm

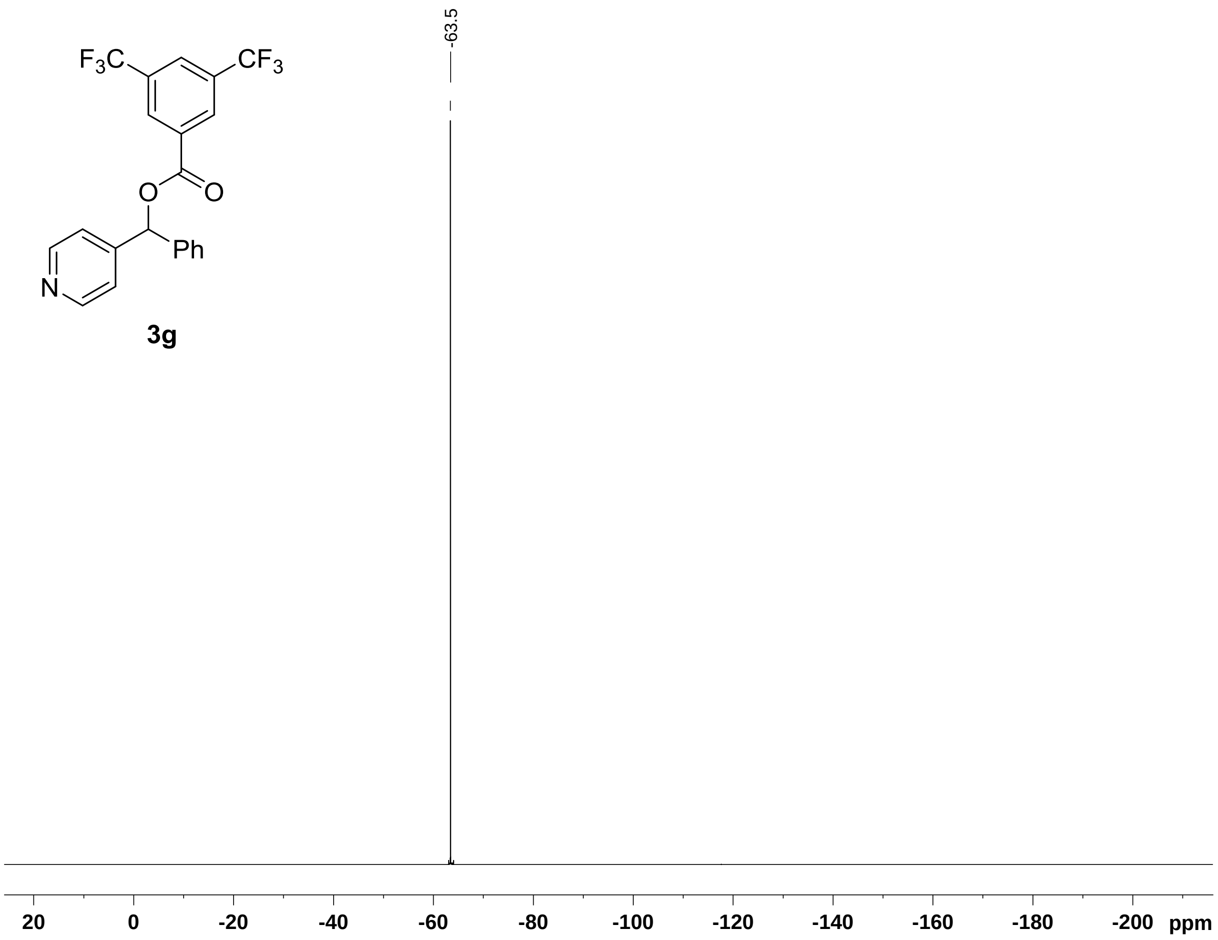

3g

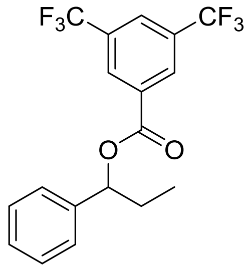
F3C
CF3
O
O

3h

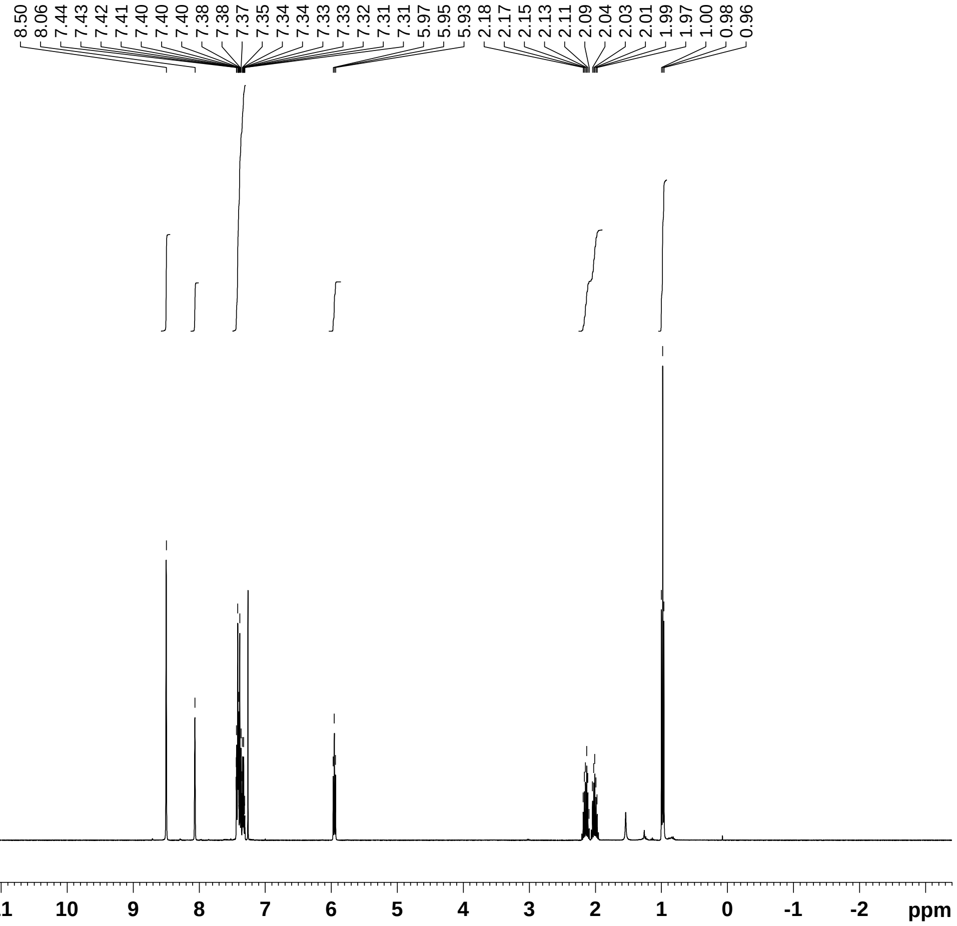
8.50 8.06 7.44 7.43 7.42 7.41 7.40 7.40 7.38 7.38 7.37 7.35 7.34 7.34 7.33 7.33 7.32 7.31 7.31 5.97 5.95 5.93 2.18 2.17 2.15 2.13 2.11 2.09 2.04 2.03 2.01 1.99 1.97 1.00 0.98 0.96
14 13 12 11 10 9 8 7 6 5 4 3 2 1 0 -1 -2 ppm

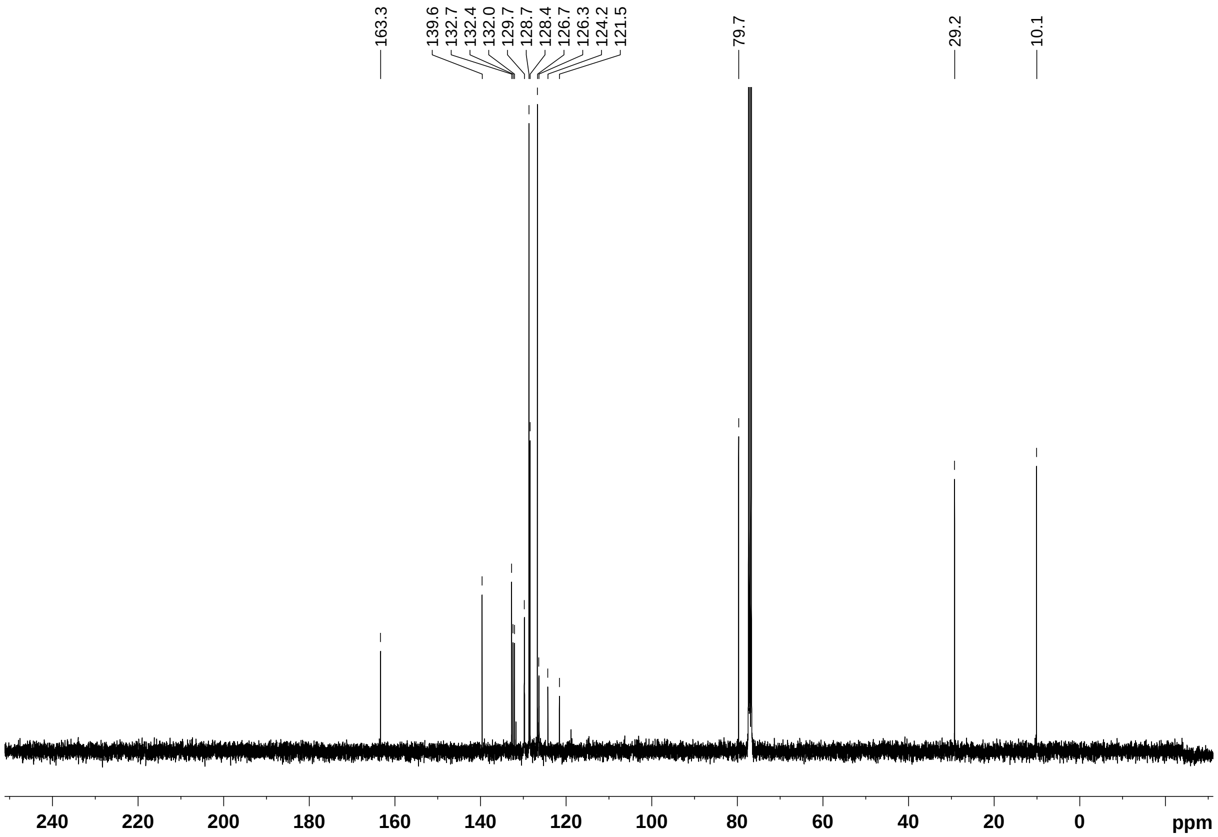
163.3
139.6 132.7 132.4 132.0 129.7 128.7 128.4 126.7 126.3 124.2 121.5
79.7
29.2
10.1
240 220 200 180 160 140 120 100 80 60 40 20 0 ppm

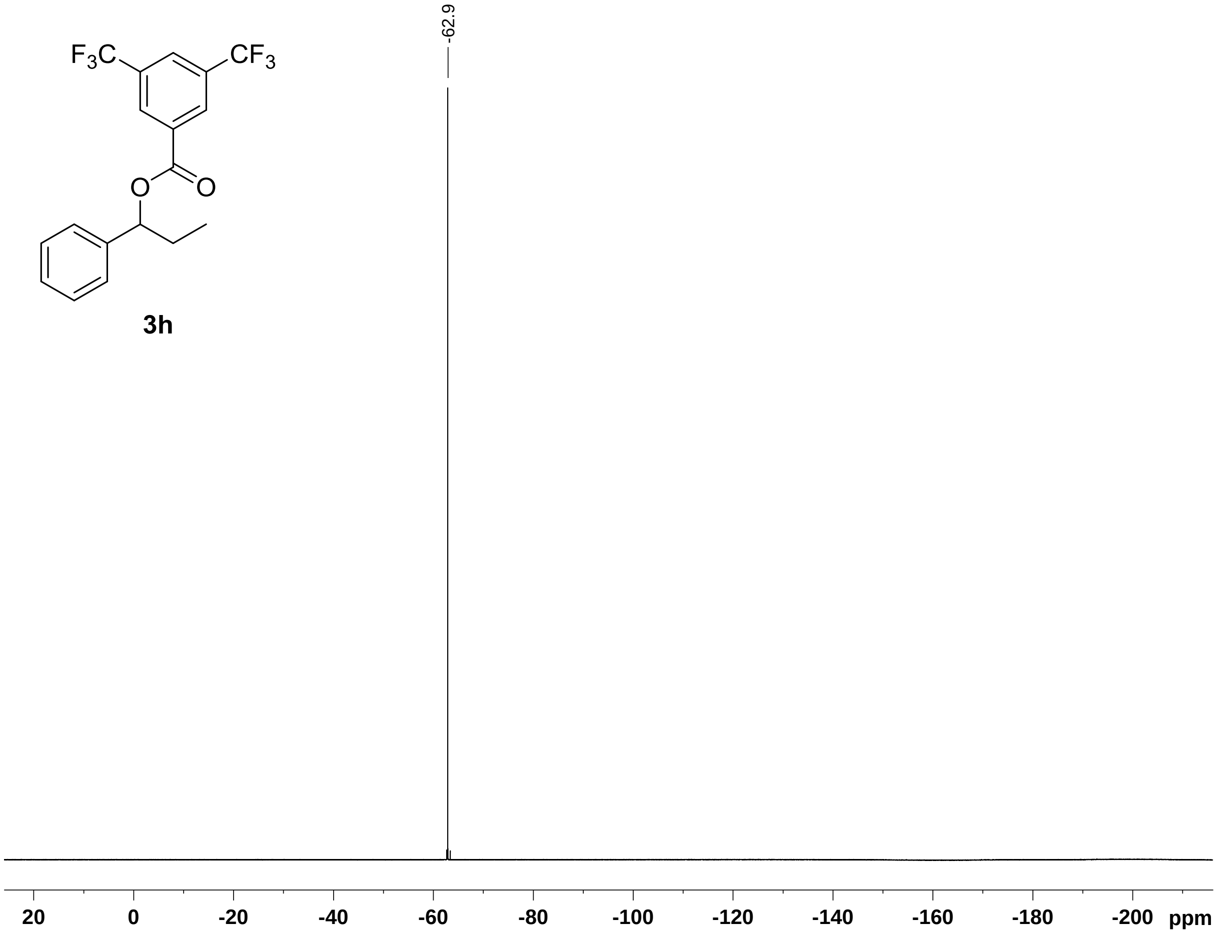
F3C
CF3
O
O
3h
-62.9
20
0
-20
-40
-60
-80
-100
-120
-140
-160
-180
-200
ppm

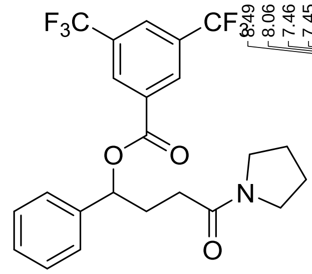
F$_3$C
CF$_3$
O
O
N
O

3i

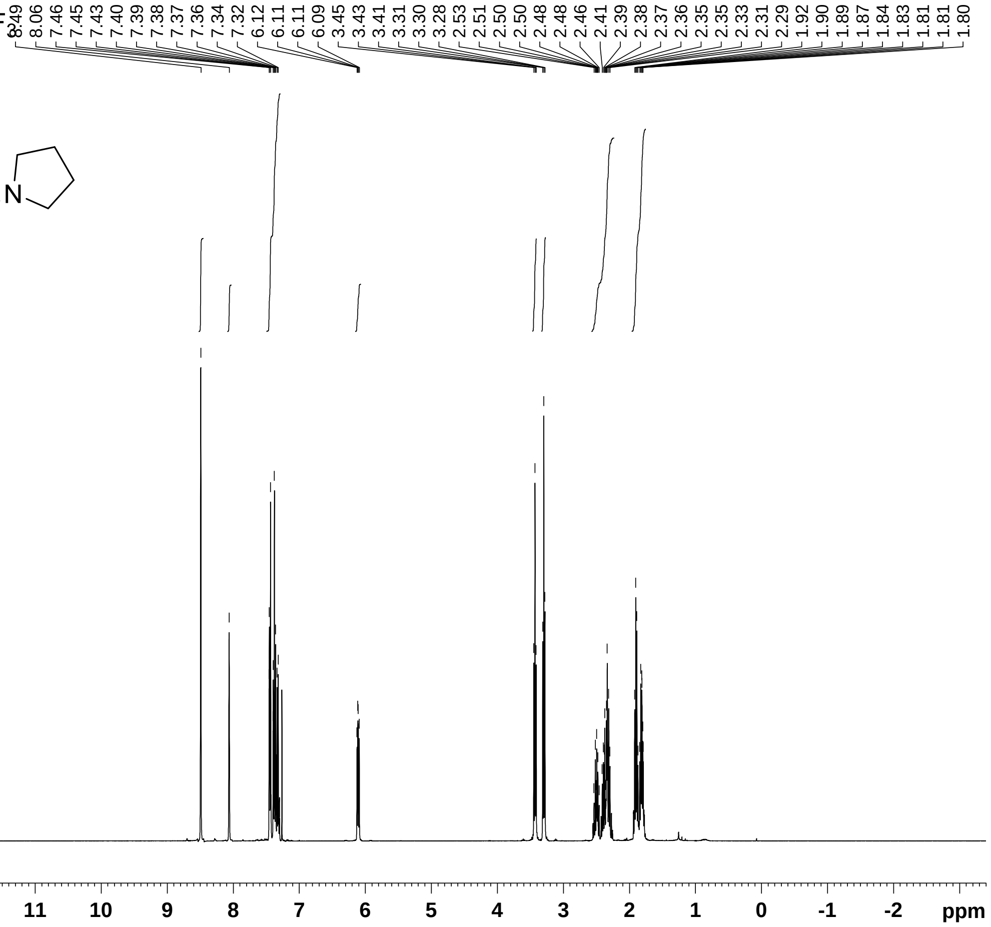
8.49 8.06 7.46 7.45 7.43 7.40 7.39 7.38 7.37 7.36 7.34 7.32 6.12 6.11 6.11 6.09 3.45 3.43 3.41 3.31 3.30 3.28 2.53 2.51 2.50 2.50 2.48 2.48 2.46 2.41 2.39 2.38 2.37 2.36 2.35 2.35 2.33 2.31 2.29 1.92 1.90 1.89 1.87 1.84 1.83 1.81 1.81 1.80
14 13 12 11 10 9 8 7 6 5 4 3 2 1 0 -1 -2 ppm

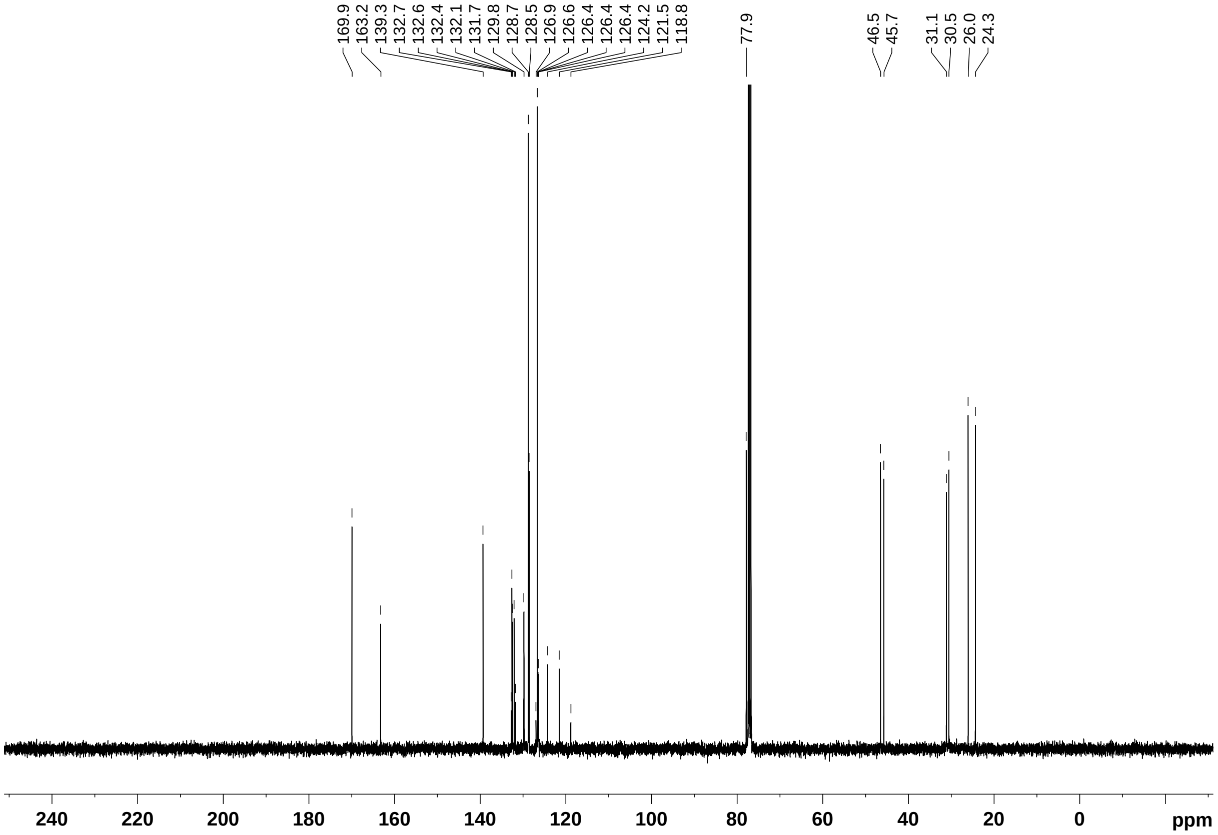
169.9 163.2 139.3 132.7 132.6 132.4 132.1 131.7 129.8 128.7 128.5 126.9 126.6 126.4 126.4 124.2 121.5 118.8
77.9
46.5 45.7
31.1 30.5 26.0 24.3
240 220 200 180 160 140 120 100 80 60 40 20 0 ppm

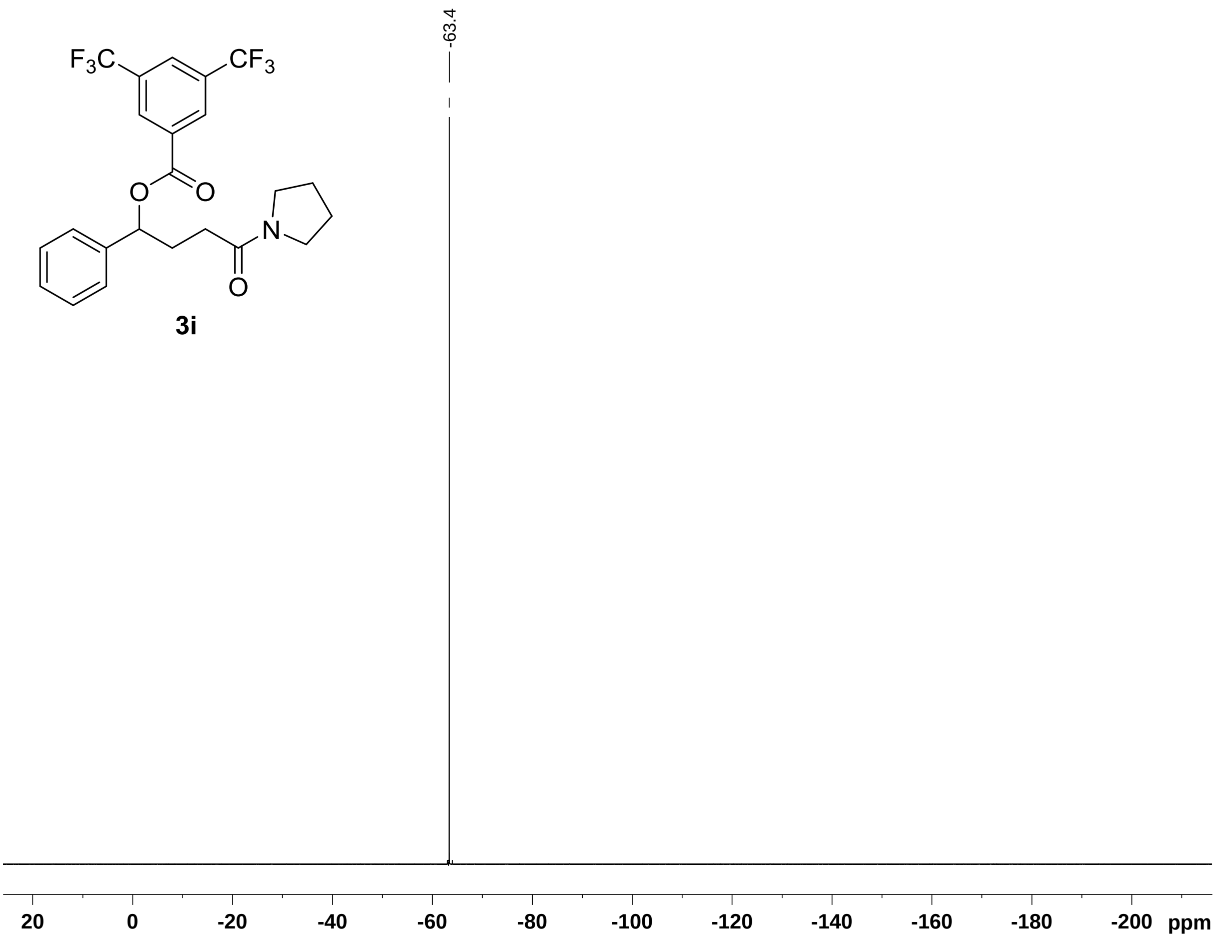
F3C
CF3
O
O
N
O
3i
-63.4
20
0
-20
-40
-60
-80
-100
-120
-140
-160
-180
-200
ppm

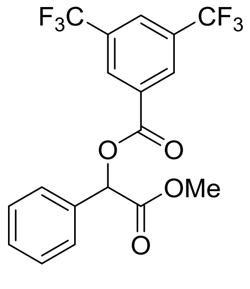
F3C
CF3
O
O
OMe
O

3j

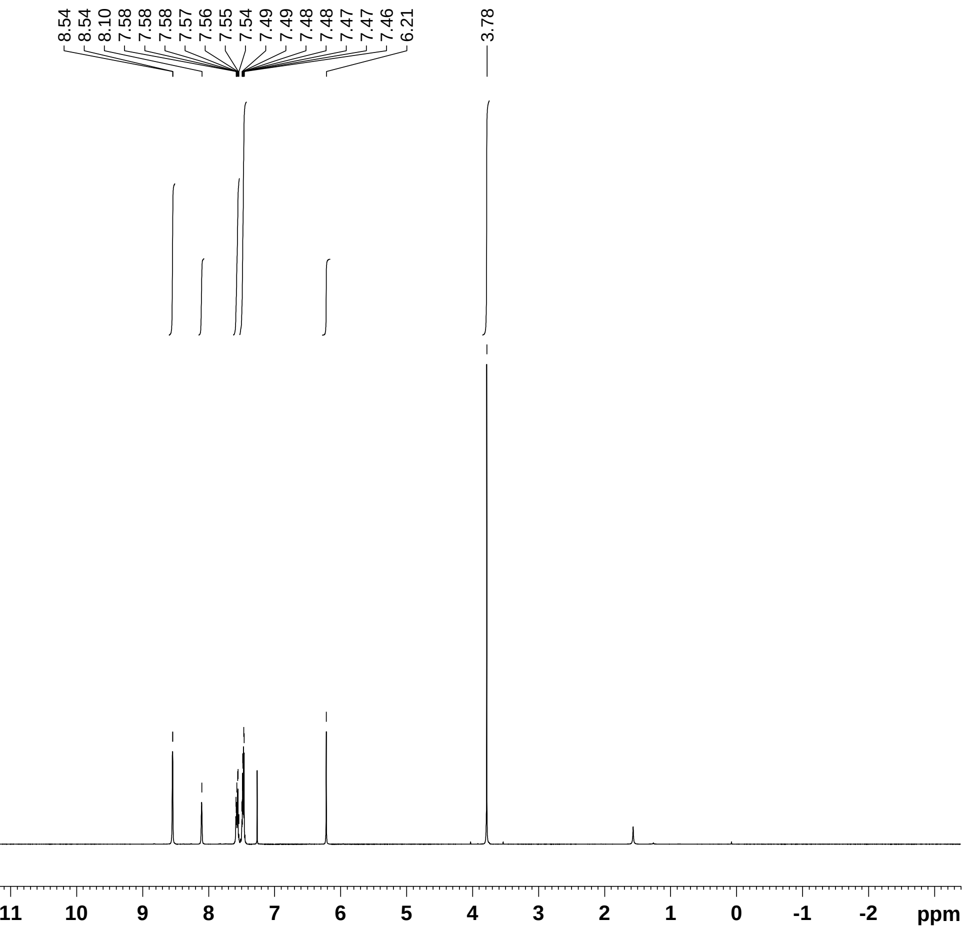
8.54
8.54
8.10
7.58
7.58
7.58
7.57
7.56
7.55
7.54
7.49
7.49
7.48
7.48
7.47
7.47
7.46
6.21
3.78
14 13 12 11 10 9 8 7 6 5 4 3 2 1 0 -1 -2 ppm

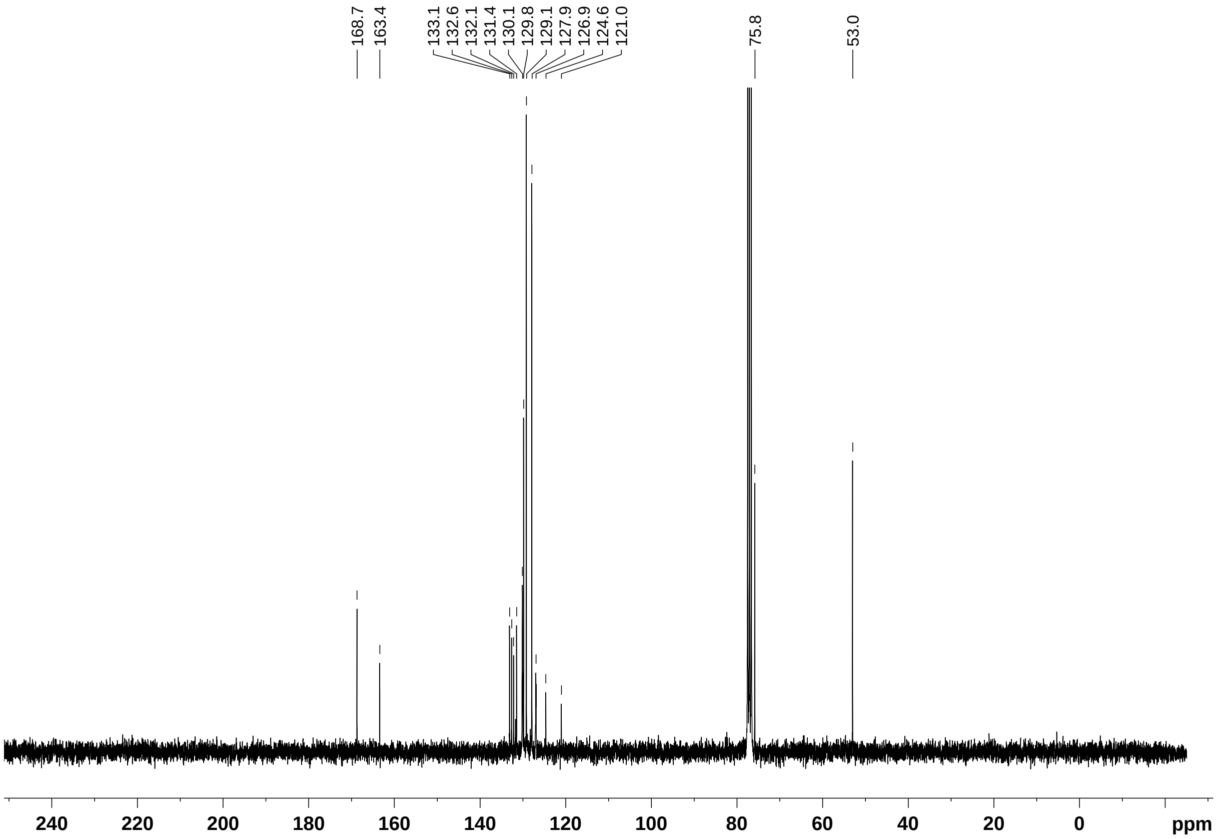
168.7
163.4
133.1
132.6
132.1
131.4
130.1
129.8
129.1
127.9
126.9
124.6
121.0
75.8
53.0
240 220 200 180 160 140 120 100 80 60 40 20 0 ppm

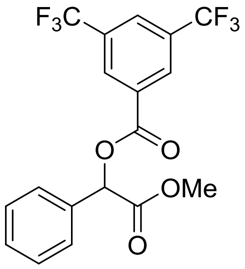

3j

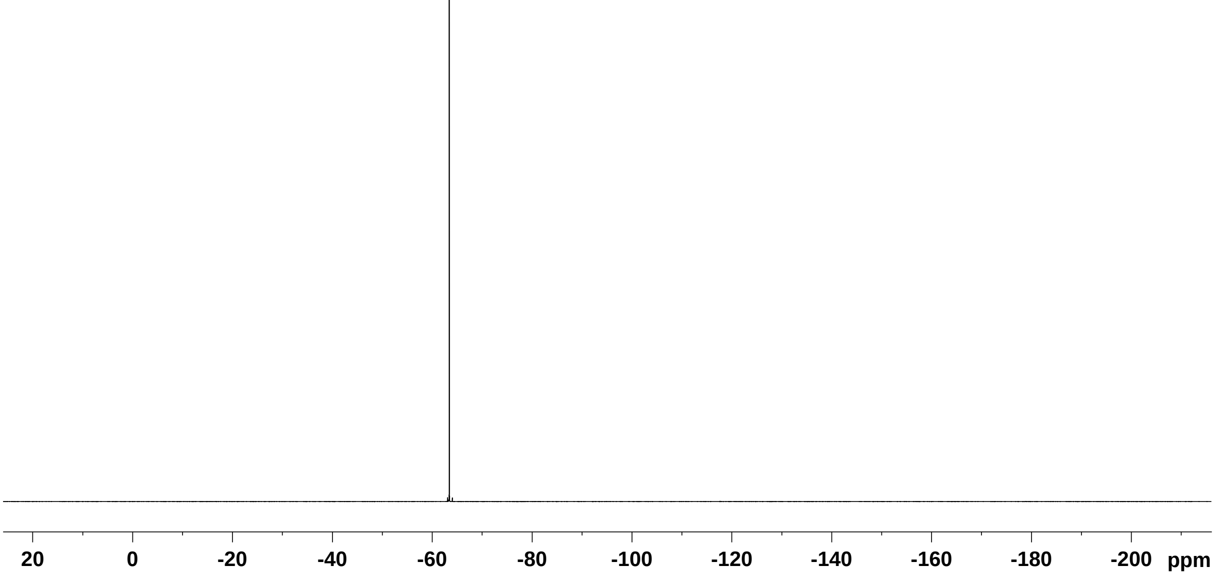

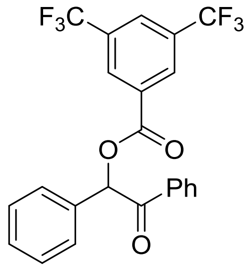
F3C
CF3
O
O
Ph
O

3k

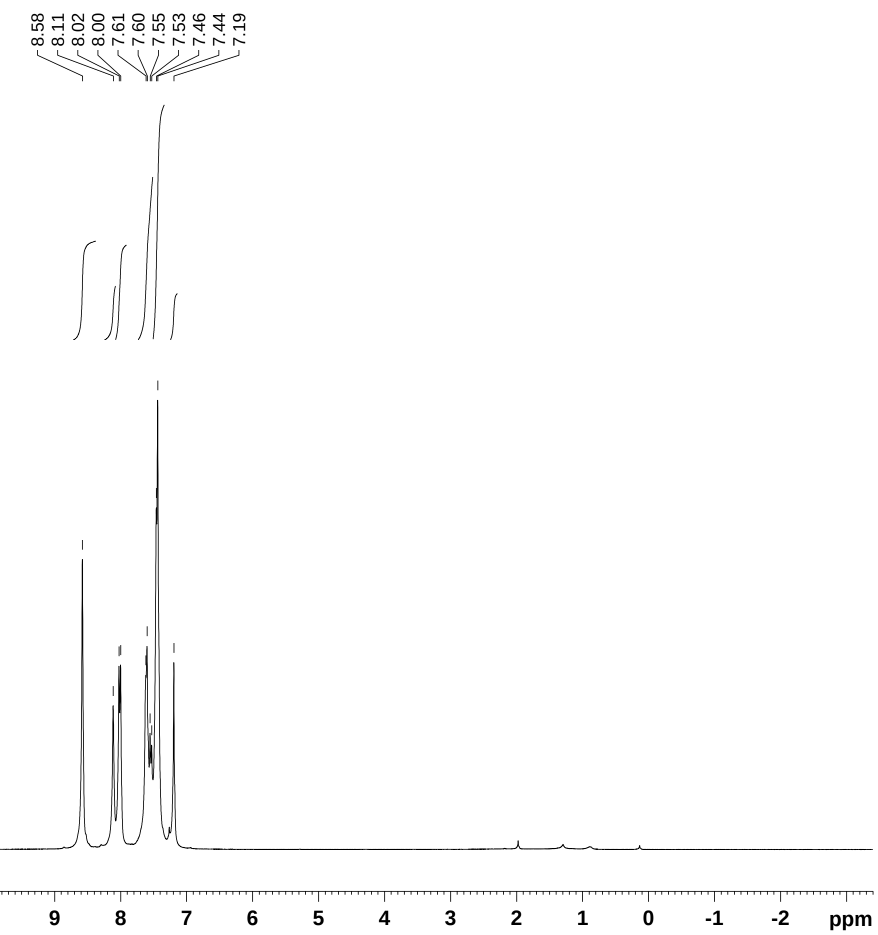
8.58
8.11
8.02
8.00
7.61
7.60
7.55
7.53
7.46
7.44
7.19
14 13 12 11 10 9 8 7 6 5 4 3 2 1 0 -1 -2 ppm

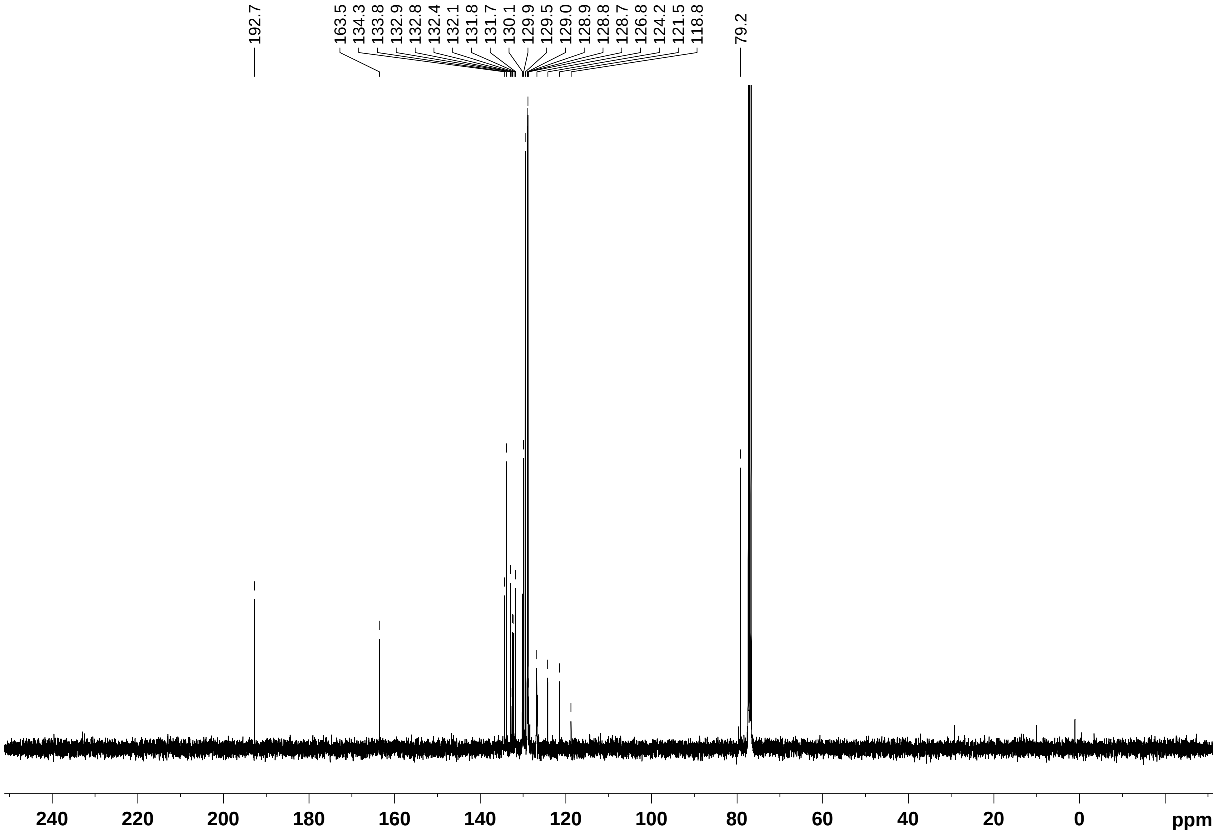
192.7
163.5
134.3
133.8
132.9
132.8
132.4
132.1
131.8
131.7
130.1
129.9
129.5
129.0
128.9
128.8
128.7
126.8
124.2
121.5
118.8
79.2
240 220 200 180 160 140 120 100 80 60 40 20 0 ppm

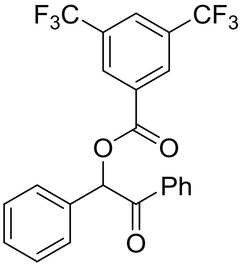

3k

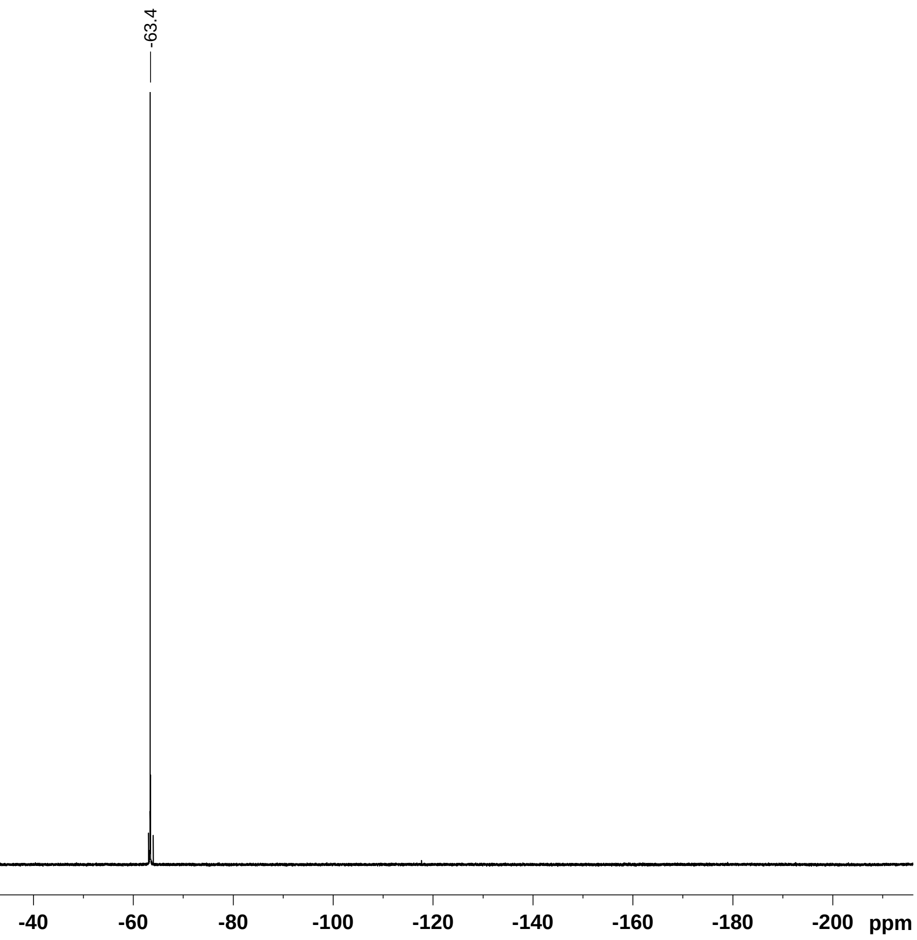

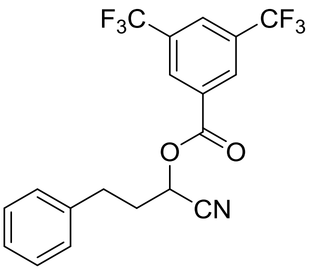
F_3C
CF_3
O
O
CN

6a

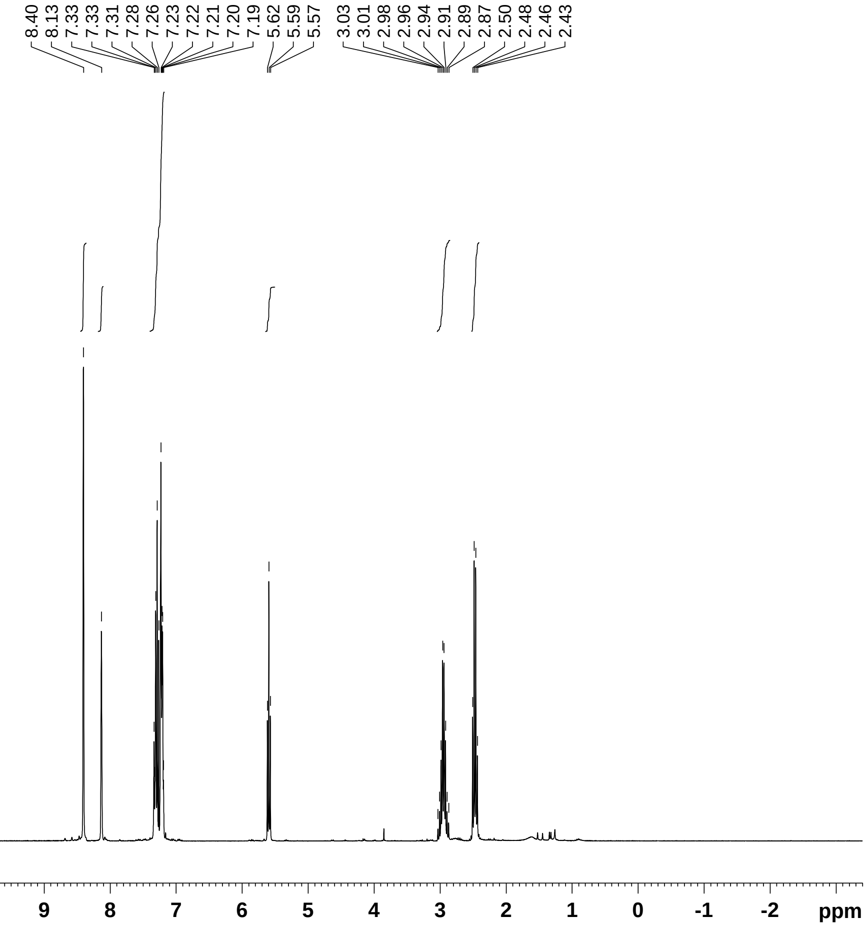
8.40
8.13
7.33
7.33
7.31
7.28
7.26
7.23
7.22
7.21
7.20
7.19
5.62
5.59
5.57
3.03
3.01
2.98
2.96
2.94
2.91
2.89
2.87
2.50
2.48
2.46
2.43
14 13 12 11 10 9 8 7 6 5 4 3 2 1 0 -1 -2 ppm

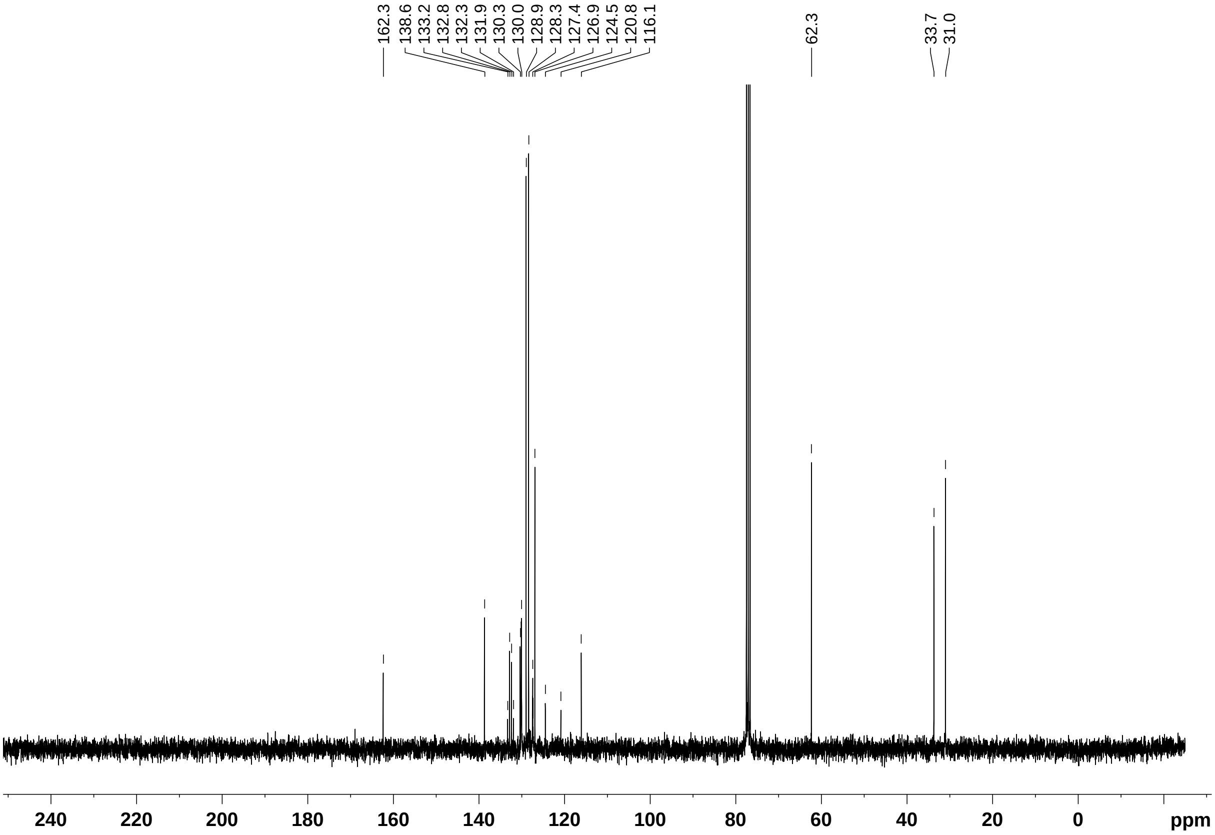
162.3
138.6
133.2
132.8
132.3
131.9
130.3
130.0
128.9
128.3
127.4
126.9
124.5
120.8
116.1
62.3
33.7
31.0
240 220 200 180 160 140 120 100 80 60 40 20 0 ppm

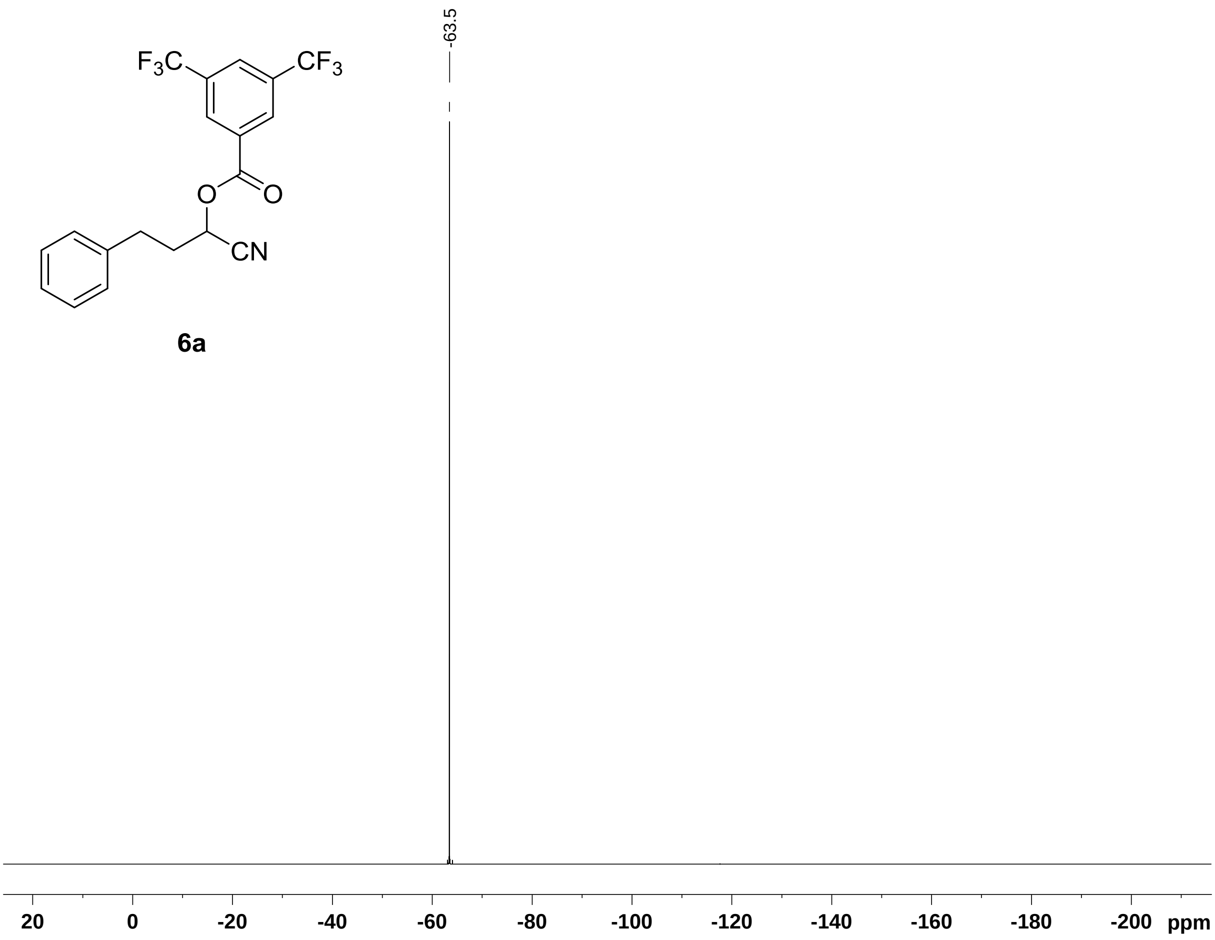

F3C
CF3
O
O
CN
6a
-63.5
20
0
-20
-40
-60
-80
-100
-120
-140
-160
-180
-200
ppm

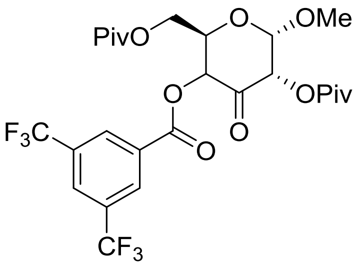
PivO
O
OMe
OPiv
O
O
O
F3C
CF3

6b

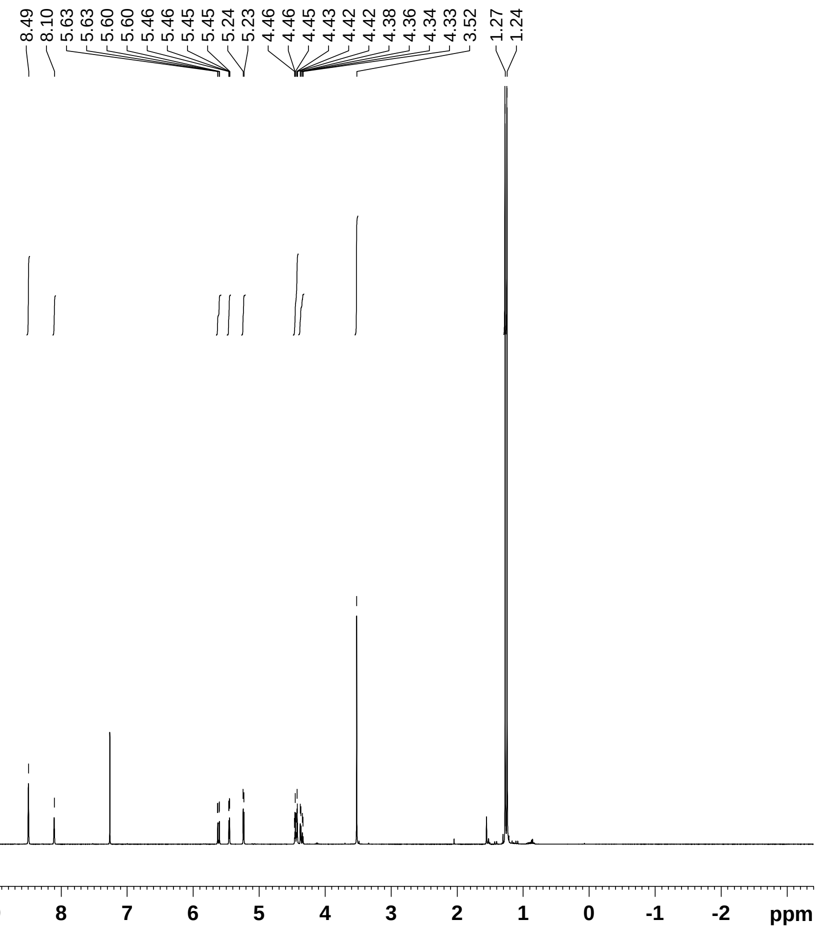
8.49
8.10
5.63
5.63
5.60
5.60
5.46
5.46
5.45
5.45
5.24
5.23
4.46
4.46
4.45
4.43
4.42
4.42
4.38
4.36
4.34
4.33
3.52
1.27
1.24
14 13 12 11 10 9 8 7 6 5 4 3 2 1 0 -1 -2 ppm

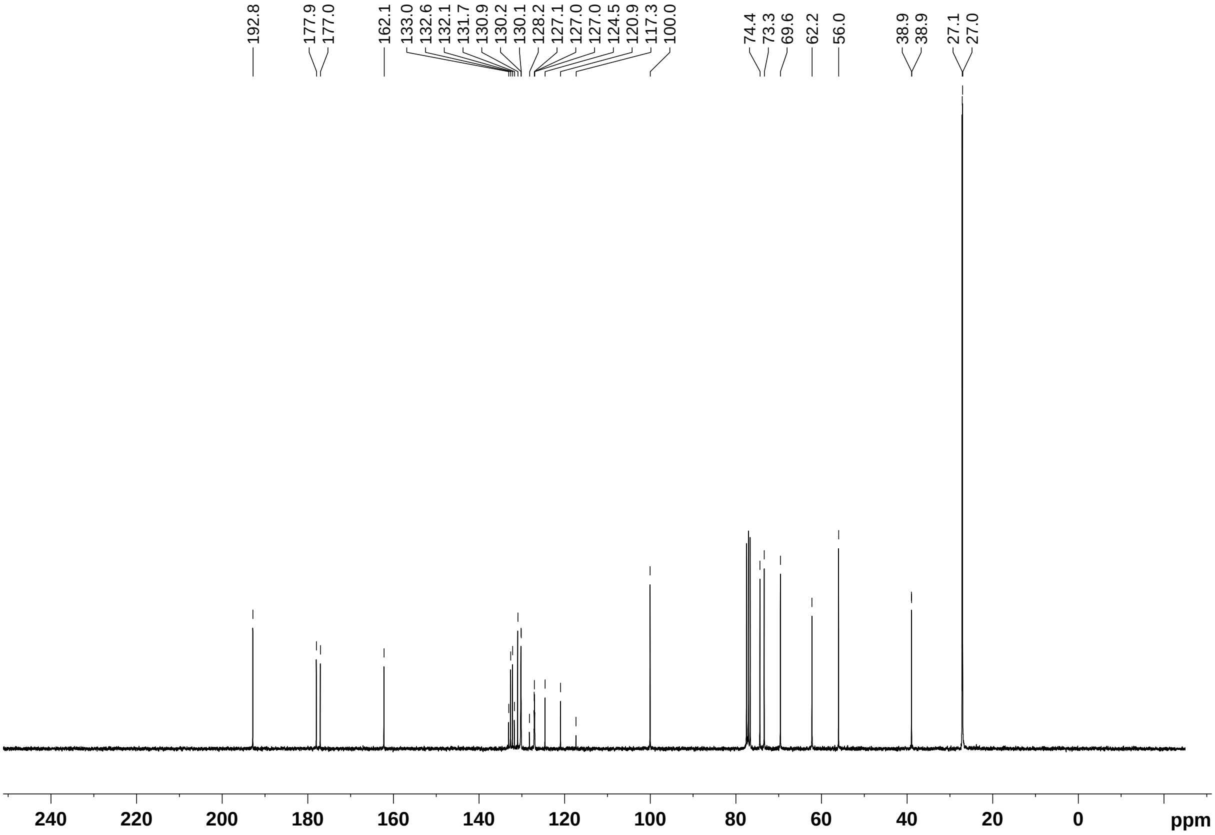
192.8
177.9
177.0
162.1
133.0
132.6
132.1
131.7
130.9
130.2
130.1
128.2
127.1
127.0
124.5
120.9
117.3
100.0
74.4
73.3
69.6
62.2
56.0
38.9
38.9
27.1
27.0
240 220 200 180 160 140 120 100 80 60 40 20 0 ppm

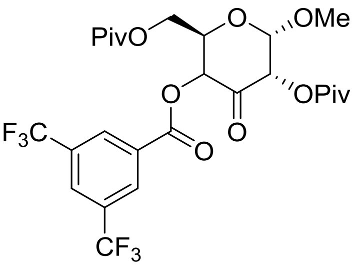
PivO
O
OMe
O
OPiv
O
F3C
O
CF3

6b

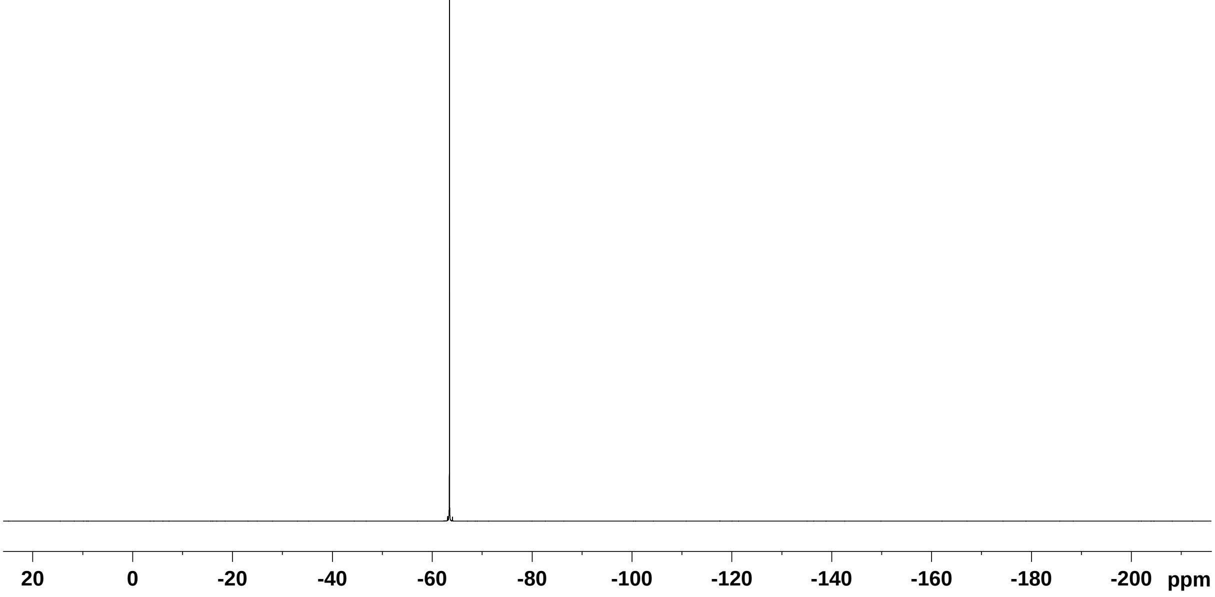
-63.5
20
0
-20
-40
-60
-80
-100
-120
-140
-160
-180
-200
ppm

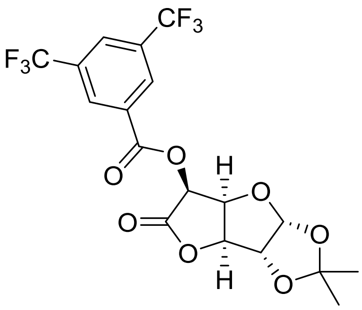
CF3
F3C
O
O
H
O
O
O
O
H
O

6c

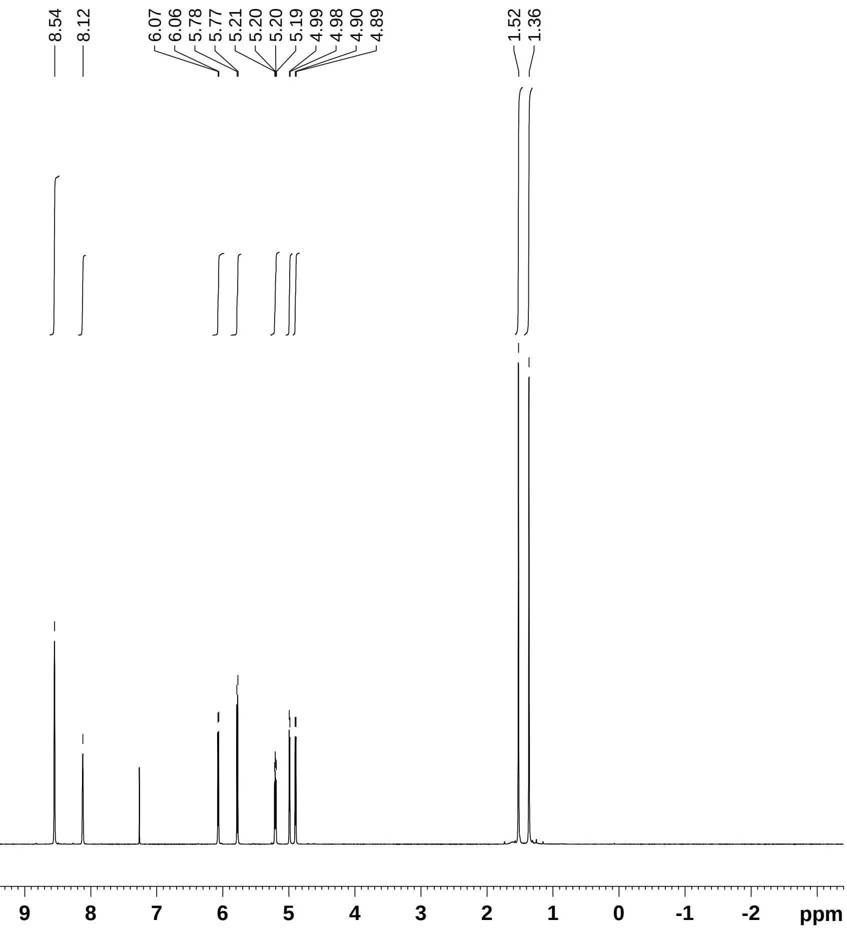
8.54
8.12
6.07
6.06
5.78
5.77
5.21
5.20
5.20
5.19
4.99
4.98
4.90
4.89
1.52
1.36
14 13 12 11 10 9 8 7 6 5 4 3 2 1 0 -1 -2 ppm

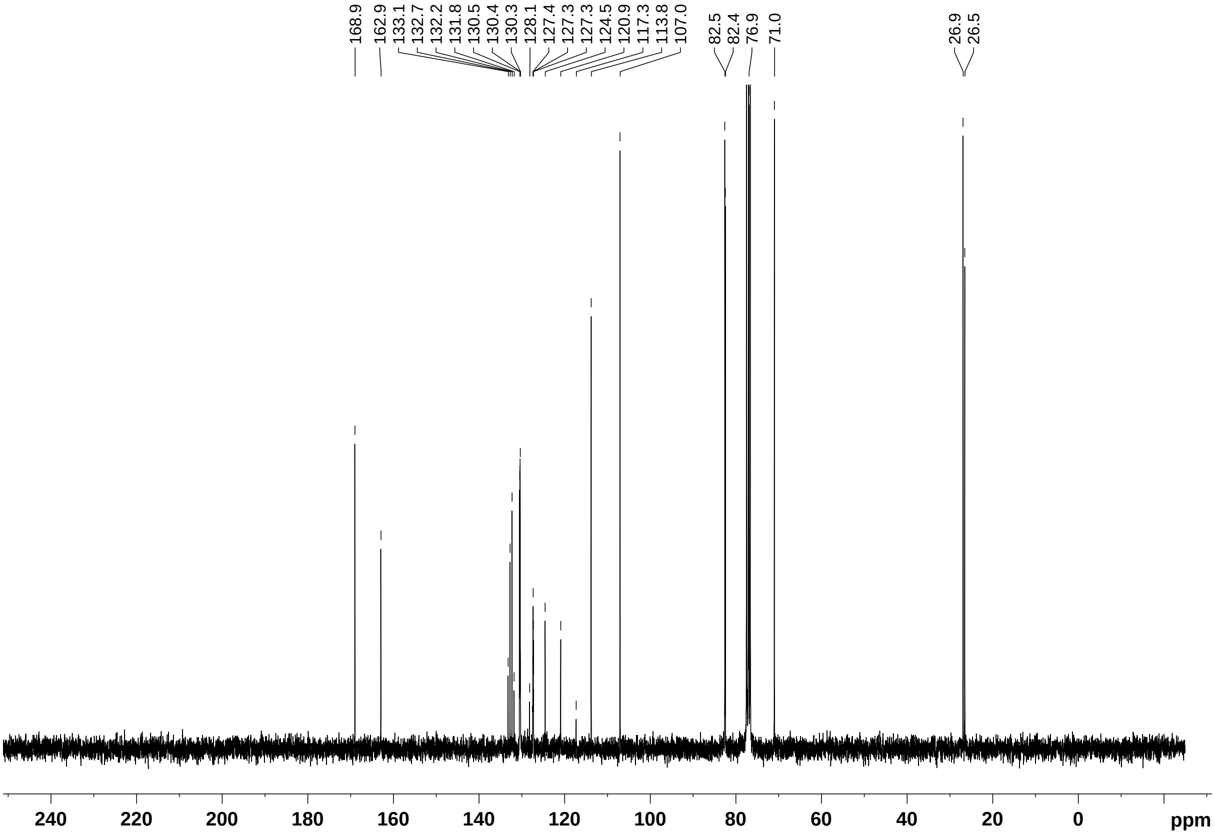
168.9
162.9
133.1
132.7
132.2
131.8
130.5
130.4
130.3
128.1
127.4
127.3
127.3
124.5
120.9
117.3
113.8
107.0
82.5
82.4
76.9
71.0
26.9
26.5
240 220 200 180 160 140 120 100 80 60 40 20 0 ppm

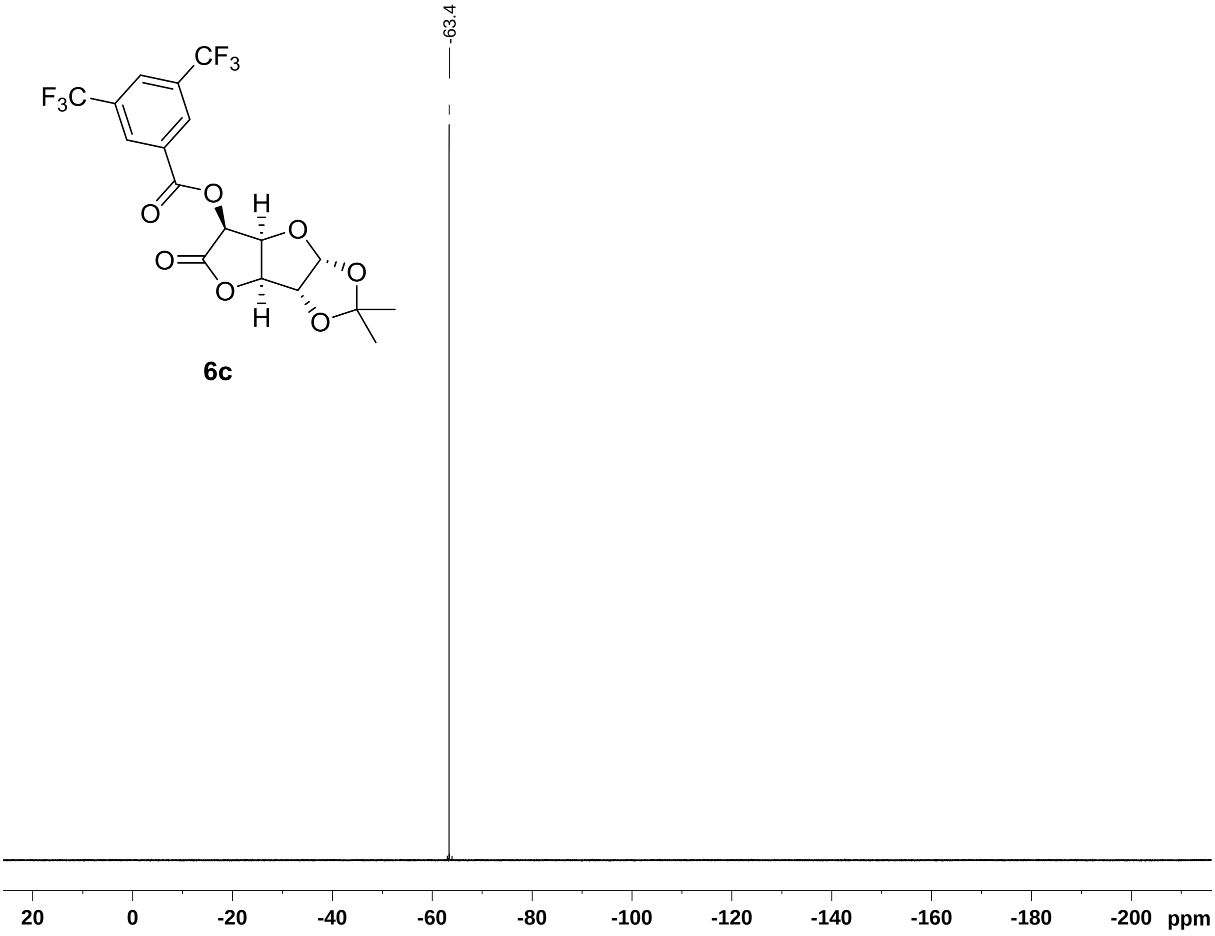
CF3
F3C
O
O
H
O
O
O
O
H
O
O
-63.4
20
0
-20
-40
-60
-80
-100
-120
-140
-160
-180
-200
ppm

6c

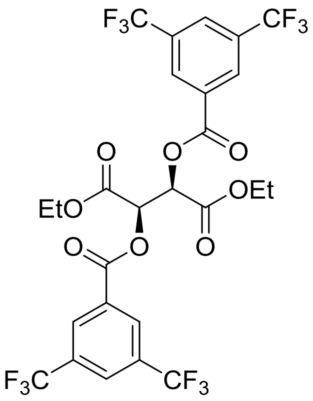
F3C
CF3
O
O
O
OEt
EtO
O
O
O
O
O
F3C
CF3

6d

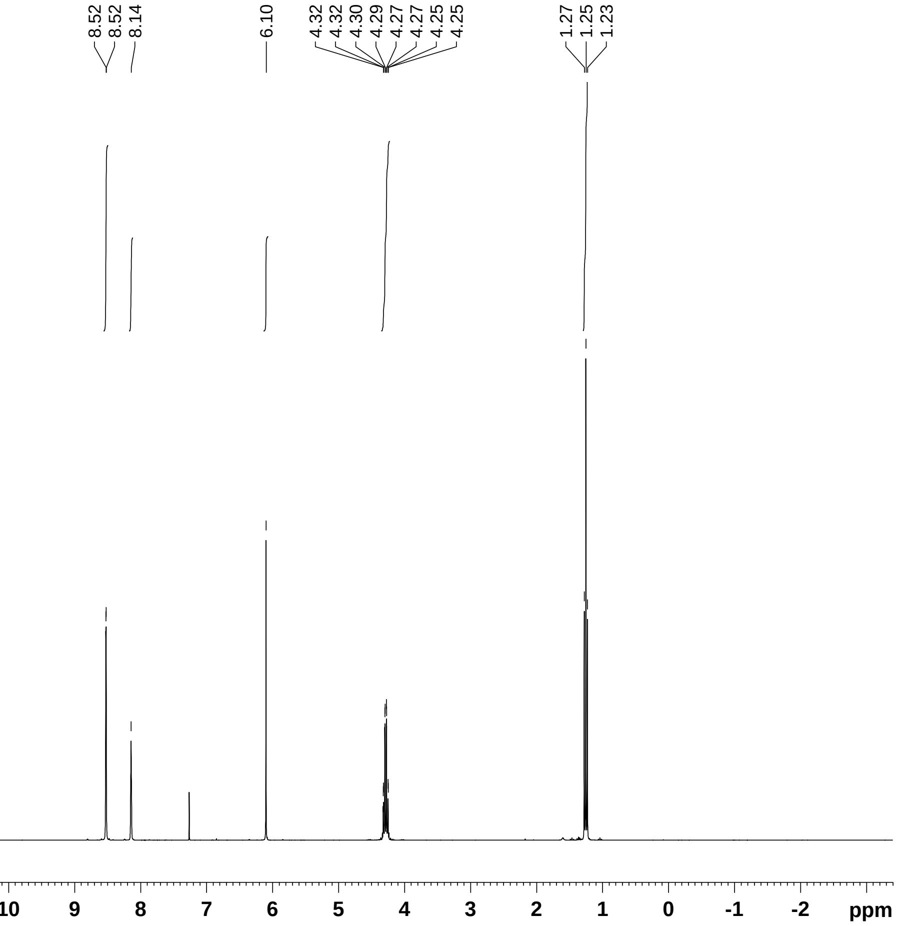
8.52
8.52
8.14
6.10
4.32
4.32
4.30
4.29
4.27
4.27
4.25
4.25
1.27
1.25
1.23
14 13 12 11 10 9 8 7 6 5 4 3 2 1 0 -1 -2 ppm

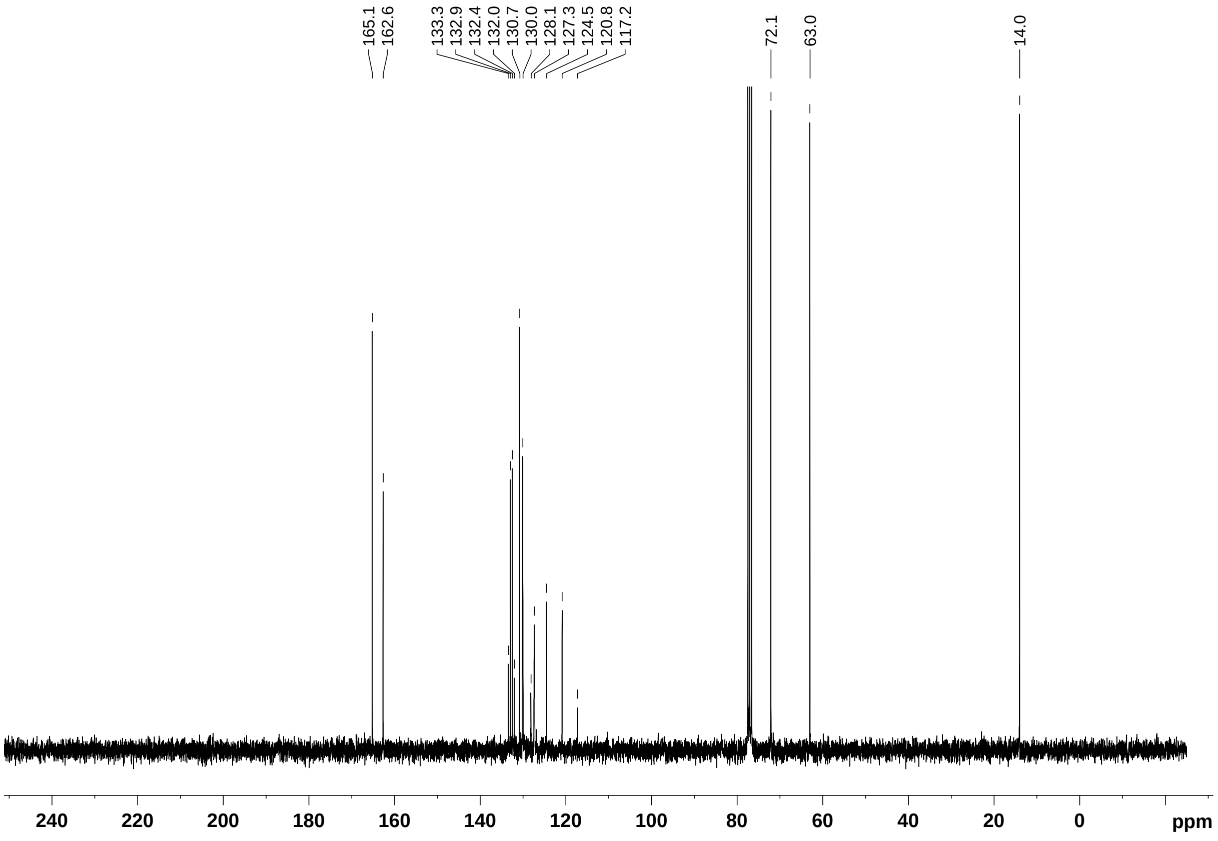
165.1
162.6
133.3
132.9
132.4
132.0
130.7
130.0
128.1
127.3
124.5
120.8
117.2
72.1
63.0
14.0
240 220 200 180 160 140 120 100 80 60 40 20 0 ppm

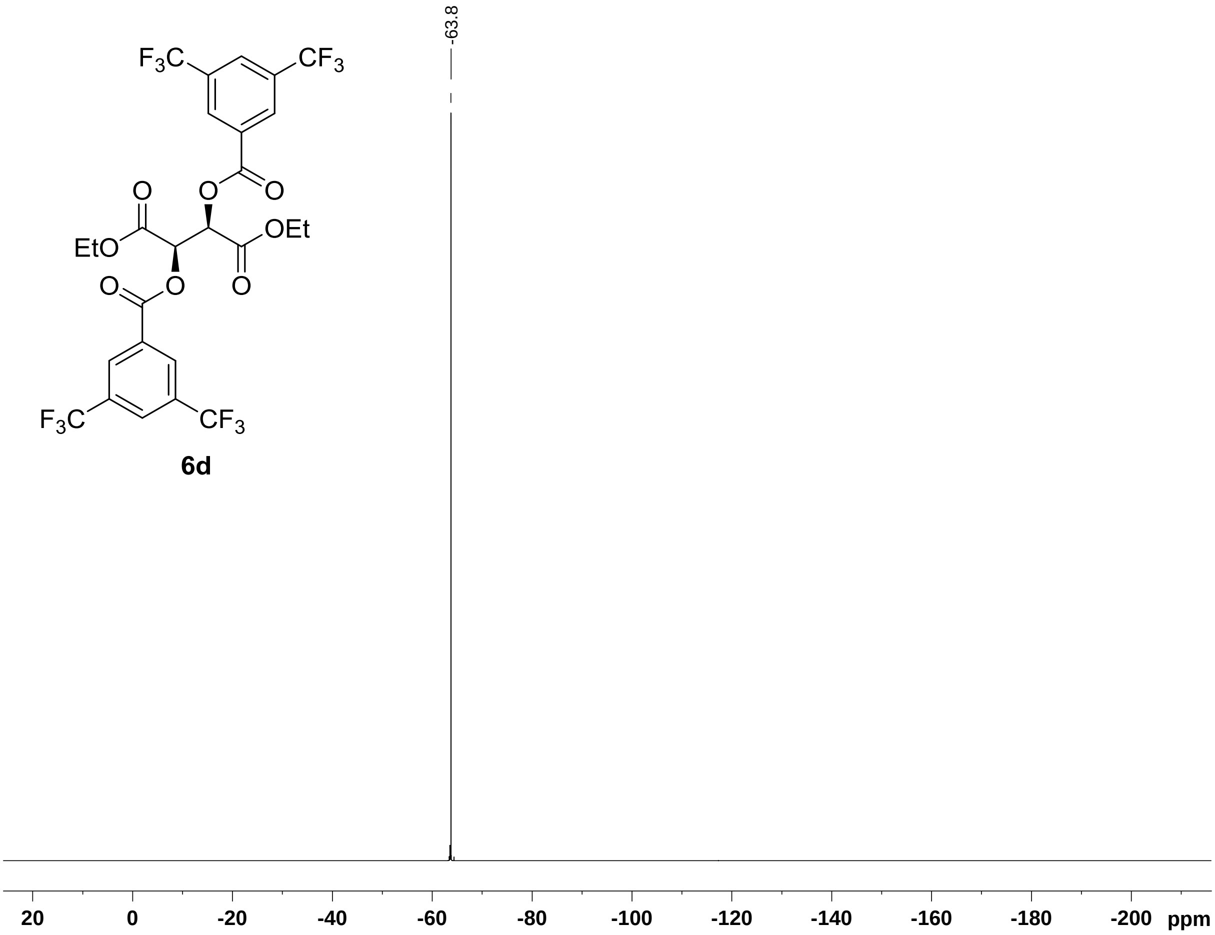
F3C
CF3
O
O
O
EtO
OEt
O
O
O
F3C
CF3
-63.8
20
0
-20
-40
-60
-80
-100
-120
-140
-160
-180
-200
ppm

6d

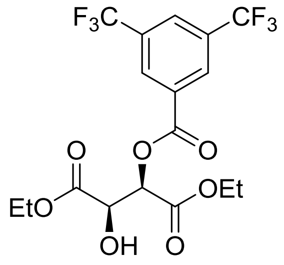
F3C
CF3
O
O
O
OEt
EtO
OH
O

6e

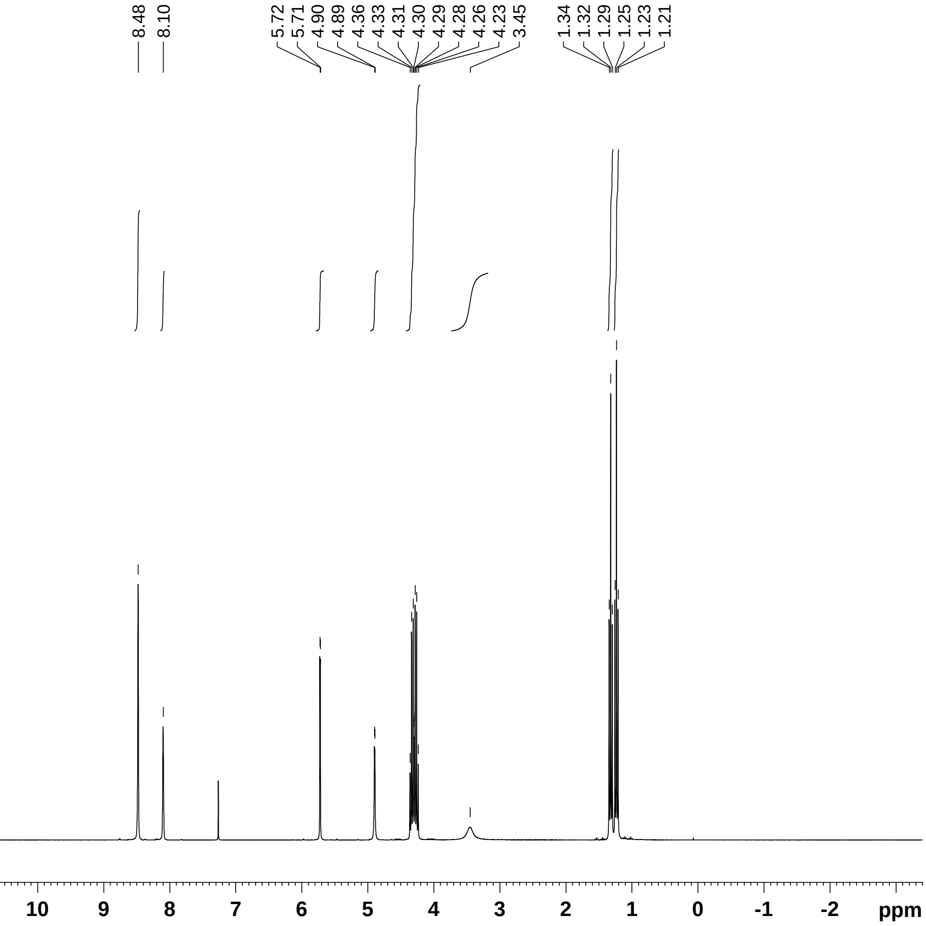
8.48
8.10
5.72
5.71
4.90
4.89
4.36
4.33
4.31
4.30
4.29
4.28
4.26
4.23
3.45
1.34
1.32
1.29
1.25
1.23
1.21
14 13 12 11 10 9 8 7 6 5 4 3 2 1 0 -1 -2 ppm

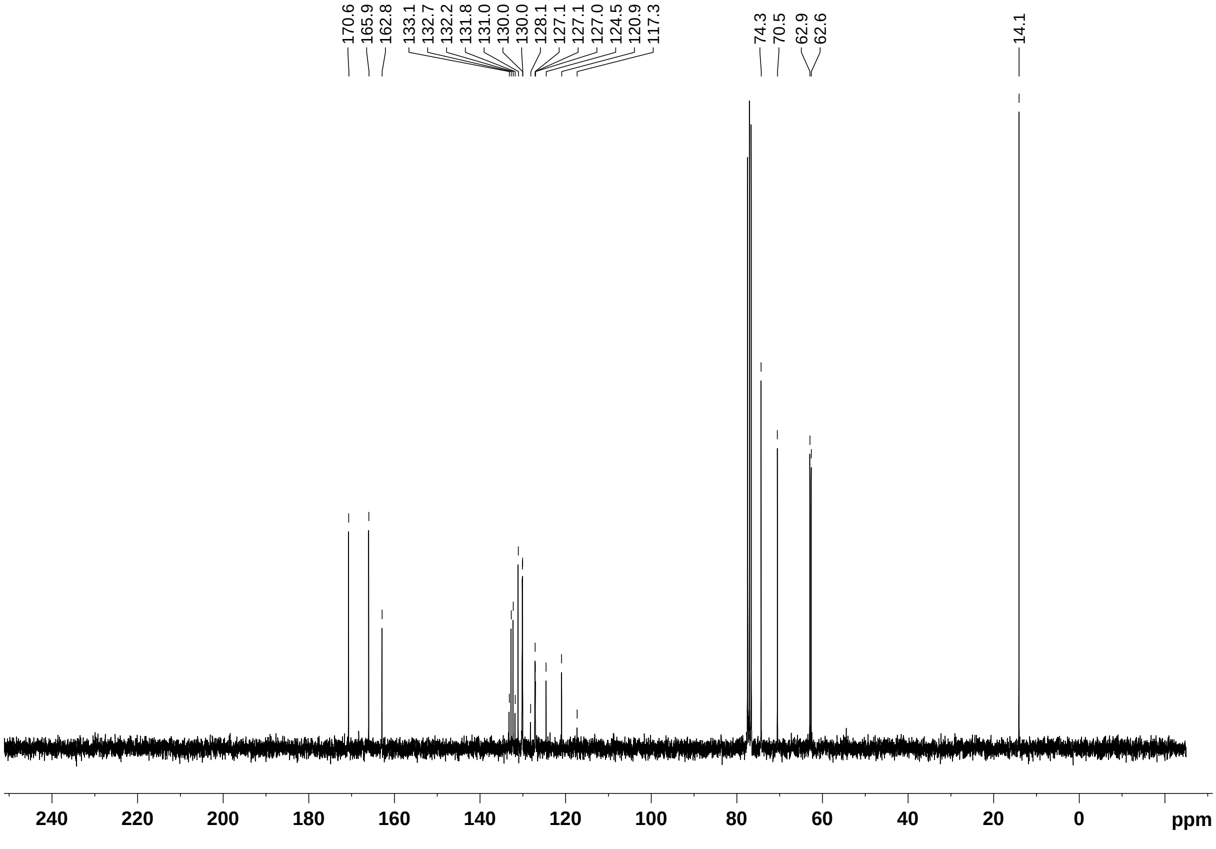
170.6
165.9
162.8
133.1
132.7
132.2
131.8
131.0
130.0
130.0
128.1
127.1
127.1
127.0
124.5
120.9
117.3
74.3
70.5
62.9
62.6
14.1
240 220 200 180 160 140 120 100 80 60 40 20 0 ppm

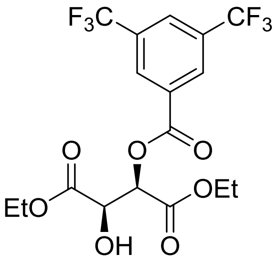
F3C
CF3
O
O
O
EtO
OEt
OH
O

6e

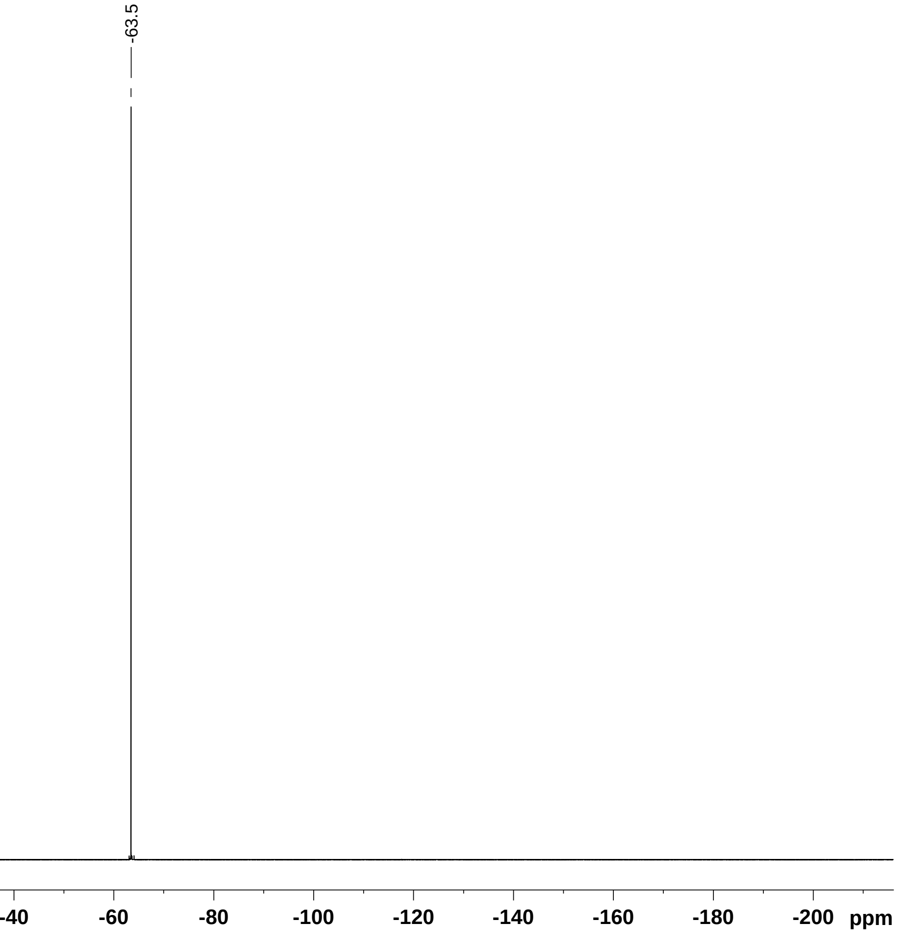
-63.5
20
0
-20
-40
-60
-80
-100
-120
-140
-160
-180
-200
ppm

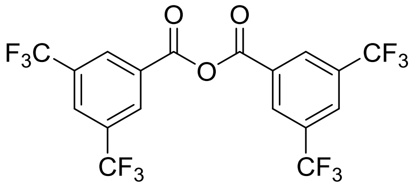
F3C
O
O
O
CF3
CF3
CF3

9

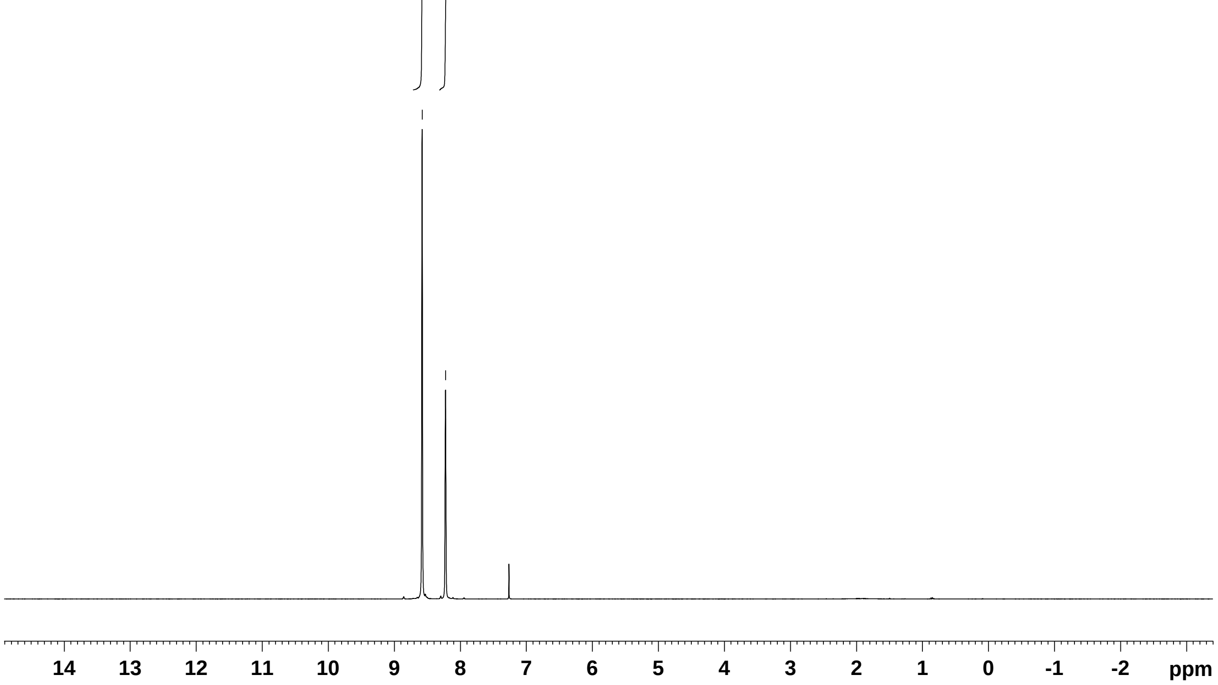
8.58
8.22
14 13 12 11 10 9 8 7 6 5 4 3 2 1 0 -1 -2 ppm

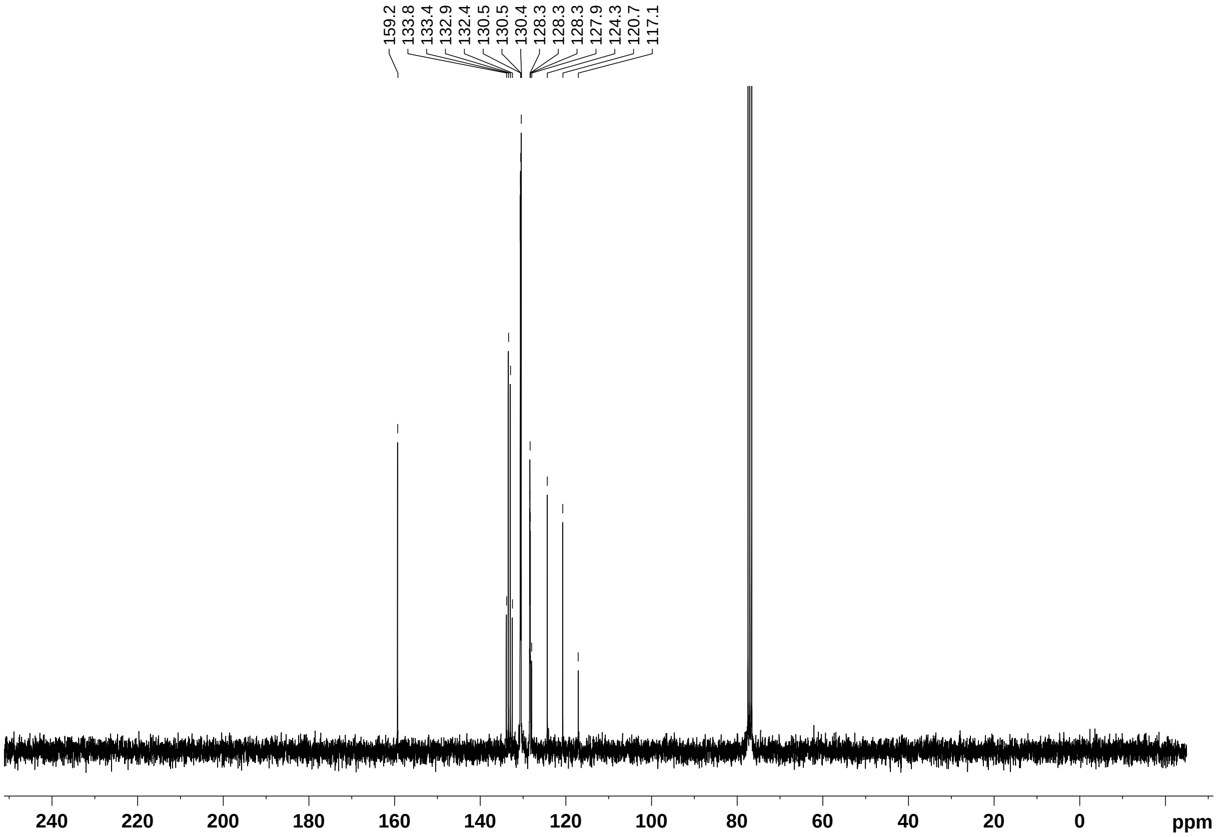
159.2
133.8
133.4
132.9
132.4
130.5
130.5
130.4
128.3
128.3
128.3
127.9
124.3
120.7
117.1
240 220 200 180 160 140 120 100 80 60 40 20 0 ppm

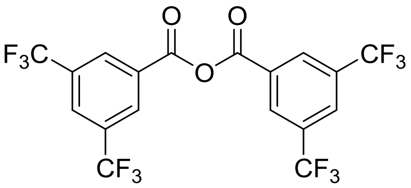

F3C
O
O
O
CF3
CF3
CF3

9

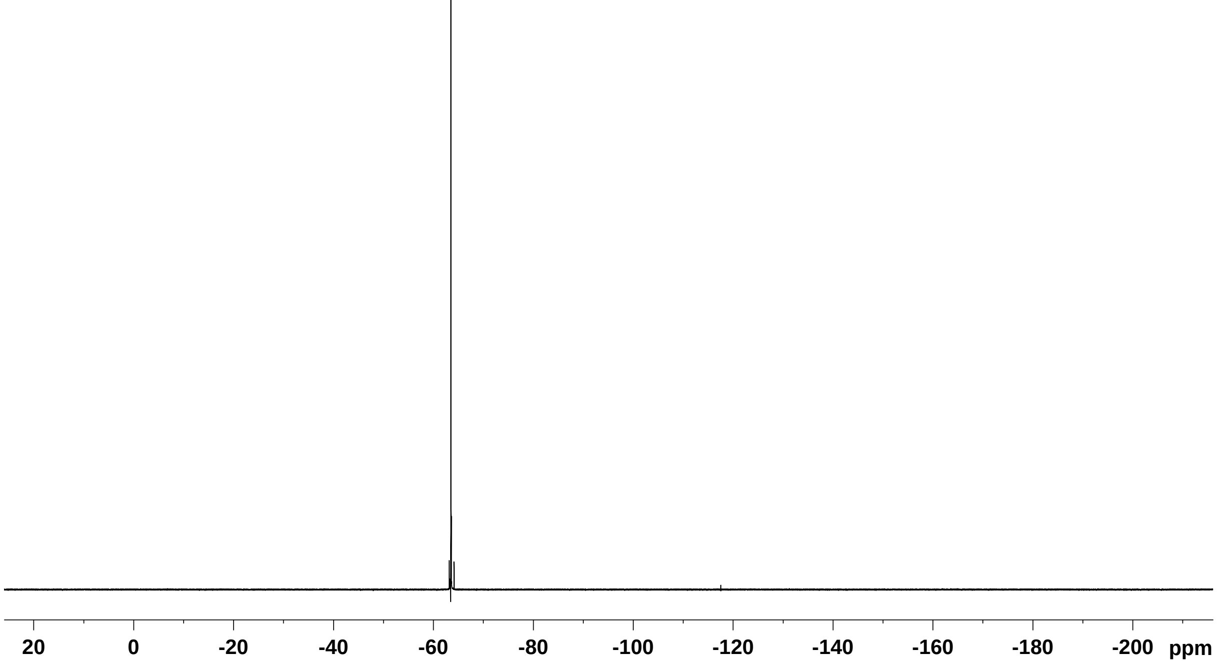

-63.6
20
0
-20
-40
-60
-80
-100
-120
-140
-160
-180
-200
ppm

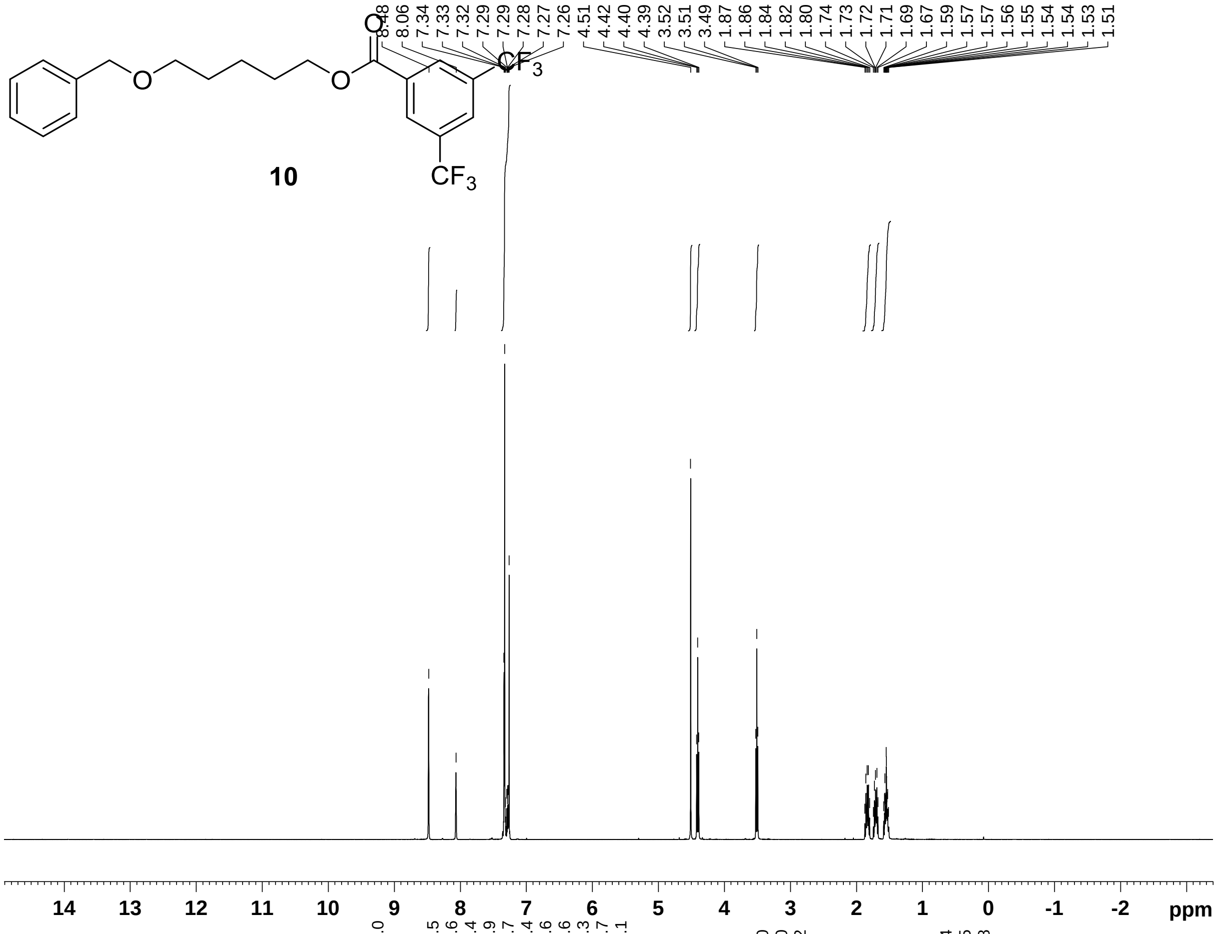

10
O
O
O
CF3
CF3
8.48
8.06
7.34
7.33
7.32
7.29
7.29
7.28
7.27
7.26
4.51
4.42
4.40
4.39
3.52
3.51
3.49
1.87
1.86
1.84
1.82
1.80
1.74
1.73
1.72
1.71
1.69
1.67
1.59
1.57
1.57
1.56
1.55
1.54
1.53
1.51
14 13 12 11 10 9 8 7 6 5 4 3 2 1 0 -1 -2 ppm

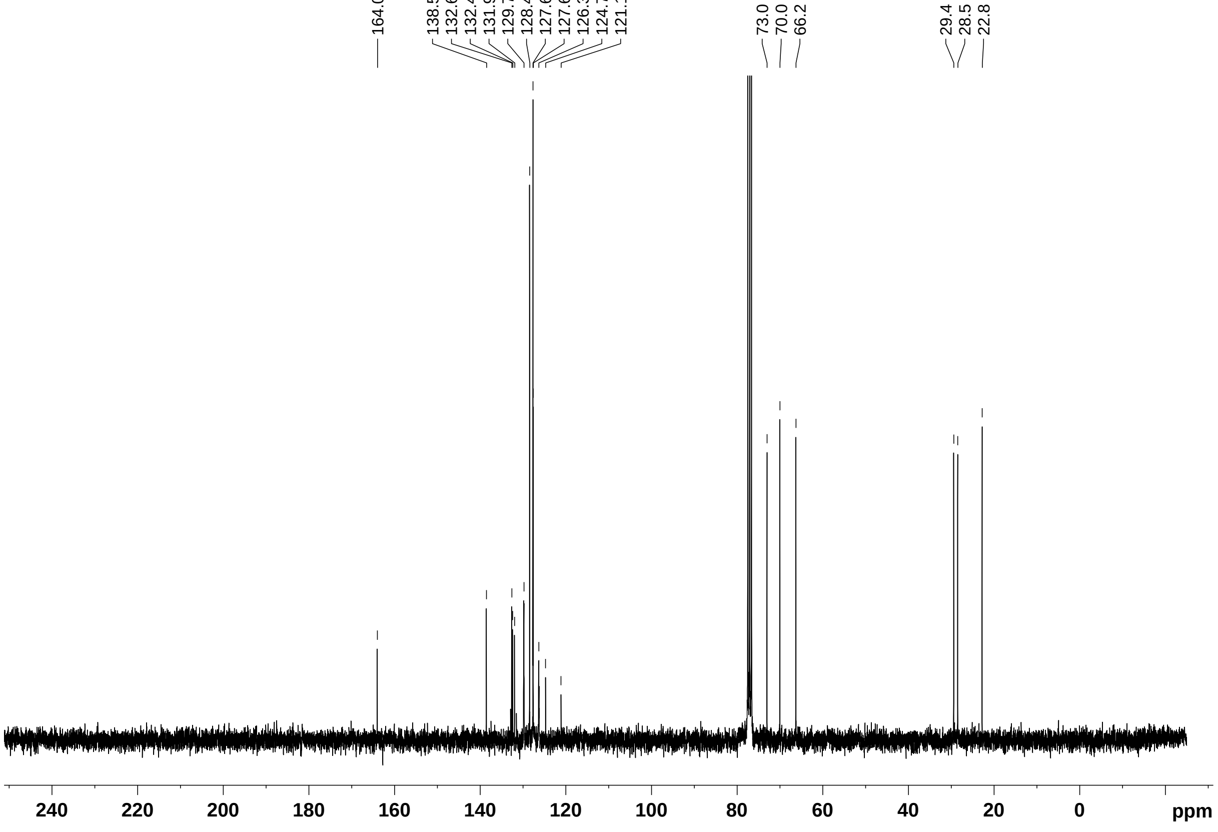

164.0
138.5
132.6
132.4
131.9
129.7
128.4
127.6
127.6
126.3
124.7
121.1
73.0
70.0
66.2
29.4
28.5
22.8
240 220 200 180 160 140 120 100 80 60 40 20 0 ppm

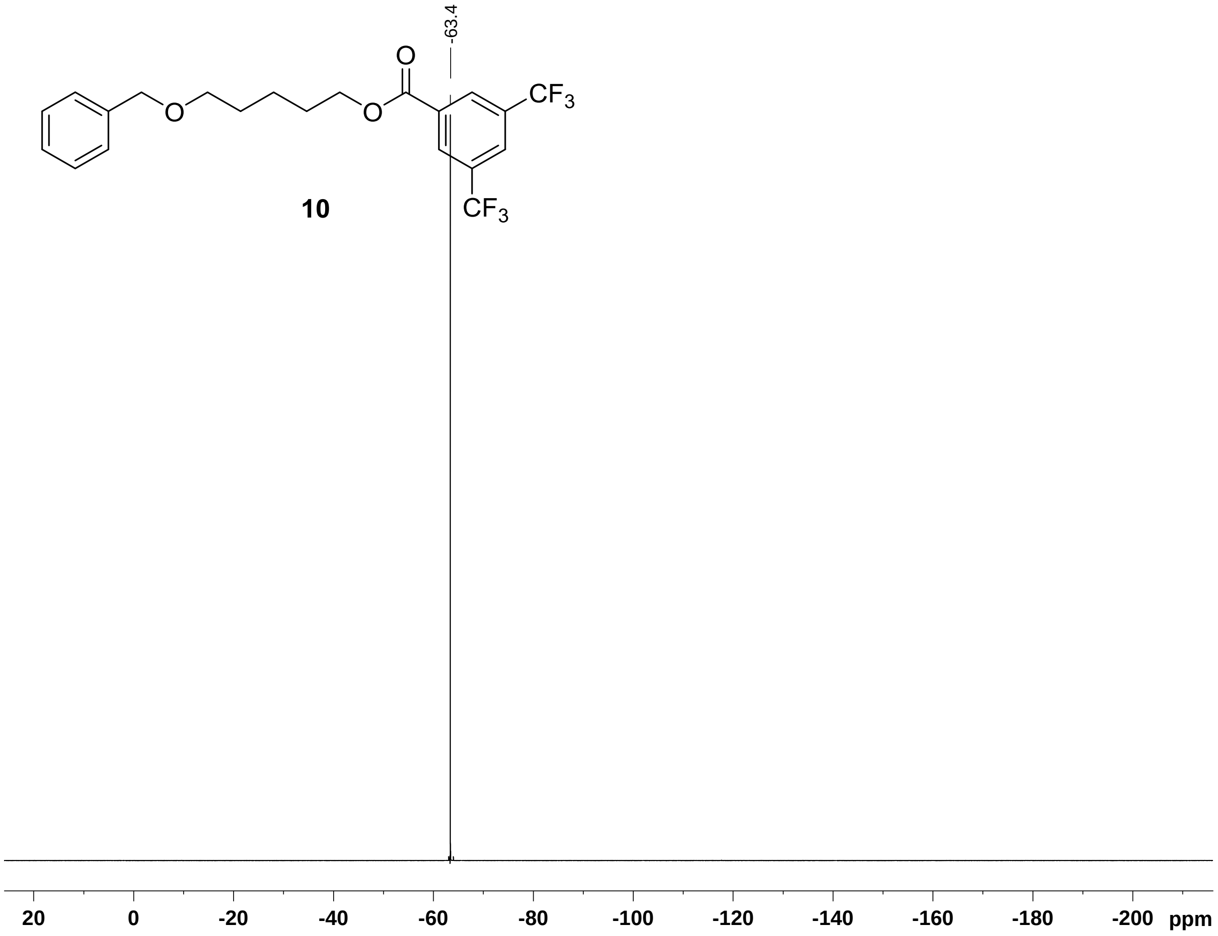
-63.4
O
O
O
CF3
CF3
10
20 0 -20 -40 -60 -80 -100 -120 -140 -160 -180 -200 ppm

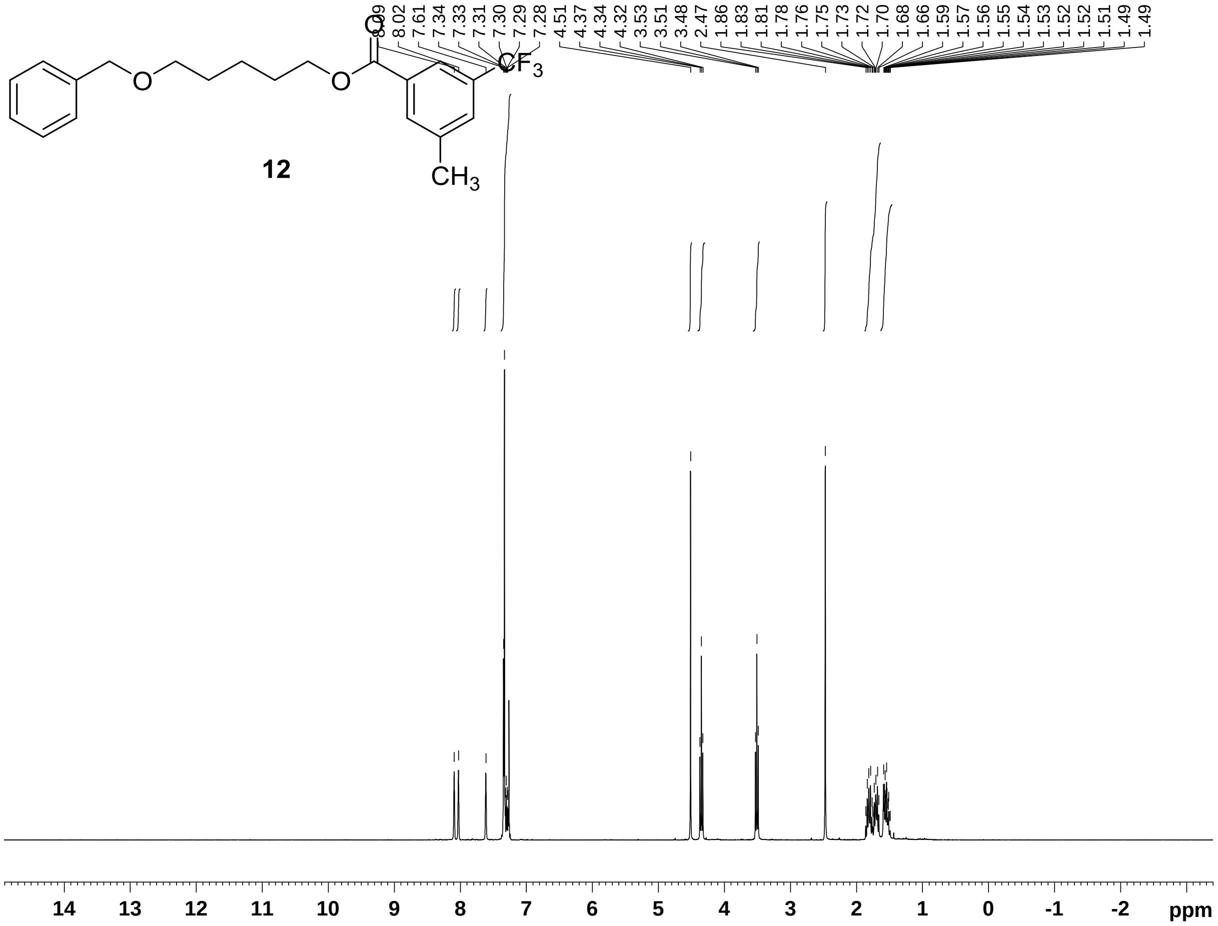
O
O
O
CF3
CH3
8.09 8.02 7.61 7.34 7.33 7.31 7.30 7.29 7.28 4.51 4.37 4.34 4.32 3.53 3.51 3.48 2.47 1.86 1.83 1.81 1.78 1.76 1.75 1.73 1.72 1.70 1.68 1.66 1.59 1.57 1.56 1.55 1.54 1.53 1.52 1.52 1.51 1.49 1.49
14 13 12 11 10 9 8 7 6 5 4 3 2 1 0 -1 -2 ppm

12

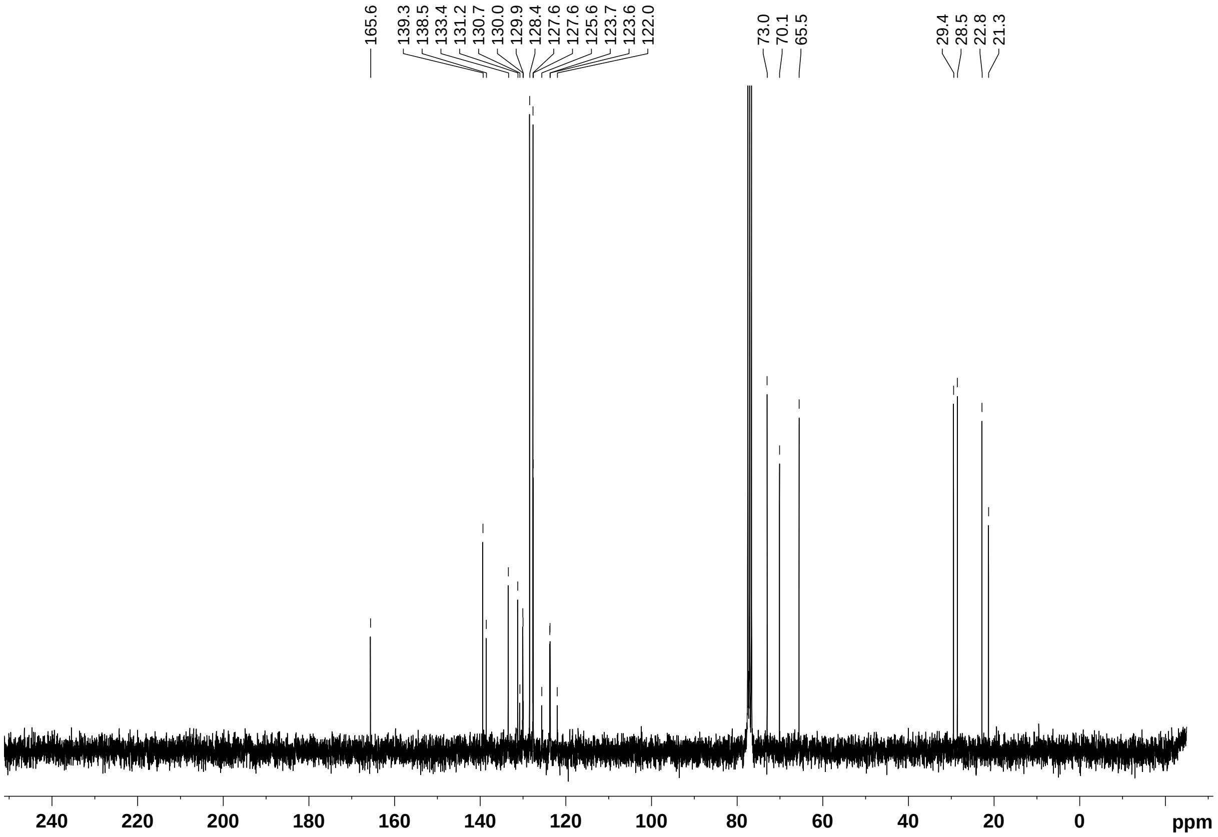
165.6
139.3 138.5 133.4 131.2 130.7 130.0 129.9 128.4 127.6 127.6 125.6 123.7 123.6 122.0
73.0 70.1 65.5
29.4 28.5 22.8 21.3
240 220 200 180 160 140 120 100 80 60 40 20 0 ppm

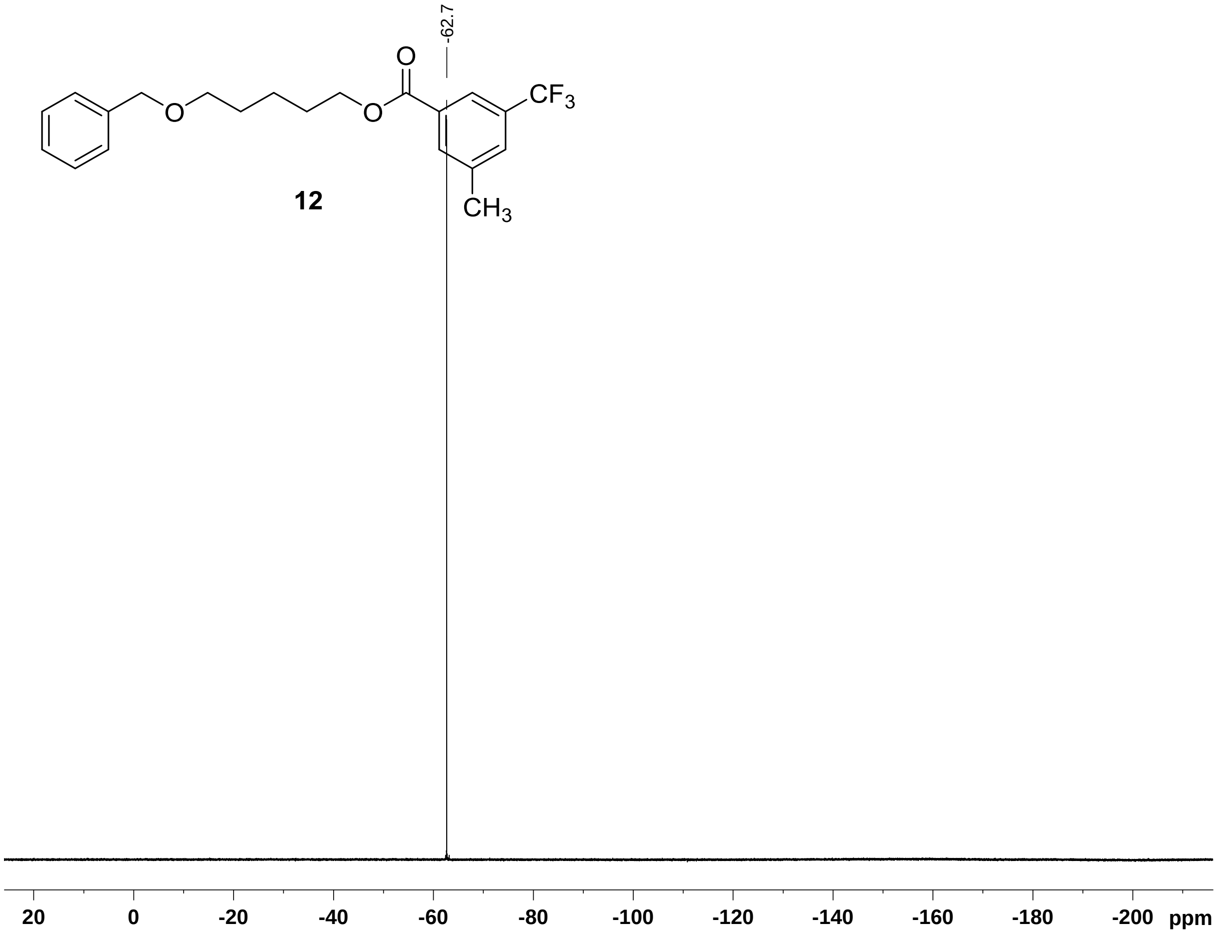

12

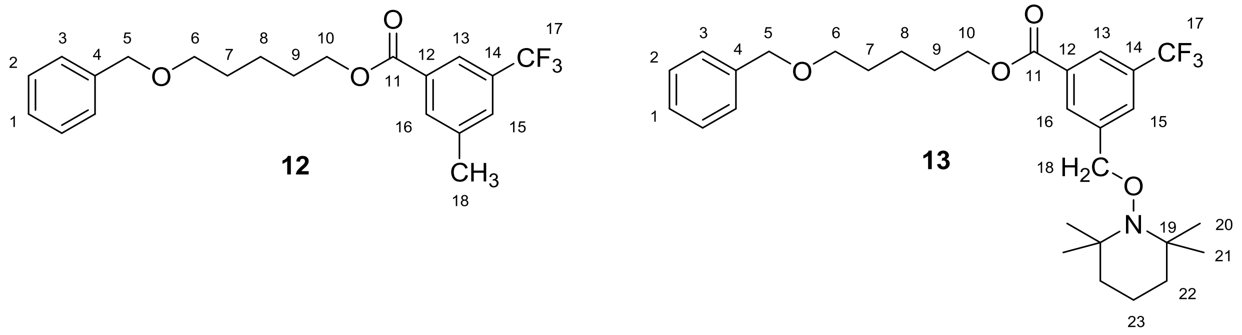
12
13
1
2
3
4
5
6
7
8
9
10
11
12
13
14
15
16
17
18
19
20
21
22
23
CF3
CH3
H2C
O
N

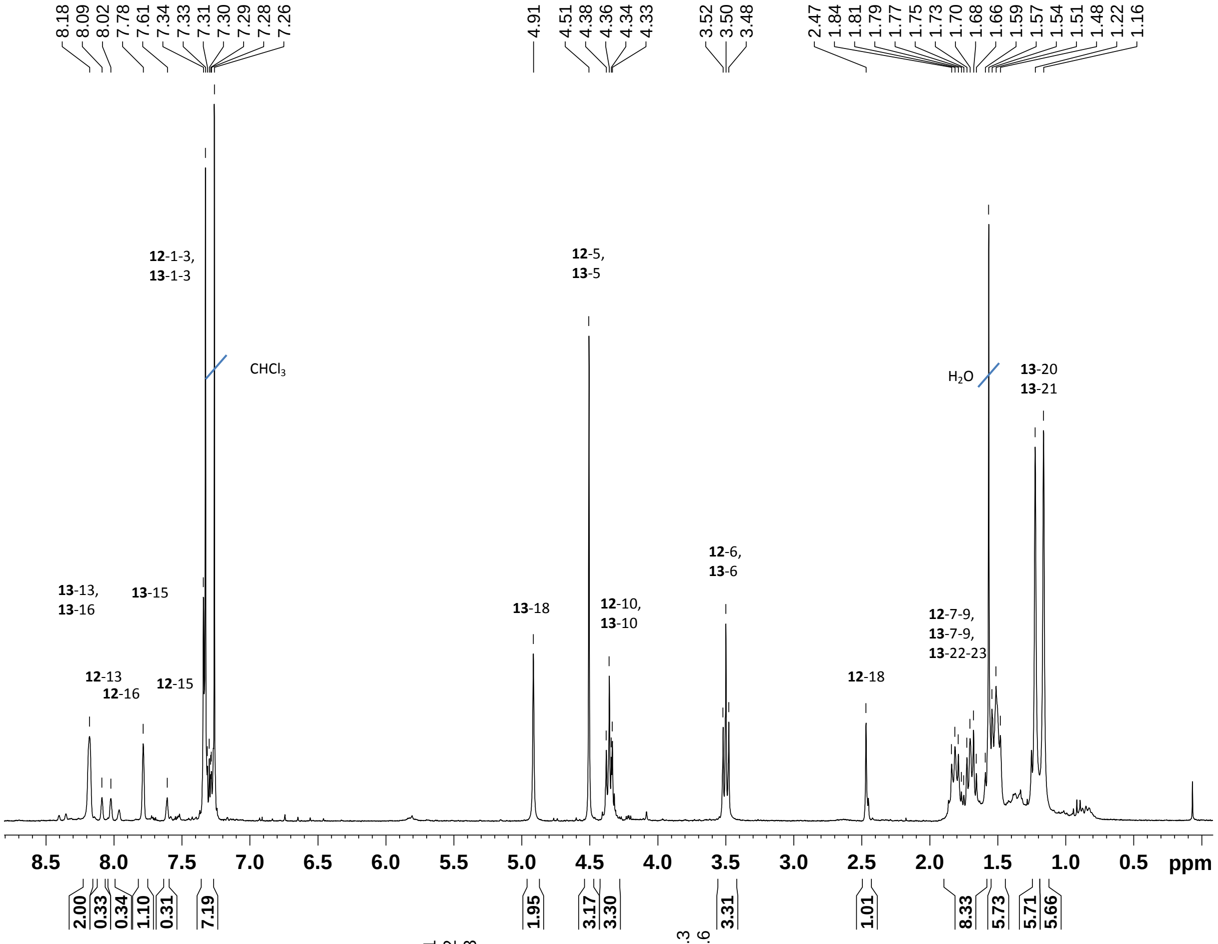
8.18
8.09
8.02
7.78
7.61
7.34
7.33
7.31
7.30
7.29
7.28
7.26
4.91
4.51
4.38
4.36
4.34
4.33
3.52
3.50
3.48
2.47
1.84
1.81
1.79
1.77
1.75
1.73
1.70
1.68
1.66
1.59
1.57
1.54
1.51
1.48
1.22
1.16
12-1-3,
13-1-3
CHCl3
12-5,
13-5
H2O
13-20
13-21
13-13,
13-16
13-15
12-13
12-16
12-15
13-18
12-10,
13-10
12-6,
13-6
12-7-9,
13-7-9,
13-22-23
12-18
8.5
8.0
7.5
7.0
6.5
6.0
5.5
5.0
4.5
4.0
3.5
3.0
2.5
2.0
1.5
1.0
0.5
ppm
2.00
0.33
0.34
1.10
0.31
7.19
1.95
3.17
3.30
3.31
1.01
8.33
5.73
5.71
5.66

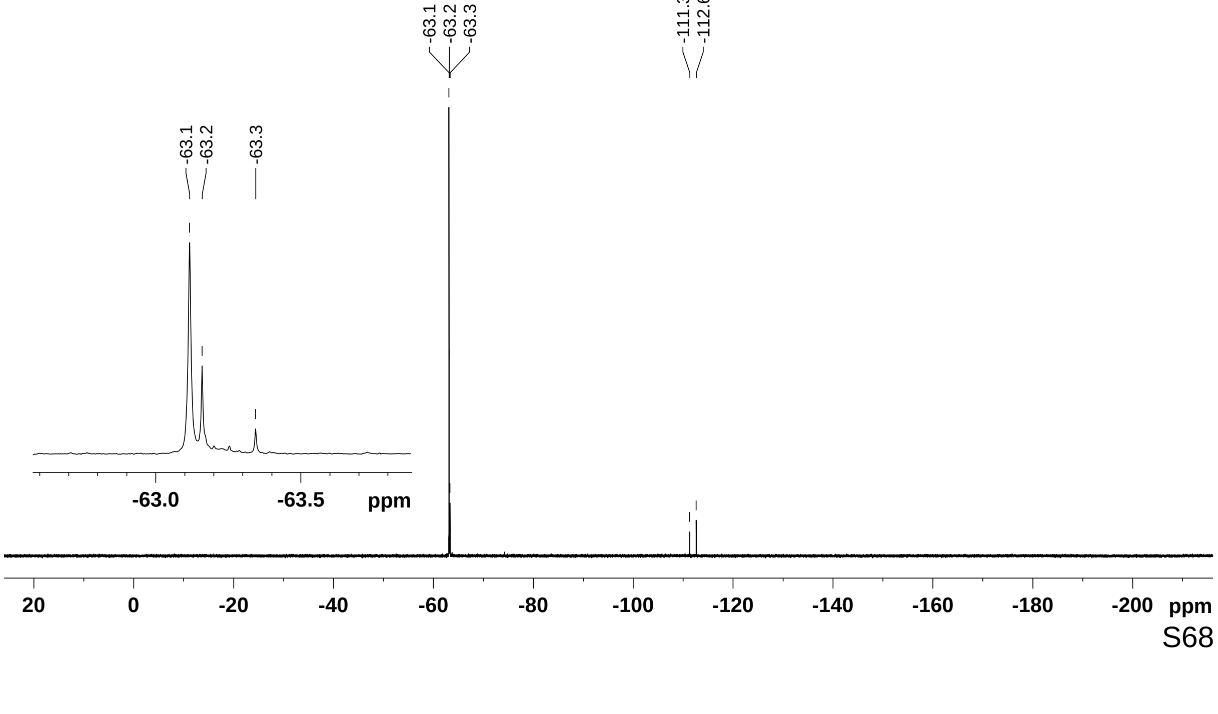
-63.1
-63.2
-63.3
-111.3
-112.6
-63.0
-63.5
ppm
20
0
-20
-40
-60
-80
-100
-120
-140
-160
-180
-200
